# Multiancestry conditional and joint analysis applied to GWAS summary statistics

**DOI:** 10.1038/s41588-026-02799-1

**Published:** 2026-10-20

**Authors:** Xiaotong Wang, Yong Wang, Jian Yang, Peter M. Visscher, Naomi R. Wray, Loic Yengo

**Affiliations:** 1 Department of Psychiatry, University of Oxford, Oxford, UK; 2 Pioneer Centre for SMARTbiomed, University of Oxford, Oxford, UK; 3 New Cornerstone Science Laboratory, School of Life Sciences, Westlake University, Hangzhou, China; 4 Westlake Laboratory of Life Sciences and Biomedicine, Hangzhou, China; 5 Institute for Molecular Bioscience, The University of Queensland, Brisbane, Queensland Australia; 6 Nuffield Department of Population Health, University of Oxford, Oxford, UK

**Keywords:** Genetics, Software

## Abstract

Conditional and joint (COJO) analysis of genome-wide association study (GWAS) data is a standard post-GWAS analysis for identifying statistically independent associations. Although GWAS meta-analyses are increasingly conducted across multiple ancestry groups, the optimal strategy for multiancestry COJO analysis remains unclear. Here we introduce Manc-COJO, a method for multiancestry COJO analysis. Simulations and real-data analyses show that Manc-COJO improves detection of independent association signals while reducing false positives compared with single-ancestry COJO implemented in genome-wide complex trait analysis (GCTA) and its ad-hoc multiancestry adaptations. We also introduce Manc-COJO:FIX, a follow-up algorithm to identify ancestry-specific independent associations. The C++ implementation of Manc-COJO is over 120-fold faster than GCTA-COJO and supports linkage disequilibrium references derived from either individual-level genotype data or pre-computed matrices, facilitating analyses when data sharing is limited.

## Abstract (web summary)

This study introduces Manc-COJO, a method for detecting independent genetic associations by integrating data from multiple ancestries, reducing false positives and enabling fast, flexible analyses across diverse populations.

## Main

Genome-wide association studies (GWASs) have revolutionized understanding of the genetic architecture of complex traits^1^. Typically, the GWAS paradigm tests for association between a trait and single-nucleotide polymorphisms (SNPs), one at a time (giving marginal SNP effect estimates). However, associations detected in GWASs are often not independent because of linkage disequilibrium (LD) between SNPs. In principle, multivariate linear regression analysis can be applied to GWAS data to identify secondary SNP associations, which are SNP associations detected after conditioning on one or more significantly associated SNPs. However, such analyses are computationally demanding and are not possible in modern GWASs which are typically meta-analyses of summary statistics from contributing studies, without sharing of individual-level data. To address this challenge, the conditional and joint (COJO) multiple-SNP analysis of GWAS summary statistics was developed and implemented into the genome-wide complex trait analysis (GCTA) software^2^. The algorithm requires an LD matrix to model the correlation structure between SNPs that the software generates from user-provided, individual-level genotype data. Notably, the GCTA-COJO algorithm was developed with an implicit assumption that the GWAS was conducted in a single-ancestry group.

GWASs are now commonly conducted as meta-analyses of GWASs across samples with diverse ancestries^3–5^. Ancestry groups differ in allele frequencies and LD patterns because of demographic and evolutionary processes such as random drift^6^. A key question when implementing post-GWAS analyses such as COJO is how to model the correlation structure between SNPs in a multiancestry GWAS setting. Some studies have repurposed GCTA-COJO to analyze multiancestry GWAS data either by using LD matrices derived from European ancestry cohorts^5,7^, because these dominate (typically >80%) current GWAS meta-analyses^8^, or by constructing LD matrices from reference samples that match the ancestry composition in the meta-analysis^9,10^. Other approaches include conducting COJO analysis separately within each ancestry^11–13^ or performing COJO analysis in one ancestry and attempting replication in other ancestries^14,15^. However, to our knowledge, these ad-hoc approaches have not been formally evaluated and optimal strategies require further consideration. Meanwhile, although GCTA-COJO was not originally designed as a fine-mapping tool, its ease of use has led many researchers to incorporate COJO into their fine-mapping pipeline^16,17^. However, interpretation of COJO-based independent associations as being causal merits further investigation.

In this study, we introduced the multiancestry conditional and joint analysis (Manc-COJO), which extends the capabilities of stepwise conditional and joint association analysis to multiancestry datasets. Through extensive simulations and real-data applications, we demonstrated that Manc-COJO improves the detection of independent trait-associated loci and reduces false-positive discoveries. An underlying assumption of our method (supported by an increasing body of evidence) was that causal effects of genetic variants are largely shared and concordant across ancestries^18–21^. We demonstrated that Manc-COJO is robust to violations of this assumption in scenarios involving ancestry-specific causal variants^22,23^. For that, we proposed a follow-up algorithm, Manc-COJO:FIX, which performs COJO analysis within a single ancestry while accounting for SNPs identified by Manc-COJO. It also provides additional functionality, including the incorporation of wet-lab-validated variants into the model.

## Results

### Overview of Manc-COJO

**Fig. 1:**
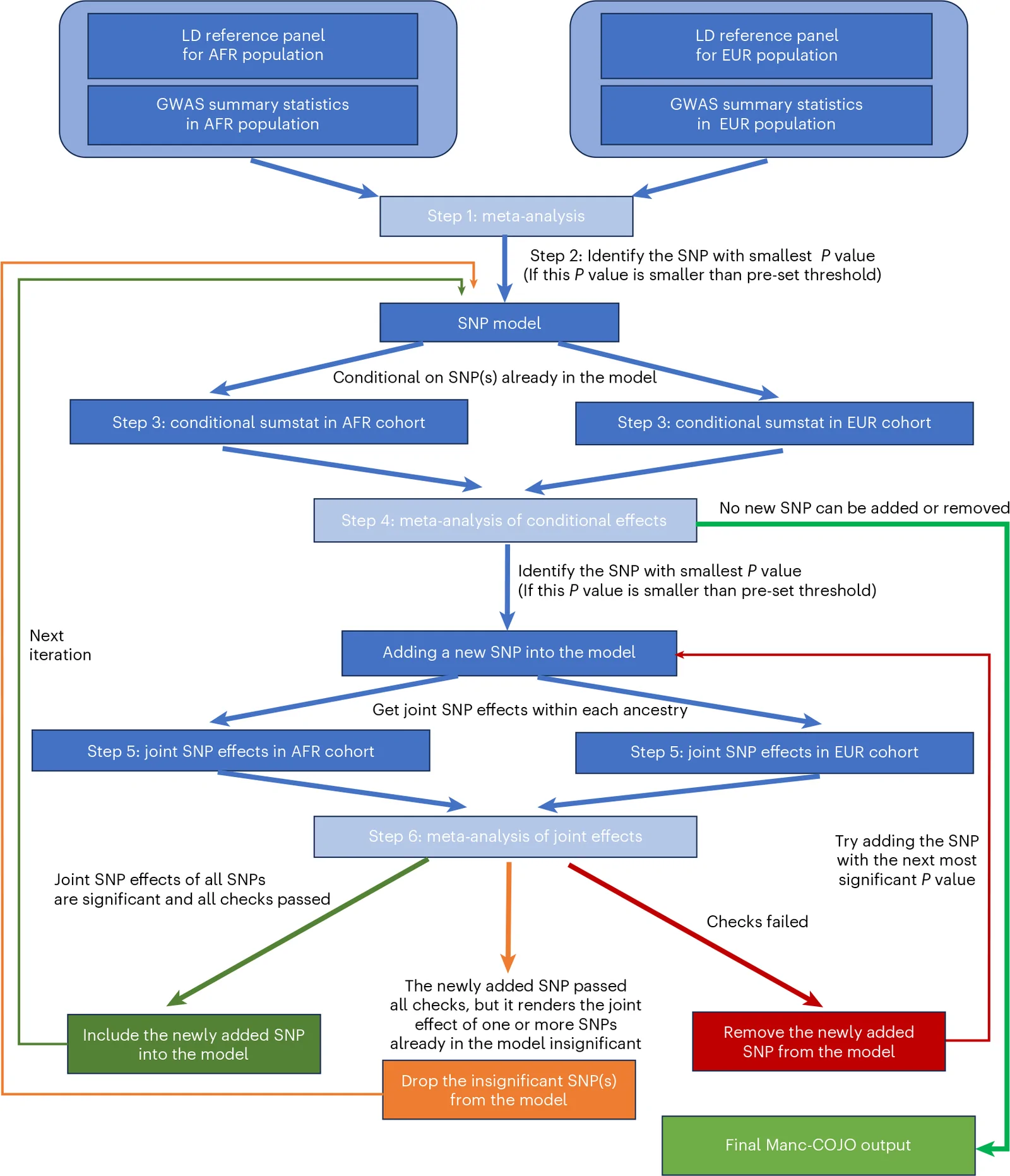
A schematic overview of the Manc-COJO algorithm. Manc-COJO is a forward and backward, stepwise conditional and joint analysis method that extends the current GCTA-COJO algorithm into multiancestry (illustrated here with two ancestry) settings.

Manc-COJO is a forward and backward, stepwise conditional and joint analysis method that extends the current GCTA-COJO algorithm into multiancestry settings. Illustrated in Fig. 1 for two ancestry groups, Manc-COJO utilizes GWAS summary statistics and LD reference matrices from each ancestry group as input. Manc-COJO is equivalent (when an in-sample LD matrix is used) to a stepwise-forward and stepwise-backward variable selection algorithm applied to individual-level genotypic data. Briefly, in each iteration, the top-associated SNPs are regressed out from the original phenotype within each ancestry group and the regression residuals serve as the new phenotype for a subsequent GWAS within each ancestry group (conditional analysis, conditioned on the top-associated SNPs). A new SNP is then selected for inclusion in the model based on *P* values from the meta-analysis of all GWASs (forward selection). After selection of a new SNP, all previously chosen SNPs, together with the newly selected SNP, are fitted jointly in a multiple linear regression model using the original phenotype within each ancestry group (joint analysis). The newly selected SNP is retained as a COJO-SNP based on the squared *z*-score value in the meta-analysis of joint effects and the adjusted *R*^2^ (coefficient of determination; an optional criterion) of the joint model within each ancestry group. If its inclusion renders previously included SNPs insignificant in the meta-analysis of joint effects, those insignificant SNPs are removed and the process continues (backward selection). This iterative process repeats until no further SNPs can be added or excluded from the model. Notably, the Manc-COJO software has been developed with an option to take LD matrices as reference input (as opposed to individual-level data to build the LD matrices).

### Simulation study results

**Fig. 2:**
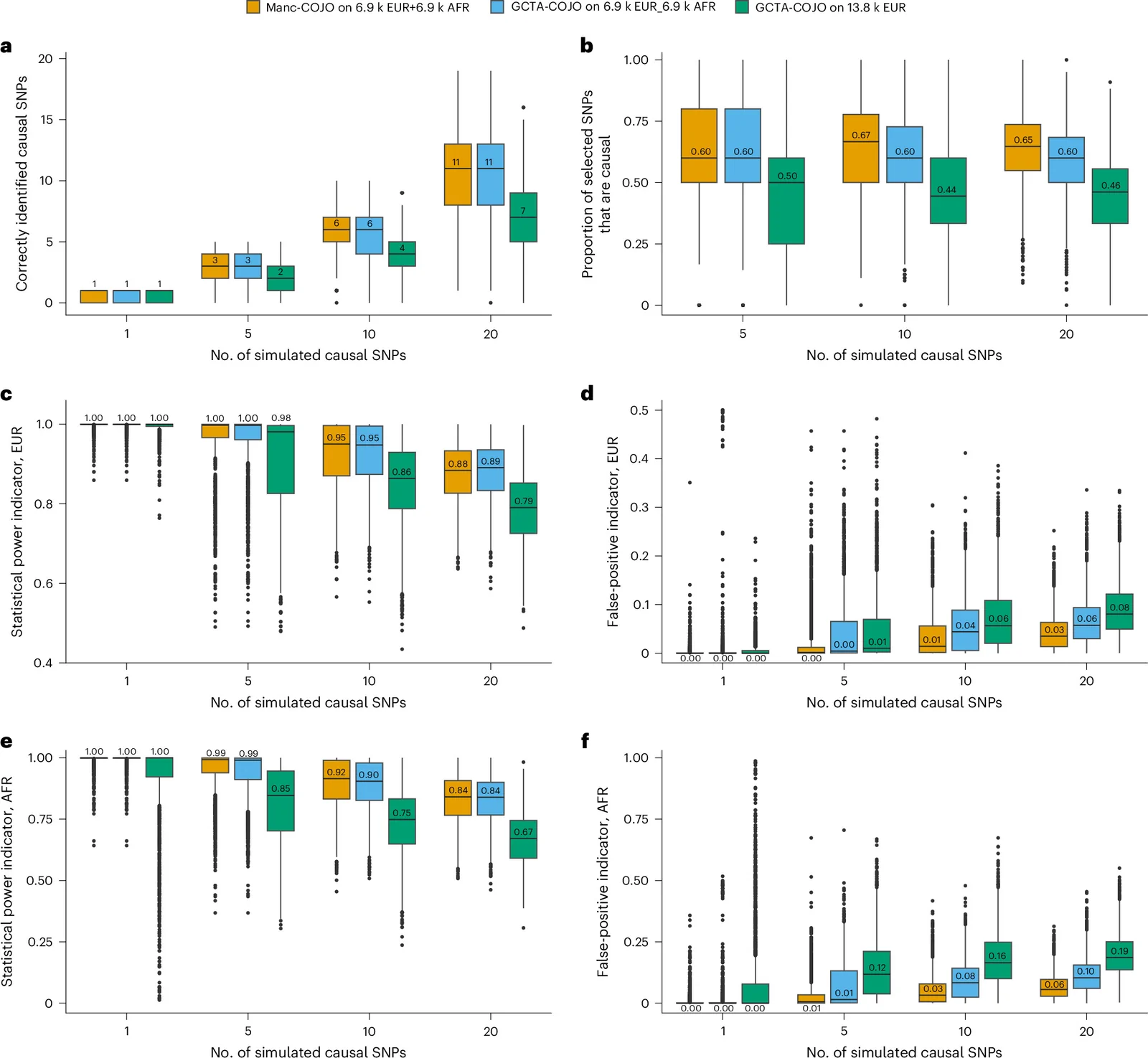
Manc-COJO identifies a similar number of causal SNPs on average, while showing superior power and fewer false-positive discoveries in simulations. Simulations were performed using genotype data from EUR and AFR cohorts (*n* = 6,901 individuals each) across 38 crossancestry, LD-independent blocks on chromosome 22. Manc-COJO jointly analyzed the EUR and AFR cohorts as separate ancestry-specific inputs. Comparisons included GCTA-COJO applied to either *n* = 13,802 EUR samples or a meta-analysis of *n* = 6,901 EUR samples and *n* = 6,901 AFR samples. In-sample LD was used for all analyses. The *x* axis is the number of simulated causal SNPs per LD-independent block. **a**, The *y* axis indicates the number of causal SNPs correctly identified by each algorithm. **b**, The *y* axis represents the proportion of identified SNPs that are causal. **c**, For each causal SNP, the COJO-SNP with the highest absolute LD (based on EUR samples) was identified and the mean LD across all causal SNPs was calculated. Higher values indicate better localization of causal variants. **d**, For each identified COJO-SNP, the causal SNP with the highest absolute LD identified (based on EUR samples) and the mean LD between them calculated. The false-positive indicator is defined as 1 − this mean LD, with higher values indicating more false-positive discoveries. **e**,**f**, Equivalent analyses shown in **c** (**e**) and **d** (**f**) using LD from AFR samples. Box plots summarize *n* = 1,900 simulation replicates and show the median, interquartile range and minimum and maximum values. Numbers above the box plots indicate the median for each group. Source data

We first compared the model output (hereafter referred to as ‘COJO-SNPs’) across three applications (note that in-sample LD is applied unless otherwise specified). In our first application, Manc-COJO was applied to GWAS summary statistics from two ancestry groups: European (EUR) and African (AFR) ancestry, each of sample size *n* = 6,901. This first application is denoted ‘Manc-COJO → 6.9 k AFR + 6.9 k EUR’ where ‘+’ indicates separate GWAS summary statistics and LD reference inputs for each ancestry. In our second application denoted ‘GCTA-COJO → 6.9 k AFR_6.9 k EUR’, GCTA-COJO was applied to the same GWAS summary statistics as in our first scenario. In the notation used for our second application, ‘_’ indicates that the GWAS summary statistics were meta-analyzed and the LD reference cohorts were combined into a single cohort. Finally, in our third application (denoted ‘GCTA-COJO → 13.8 k EUR’), GCTA-COJO was applied to EUR GWASs of the same total sample size (*n* = 6,901 × 2; Table 1). COJO-SNPs were compared to the simulation ground truth (hereafter referred to as ‘causal SNPs’) using various criteria across 7,600 simulated scenarios in 38 LD-independent blocks (Methods and Supplementary Method 2) with results summarized in Fig. 2. Trends within individual LD blocks are generally consistent with the results pooled across blocks (Supplementary Figs. 1 and 2).

**Table 1:** Summary of implemented methods

Analysis notation	Analysis description	GWAS data	LD reference
Manc-COJO → AFR data + EUR data (when not using in-sample LD) with AFR LD + EUR LD	Manc-COJO on multiancestry data	Multiancestry GWAS; separate ancestry GWASs are needed	Separate LD reference for each ancestry
GCTA-COJO → EUR data (when not using in-sample LD) with EUR LD	GCTA-COJO on single ancestry data with EUR-only LD reference	Single-ancestry EUR GWASs	EUR LD reference
GCTA-COJO → AFR data_EUR data (when not using in-sample LD) with AFR LD_EUR LD	GCTA-COJO on multiancestry data with multiancestry LD reference	Multiancestry GWAS meta-analysis (with known proportions of the different ancestries)	Single LD reference constructed from genotype data with known proportions of each ancestry
GCTA-COJO → AFR data_EUR data with EUR LD	GCTA-COJO on multiancestry data with EUR-only LD reference	Multiancestry GWAS meta-analysis	EUR LD reference

Throughout the paper, we adopt standardized terminology of the form: method → target data with LD reference (when not using in-sample LD), indicating that methods are applied to GWAS summary statistics from the target data, with the LD reference explicitly stated where in-sample LD is not used.

Under idealized simulation conditions (that is, well-powered GWASs in both ancestries, all causal SNPs genotyped, true SNP effects equal in both ancestry groups and LD structure derived from the GWAS cohort), analyses incorporating multiancestry data (that is, Manc-COJO → 6.9 k AFR + 6.9 k EUR and GCTA-COJO → 6.9 k AFR_6.9 k EUR) identify a greater number of causal SNPs (Fig. 2a,b), exhibit superior statistical power (Fig. 2c,e) and generate fewer false-positive discoveries (Fig. 2d,f) compared to single-ancestry analyses with an equivalent total sample size (that is, GCTA-COJO → 13.8 k EUR). Contrasting the two multiancestry approaches, the median numbers of causal SNPs tagged by Manc-COJO → 6.9 k AFR + 6.9 k EUR versus GCTA-COJO → 6.9 k AFR_6.9 k EUR are similar, but Manc-COJO produces fewer false-positive discoveries (Fig. 2d,f), which becomes more evident when the simulated trait is highly polygenic (that is, 10 or 20 causal SNPs within a block).

**Extended Data Fig. 1:**
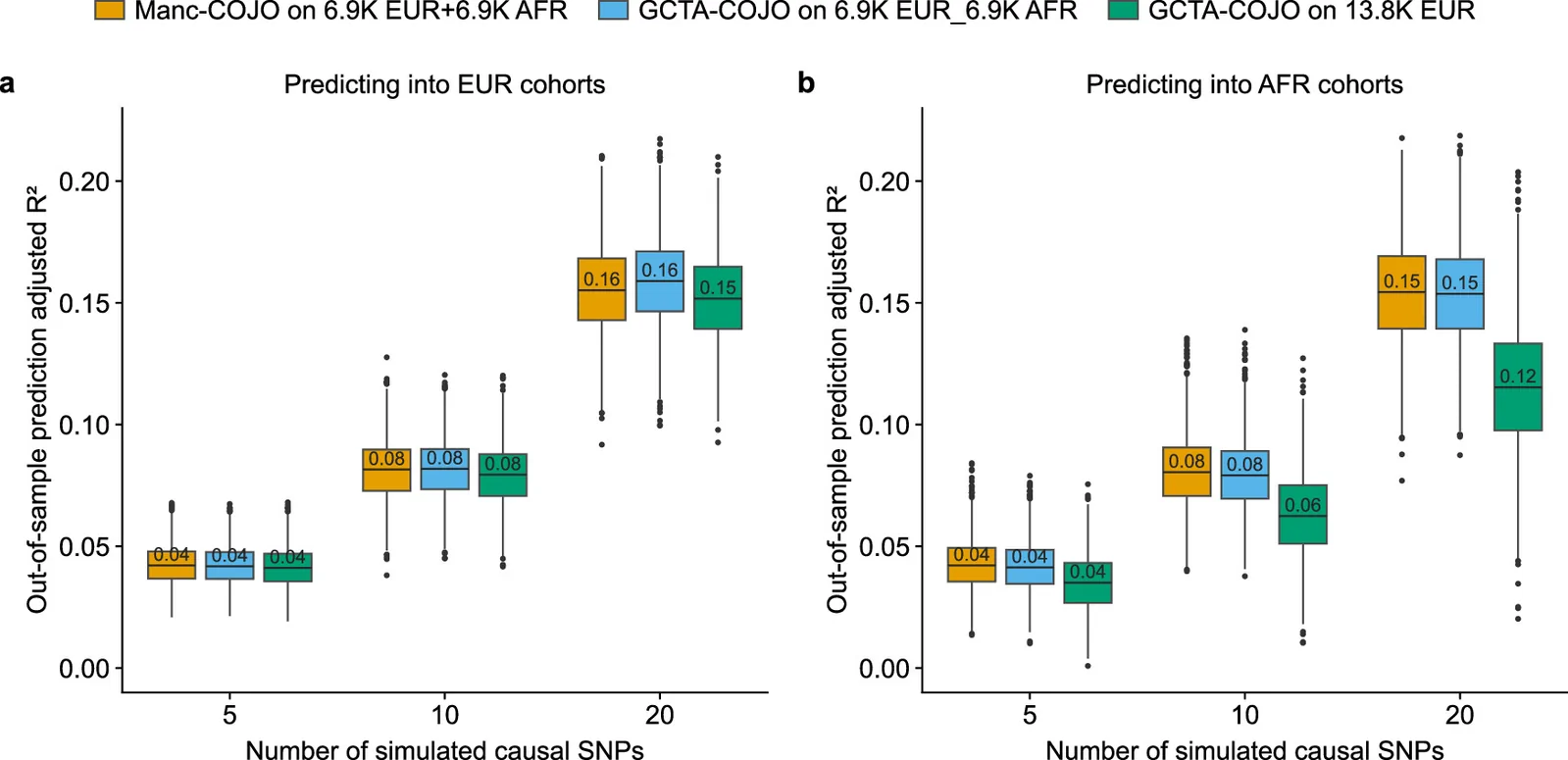
Application of GCTA-COJO to single-ancestry simulation yields a model with good out-of-sample prediction within the same ancestry but relatively low crossancestry portability in simulations. Summary of results from 5,700 simulation scenarios, illustrating the out-of-sample prediction performance of different COJO methods. SNP effect sizes were generated under the simulation settings presented in Fig. 2 and used to predict outcomes in an independent European ancestry (EUR) cohort (*N* = 13,800) and an African ancestry (AFR) cohort (*N* = 2,000). The x-axis is the number of simulated causal SNPs per independent LD block. (**a**) The y-axis indicates the adjusted *R*^*2*^ when predicting into the EUR cohort. (**b**) The y-axis indicates the adjusted *R*^*2*^ when predicting into the AFR cohort. Each box plot summarizes 1,900 simulation replicates (38 LD-independent blocks × 50 within-block replicates) and shows the minimum, maximum, mean, and interquartile range, with the numbers indicating the mean. Source data

Out-of-sample prediction using the SNPs selected by a COJO model is an objective measure to compare validity of the SNPs selected by the different models. Despite GCTA-COJO → 13.8 k EUR resulting in a model with fewer true causal SNPs and more false-positive discoveries than Manc-COJO → 6.9 k AFR + 6.9 k EUR (and GCTA-COJO → 6.9 k AFR_6.9 k EUR) from GWASs of the same total sample size, its out-of-sample prediction performance is comparable to that of Manc-COJO when predicting into an independent cohort of the same ancestry (Extended Data Fig. 1). Hence, GCTA-COJO → 13.8 k EUR SNPs have good out-of-sample prediction accuracy within an independent sample sharing a similar LD structure to the GWAS samples. However, it displays relatively low crossancestry portability, because the single-ancestry COJO-SNPs tag true causal SNPs less accurately than a multiancestry GWAS of the same total sample size, which exploits differences in LD correlation between SNPs across different ancestries^24^.

**Extended Data Fig. 2:**
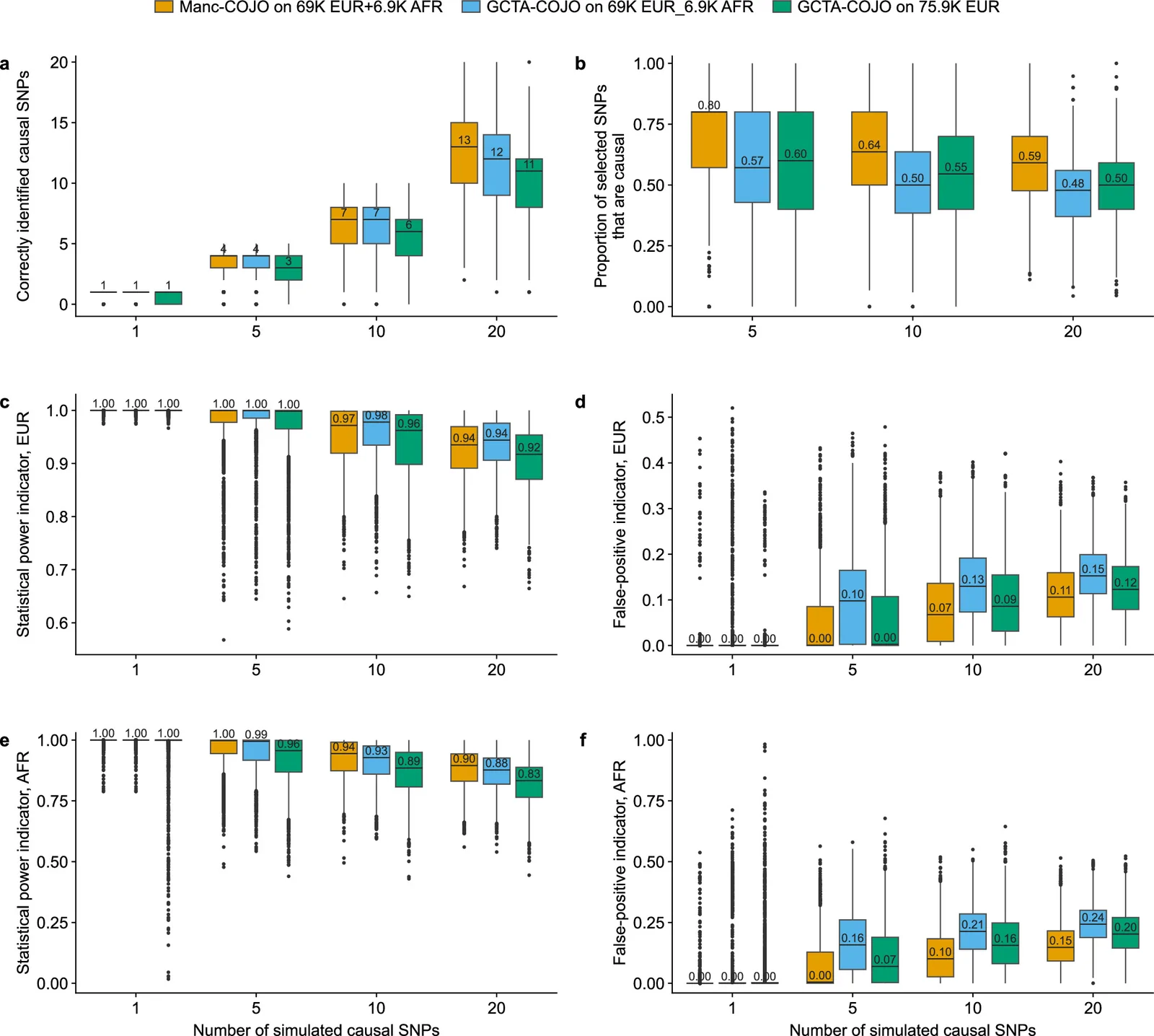
Manc-COJO demonstrates superior performance in simulations with uneven sample sizes across ancestries. Summary of results from 7,600 simulation scenarios. We apply our algorithm to a simulated European ancestry (EUR) cohort (*N* = 69,010) and a simulated African ancestry (AFR) cohort (*N* = 6,901), comparing these results with GCTA-COJO applied to either 75,911 EUR samples or a meta-analysis of 69,010 EUR samples and 6,901 AFR samples. In-sample LD was used in all scenarios. The x-axis is the number of simulated causal SNPs. (**a**) The y-axis indicates the number of causal SNPs correctly identified by each algorithm. (**b**) The y-axis represents the proportion of identified SNPs that are causal. (**c**) For each causal SNP, the COJO-SNP with the highest absolute LD (based on EUR samples) was identified, and the mean LD across all causal SNPs was calculated; higher values indicate better localization of causal variants. (**d**) For each identified COJO-SNP, the causal SNP with the highest absolute LD was identified, and the mean LD (EUR) between them was calculated. The false-positive indicator is defined as one minus this mean LD, with higher values indicating more false-positive discoveries. (**e**,**f**) Equivalent analyses shown in **c** (**e**) and **d** (**f**) using LD from AFR samples. Numbers in each plot represent the median value for each group. Each box plot summarizes 1,900 simulation replicates (38 LD-independent blocks × 50 within-block replicates) and shows the minimum, maximum, median, and interquartile range, with the numbers indicating the median. This figure illustrates that Manc-COJO, on average, identifies a greater number of causal SNPs, exhibits superior power, and produces fewer false-positive discoveries when sample sizes across ancestries are unequal. Source data

In practice, model assumptions are often violated. To investigate the impact of such violations, we conducted a series of simulations in which one assumption was altered at a time. To mimic the scarcity of multiancestry data, we simulated a scenario in which the EUR cohort sample size was increased 10-fold to 69,010. Compared with GCTA-COJO-based methods, Manc-COJO → 6.9 k AFR + 69 k EUR, on average, identifies a greater number of causal SNPs, exhibits superior power and produces fewer false-positive discoveries in simulations (Extended Data Fig. 2). This demonstrates that, even when sample sizes are heavily skewed toward EUR ancestry, incorporating AFR ancestry data still enhances the ability to detect independent associations with fewer false-positive discoveries. Notably, the application of GCTA-COJO to multiancestry data, GCTA-COJO → 6.9 k AFR_69 k EUR—even when using an LD matrix constructed from the same individuals included in the analysis (that is, in-sample mixed-ancestry LD)—results in a higher number of false-positive discoveries. Specifically, the proportion of SNPs selected by the GCTA-COJO → 6.9 k AFR_69 k EUR algorithm that are truly causal is 0.57, 0.50 and 0.48 for regions with 5, 10 and 20 causal SNPs, respectively. These values are not only lower than those obtained with Manc-COJO (0.80, 0.64 and 0.59 for the same scenarios), but also lower than those from GCTA-COJO → 75.9 k EUR (0.60, 0.55 and 0.50 for the same scenarios and total sample size).

**Extended Data Fig. 3:**
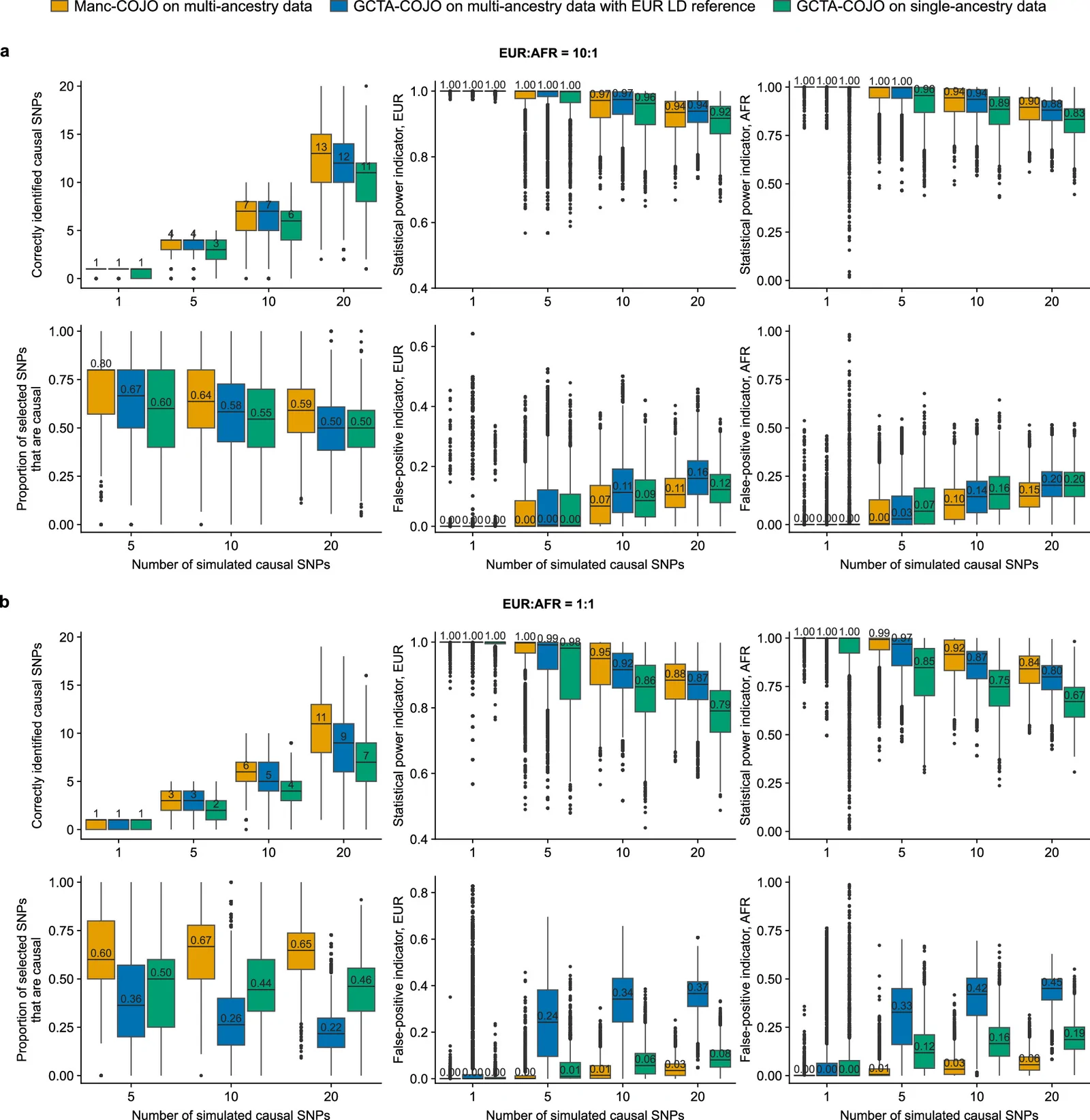
Effectiveness of European-only LD reference panels depends on ancestry composition of the study sample in simulations. Performance of GCTA-COJO applied to multiancestry GWAS summary statistics using a EUR-only LD reference panel, under two different sample size scenarios: a EUR:AFR ratio of 10:1 (**a**) and 1:1 (**b**). Each box plot summarizes 1,900 simulation replicates (38 LD-independent blocks × 50 within-block replicates) and shows the minimum, maximum, median, and interquartile range, with the numbers indicating the median. Model performance is evaluated as in Fig. 2. When EUR dominate the sample (10:1 ratio), using a EUR-only LD reference performs reasonably well, achieving higher power in both ancestries and lower false-positive rates in AFR samples, compared to GCTA-COJO applied to European-only summary statistics. However, when the ancestry balance is equal (1:1 ratio), the use of a EUR-only LD reference becomes ineffective, leading to marked inflation in false-positive discoveries, particularly in AFR samples. Source data

When sample sizes are heavily biased toward EUR populations, some studies have applied a EUR-only LD reference even when the summary statistics were derived from multiancestry GWAS meta-analyses^5,7^. In simulations, when EUR dominates the sample (10:1 ratio), such an approach (that is, GCTA-COJO → 6.9 k AFR_69 k EUR with 75.9 k EUR LD) performs reasonably well, achieving higher power in both ancestries and making fewer false-positive discoveries in AFR, compared to GCTA-COJO → 75.9 k EUR (Manc-COJO → 6.9 k AFR + 69 k EUR is still superior). When the ancestry balance is equal (1:1 ratio), the use of a EUR LD reference becomes ineffective, leading to marked inflation in false-positive discoveries (Extended Data Fig. 3).

Other sensitivity analyses are reported in Supplementary Note 1 and Supplementary Figs. 3–5. These included an analysis focusing on HapMap3 SNPs to mimic when causal SNPs are not genotyped, simulations where effect sizes differ between ancestries and simulations where LD matrices were not calculated from the GWAS samples. As expected, the performance of all models declined under nonidealized conditions. Nevertheless, Manc-COJO consistently demonstrated greater robustness compared to the GCTA-COJO-based methods.

### Real-data analysis

Given that causal SNPs and their effects are typically unknown for most traits, we use the out-of-sample prediction accuracy (*R*^2^) from the COJO-SNPs and model size as the primary performance criteria for comparing GCTA-COJO and Manc-COJO in real-data analyses. We analyzed summary statistics from a GWAS meta-analysis of lipid traits (high-density lipoprotein-cholesterol (HDL-C), low-density lipoprotein-cholesterol (LDL-C), total cholesterol (TC) and log(triglyceride) (log(TG))) including approximately 1 million samples (Supplementary Table 1).

**Fig. 3:**
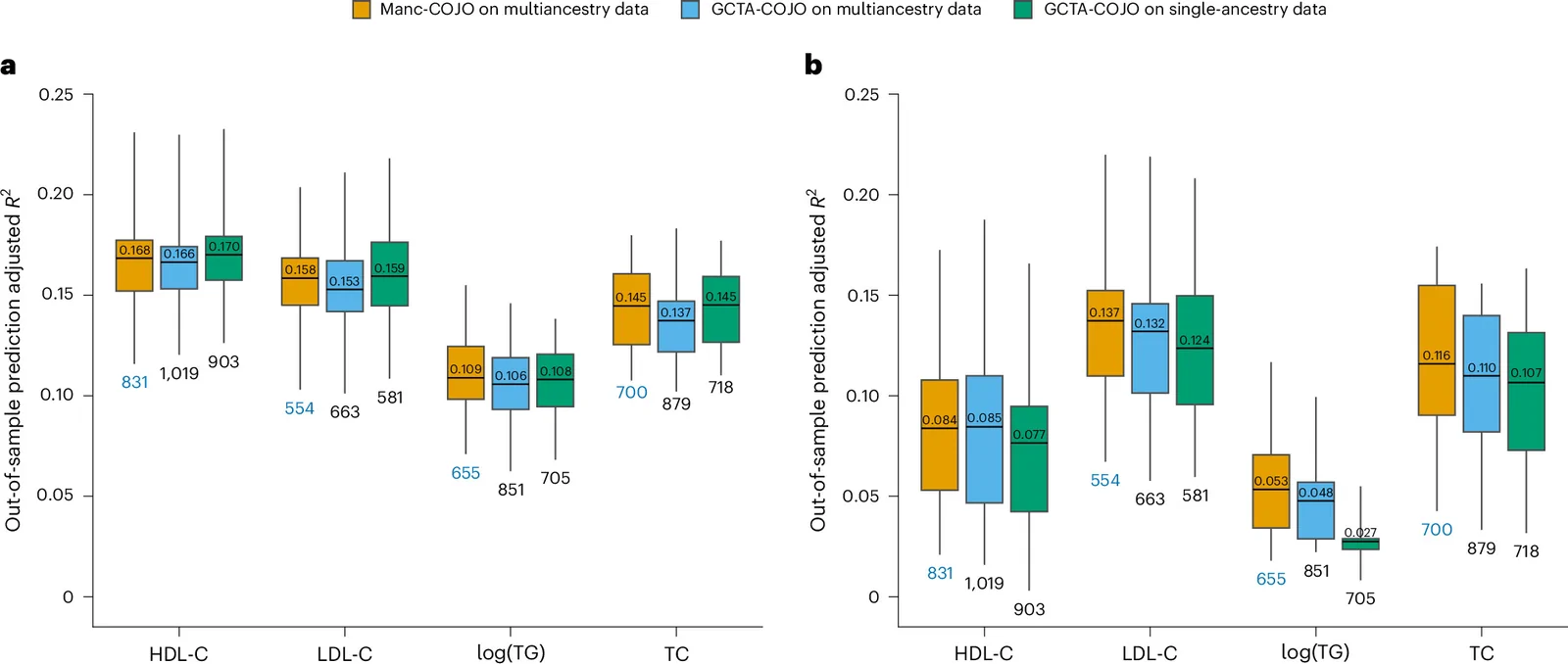
Manc-COJO selected fewer COJO-SNPs with superior transancestry portability compared to the single-ancestry GCTA-COJO model. **a**,**b**, Out-of-sample prediction accuracy evaluated for Manc-COJO and GCTA-COJO applied to multiancestry data and GCTA-COJO applied to single-ancestry data for HDL-C, LDL-C, log(transformed) TG (log(TG)) and TC in independent EUR (**a**) and AFR (**b**) cohorts. Numbers below each box plot indicate the number of independently associated SNPs identified by each method; the model with the fewest SNPs is highlighted in blue for each trait. (1) Manc-COJO jointly analyzed EUR-specific (Global Lipids Genetics Consortium (GLGC), *n* ≈ 0.9 million) and AFR-specific (GLGC, *n* ≈ 90,000) GWAS summary statistics as separate ancestry-specific inputs. The LD reference panels comprised *n* = 69,010 unrelated EUR and *n* = 6,901 unrelated AFR individuals in the UKB. (2) GCTA-COJO on multiancestry data was applied to a meta-analysis of the EUR-specific and AFR-specific GWAS summary statistics using a combined EUR–AFR LD reference panel of the same individuals. (3) GCTA-COJO on single-ancestry data was applied to EUR-specific GWAS summary statistics meta-analyzed with an additional independent EUR UKB GWAS (*n* ≈ 90,000), using an LD reference panel comprising *n* = 75,911 unrelated EUR individuals. All GWAS summary statistics excluded UKB participants unless otherwise specified. To ensure fair comparisons, all methods used harmonized GWAS summary statistics with identical SNP sets, approximately matched sample sizes and LD reference panels of comparable size. Box plots summarize the distribution of out-of-sample prediction *R*^2^ values across all evaluation groups (50 EUR and 20 AFR cohorts) and show the mean, interquartile range and minimum and maximum values. Source data

We first assessed the prediction accuracy of single-ancestry GCTA-COJO-derived and Manc-COJO-derived predictors in an independent sample of 47,066 EUR individuals. The prediction *R*^2^ (and s.d.) of the Manc-COJO → 0.09 M AFR + 0.9 M EUR with 6.9 k AFR + 69 k EUR LD model was 0.168 (0.023), 0.158 (0.021), 0.109 (0.018) and 0.145 (0.020) for HDL-C, LDL-C, log(TG) and TC, respectively. The *R*^2^ value of the GCTA-COJO → 0.99 M EUR with 75.9 k EUR LD was similar: 0.170 (0.022), 0.159 (0.023), 0.108 (0.017) and 0.145 (0.020) for the same traits. When applied to an independent cohort of 3,000 AFR individuals, the Manc-COJO model consistently yielded higher *R*^2^ values (although not statistically different given the sample size available) than GCTA-COJO: 0.084 (0.040) versus 0.077 (0.044) for HDL-C, 0.137 (0.046) versus 0.124 (0.040) for LDL-C, 0.0534 (0.028) versus 0.0274 (0.013) for log(TG) and 0.116 (0.044) versus 0.107 (0.040) for TC. Notably, the number of independently associated SNPs identified by Manc-COJO was much lower than that of the GCTA-COJO model: 831 versus 903 for HDL-C, 554 versus 581 for LDL-C, 655 versus 705 for log(TG) and 700 versus 718 for TC (Fig. 3). In summary, Manc-COJO achieved comparable predictive accuracy in the EUR sample and superior performance in the AFR sample, while selecting fewer SNPs, suggesting a lower rate of false-positive inclusions relative to the GCTA-COJO model (consistent with simulation results). We replicated the out-of-sample prediction analysis in the All of Us cohort and found consistent trends with those observed in the UK Biobank (UKB; Table 2).

**Table 2:** Replication of out-of-sample predictions in the All of Us cohort

Method	HDL-C	LDL-C	log(TG)	TC
Manc-COJO → 0.09 M AFR + 0.9 M EUR with 6.9 k AFR + 69 k EUR LD	809 0.119 (0.021) 0.0830 (0.022)	533 0.0914 (0.019) 0.100 (0.023)	631 0.0876 (0.019) 0.0347 (0.016)	693 0.0808 (0.018) 0.0843 (0.018)
GCTA-COJO → 0.99 M EUR with 75.9 k EUR LD	884 0.122 (0.021) 0.0698 (0.019)	565 0.0902 (0.019) 0.0866 (0.018)	677 0.0911 (0.019) 0.0297 (0.016)	700 0.0815 (0.019) 0.0665 (0.017)
GCTA-COJO → 0.09 M AFR_0.9 M EUR with 6.9 k AFR_69 k EUR LD	1022 0.117 (0.021) 0.0782 (0.020)	638 0.0852 (0.018) 0.0949 (0.024)	829 0.0871 (0.020) 0.0337 (0.015)	880 0.0801 (0.019) 0.0755 (0.016)
GCTA-COJO → 0.09 M AFR_0.9 M EUR with 75.9 k EUR LD	845 0.118 (0.021) 0.0818 (0.022)	593 0.0903 (0.019) 0.0955 (0.020)	650 0.0868 (0.020) 0.0382 (0.017)	730 0.0813 (0.018) 0.0800 (0.015)

Within each cell, the first row reports the model size, the second row reports the mean out-of-sample prediction accuracy (and s.d.) among unrelated EUR ancestry participants and the third row reports the corresponding accuracy (and s.d.) among unrelated AFR ancestry participants.

In previous attempts to adapt GCTA-COJO for multiancestry GWASs, an ad-hoc approach employed LD matrices estimated from samples with ancestry composition matching that of the GWAS sample^9,10^. In real-data analyses, the resulting models were substantially larger than those produced by Manc-COJO: the number of SNPs selected by the GCTA-COJO → 0.09 M AFR_0.9 M EUR with 6.9 k AFR_69 k EUR LD model was 1,019 for HDL-C, 663 for LDL-C, 851 for log(TG) and 879 for TC—approximately 25% more SNPs than identified by Manc-COJO models (Fig. 3). Despite this increased model complexity, predictive performance was marginally lower than that of Manc-COJO across all traits and scenarios. These results indicate that the GCTA-COJO → 0.09 M AFR_0.9 M EUR with 6.9 k AFR_69 k EUR LD model is more prone to false-positive SNP inclusion than the more parsimonious Manc-COJO approach, in agreement with the simulation findings.

**Extended Data Fig. 4:**
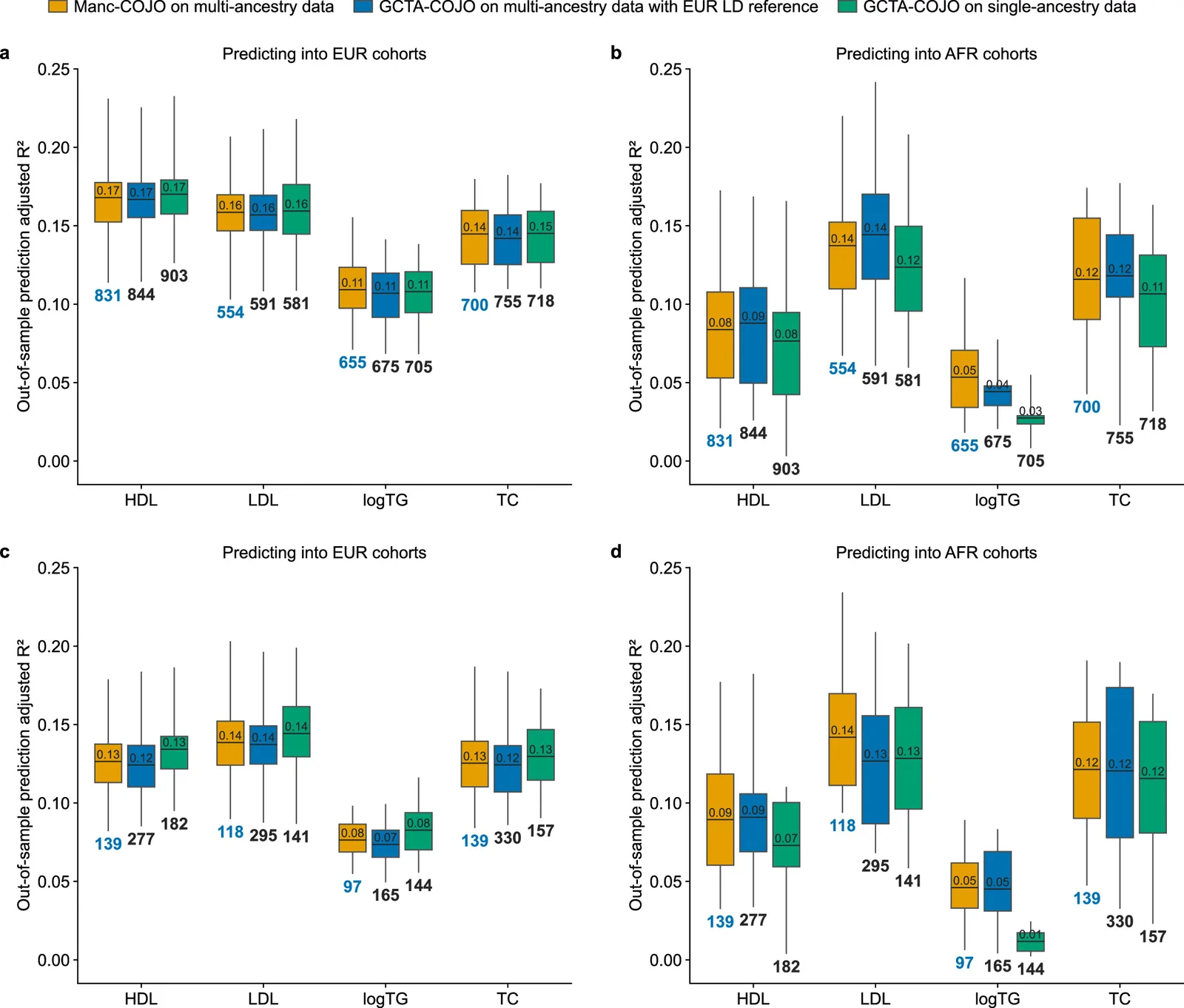
Applying GCTA-COJO to meta-analyses of multiancestry GWAS using European-only LD reference panels is only effective when European populations dominate the sample. Published studies have applied GCTA-COJO to multiancestry GWAS meta-analyses using LD panels only calculated from European ancestry (EUR) genomes, particularly when EUR individuals comprised the majority of the sample. Here, we apply GCTA-COJO using a EUR-only LD reference under two scenarios: a EUR:AFR sample size ratio of 10:1 (**a**, **b**) and 1:1 (**c**, **d**), predicting into both an independent European cohort (**a**, **c**) and an AFR cohort (**b**, **d**). The results mirror our simulation findings. Each box plot shows the distribution of the out-of-sample prediction *R*^2^ values for all groups (50 in EUR and 20 in AFR) and displays the minimum, maximum, mean, and interquartile range. When Europeans dominate the sample (10:1), the approach performs reasonably well. However, when the ancestry balance shifts to 1:1, the strategy becomes ineffective. The number of SNPs selected by GCTA-COJO increases markedly, indicating a higher rate of false-positive inclusions and reduced model parsimony. Source data

In some studies^5,7^ GCTA-COJO has been applied to meta-analyses of multiancestry GWASs using EUR-only LD reference panels, particularly in studies where EUR populations dominate the sample sizes. The results aligned with observations from our simulations. When the EUR-to-AFR sample size ratio was 10:1, this approach was reasonably effective—GCTA-COJO → 0.09 M AFR_0.9 M EUR with 75.9 k EUR LD produced models with comparable out-of-sample prediction accuracy in both EUR and AFR cohorts and the model sizes remained similar. However, as the ancestry balance shifted to a 1:1 ratio, this approach was no longer viable (evaluated through downsampling; Supplementary Note 2). The number of SNPs selected by the GCTA-COJO → 0.09 M AFR_0.09 M EUR with 13.8 k EUR LD model increased substantially, reaching approximately twice the size of the corresponding Manc-COJO → 0.09 M AFR + 0.09 M EUR with 6.9 k AFR + 6.9 k EUR LD models (Extended Data Fig. 4). Moreover, for all traits except TC, these model sizes also exceeded those of GCTA-COJO → 0.09 M AFR_0.09 M EUR with 6.9 k AFR_6.9 k EUR LD models (which were constructed with ancestry-proportion-matched LD reference panels), suggesting a higher rate of false-positive SNP inclusion.

### Manc-COJO:FIX

**Fig. 4:**
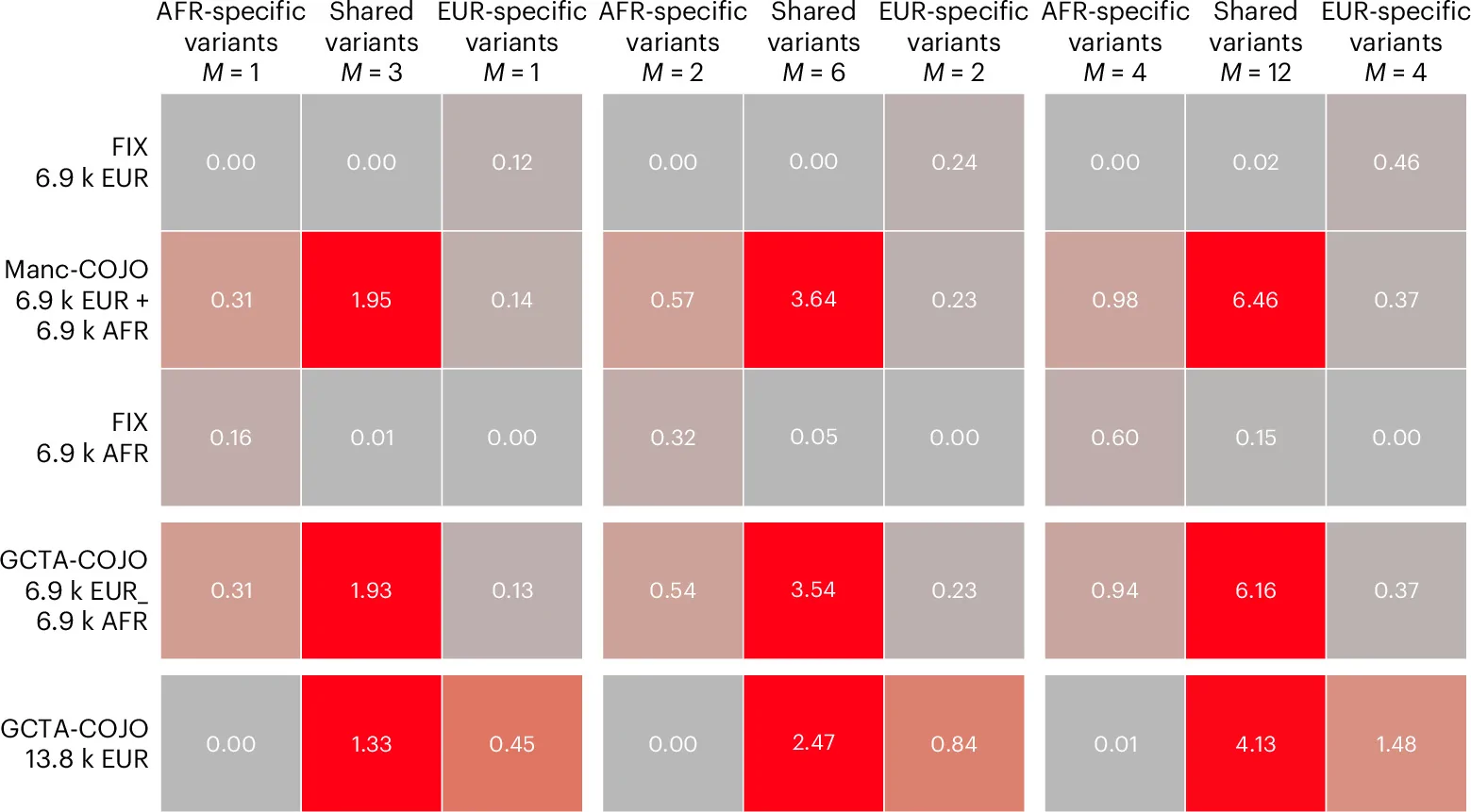
Manc-COJO identifies a greater number of shared causal SNPs in scenarios where ancestry-specific, trait-associated variants exist, whereas Manc-COJO:FIX recovers some ancestry-specific associations. Simulations performed across 38 crossancestry LD-independent blocks on chromosome 22 with varying numbers of causal variants (5, 10 or 20), including both shared and ancestry-specific causal SNPs. The columns indicate the number (*M*) and ancestry composition of simulated causal variants and the rows correspond to the analysis method. The numbers in each box represent the mean number of causal SNPs correctly identified across all simulation replicates, with the color scale indicating performance (red, higher; gray, lower). Manc-COJO was applied jointly to simulated EUR and AFR cohorts and Manc-COJO:FIX denotes a secondary conditional analysis with variants identified by Manc-COJO fixed in the model. GCTA-COJO was applied to either a EUR-only cohort or a multiancestry meta-analysis using in-sample LD. Source data

All simulations and real-data analyses described above assumed shared causal SNPs across ancestries. We developed a complementary approach, termed Manc-COJO:FIX, which allows users to retain specified variants in the COJO model by preventing their removal during the variable selection process. SNPs can be fixed in both multiancestry and single-ancestry settings (Supplementary Note 3). Fixing SNPs identified in the Manc-COJO analysis and subsequently running Manc-COJO:FIX within each ancestry-specific dataset allowed the identification of variants with potential ancestry-specific effects. To evaluate the performance of this pipeline, we designed a series of simulated scenarios in which 60% of the simulated SNPs in a block were common across ancestries, whereas 20% were specific to each of the two ancestries (Methods). Both multiancestry algorithms (Manc-COJO → 6.9 k AFR + 6.9 k EUR and GCTA-COJO → 6.9 k AFR_6.9 k EUR) detected more shared causal variants than single-ancestry GCTA-COJO of the same total GWAS sample size (that is, GCTA-COJO → 13.8 k EUR), with Manc-COJO outperforming GCTA-COJO → 6.9 k AFR_6.9 k EUR. Applying Manc-COJO:FIX to the Manc-COJO output recovers 10–20% additional ancestry-specific, simulated causal variants (Fig. 4). In real-data analysis, we applied Manc-COJO:FIX (using ~6.5 M SNPs; fixing SNPs identified in Manc-COJO → 0.09 M AFR + 0.9 M EUR with 6.9 k AFR + 69 k EUR LD) within the ~1 M EUR and ~0.09 M AFR cohorts separately to test for ancestry-specific associations. No new SNPs were identified in the AFR cohort. In the EUR cohort, compared with the Manc-COJO model, the increase in prediction *R*^2^ (and corresponding increase in model size) was 0.0007 (8), 0.0003 (4), 0.0005 (17) and 0.0002 (2) for HDL-C, LDL-C, log(TG) and TC, respectively. Overall, these results suggest that most of the predictive performance can be attributed to models that assume shared common causal SNPs across ancestries (Supplementary Note 3).

### Software improvement compared to GCTA-COJO

**Extended Data Fig. 5:**
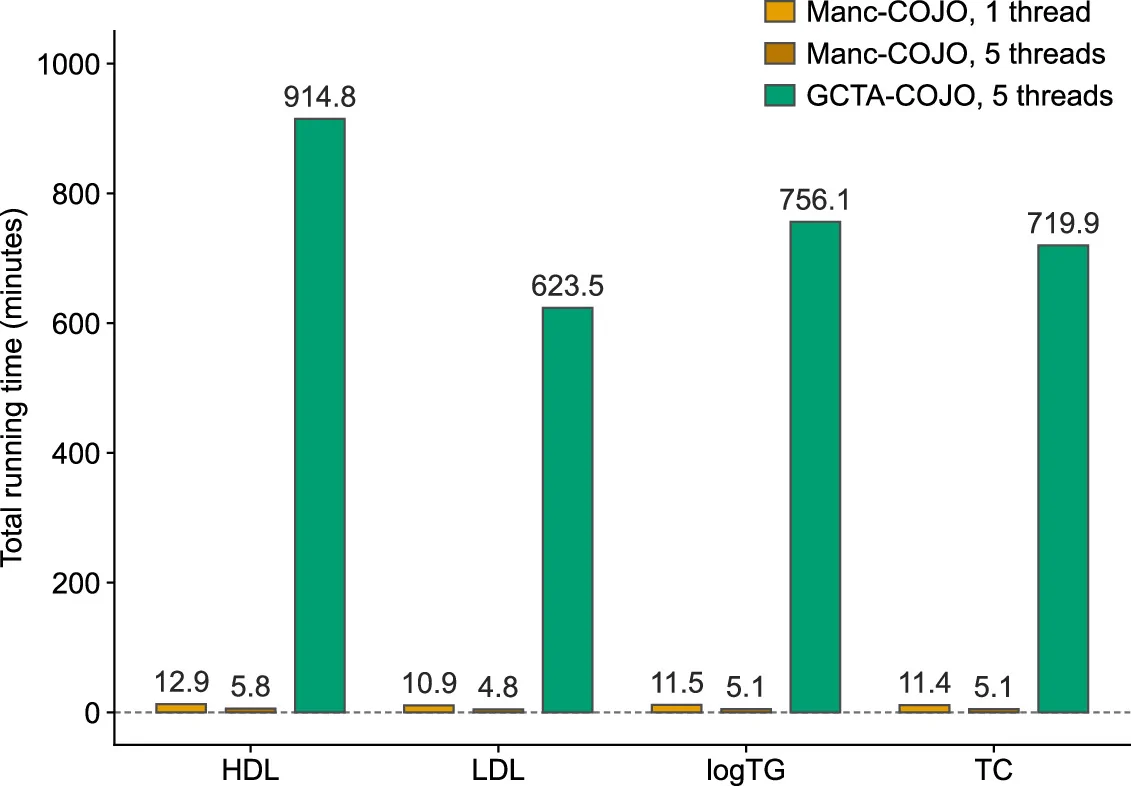
Improved computational efficiency of our software for running GCTA-COJO compared with the implementation in GCTA. Although our software is primarily designed for multiancestry analysis, it also supports single-ancestry COJO analyses as in GCTA. The x-axis shows the traits, and the y-axis shows the total running time across all 22 chromosomes for single-ancestry analysis, using *N* = 75,911 unrelated UKB EUR samples as the LD reference. Even using a single thread, the average per-chromosome runtime is below one min—orders of magnitude faster than the GCTA-COJO algorithm currently implemented in GCTA. Source data

Our Manc-COJO software implemented the same single-ancestry COJO algorithm as GCTA, alongside several algorithmic improvements to enhance its robustness (Supplementary Note 4) and computational efficiency. We benchmarked the total running time across all 22 chromosomes for single-ancestry analysis in ~1 M EUR individuals (~6.5 M SNPs; as described in the real-data analysis), using ~75,000 unrelated UKB EUR samples as the LD reference. With 5 threads, our software required only 5.80 min, 4.83 min, 5.12 min and 5.08 min for HDL-C, LDL-C, log(TG), and TC, respectively (that is, on average <30 s per chromosome when run in parallel). In contrast, GCTA required 915 min, 623 min, 756 min and 720 min under the same central processing unit and memory settings, corresponding to speedups of 158×, 129×, 147× and 142×, respectively. The design principles underlying these improvements may be relevant to other GWAS summary statistics software (Extended Data Fig. 5 and Supplementary Note 4).

### Insights into Manc-COJO and GCTA-COJO outputs

**Fig. 5:**
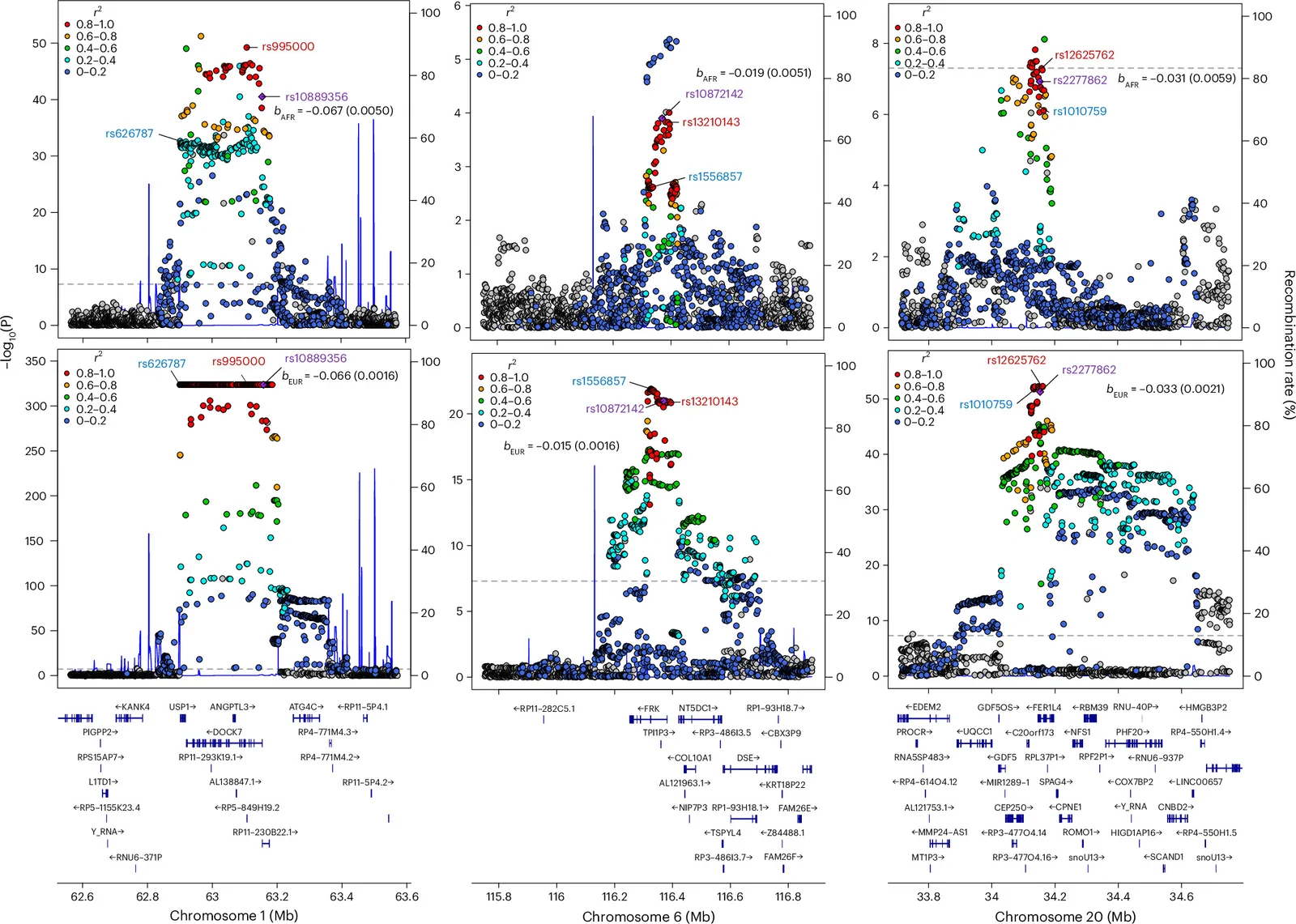
LocusZoom plots comparing variants selected by Manc-COJO and GCTA-COJO at loci containing experimentally supported functional variants. Regional association signals, LD and recombination rates shown for three SNP–gene pairs and their relevant lipid traits: rs10889356–*DOCK7* and *ANGPTL3* for TC (left), rs10872142–*FRK* for LDL-C (middle) and rs2277862–*CPNE1* for TC (right). Top: LD structure and GWAS summary statistics from AFR data (as in Fig. 3); bottom: the corresponding EUR results. Experimentally supported functional variants are labeled in purple, variants selected by Manc-COJO are highlighted in red and variants selected by GCTA-COJO are highlighted in blue. Point colors indicate squared LD correlations (*r*^2^) with the corresponding functional variant and numbers adjacent to the functional variants indicate their marginal GWAS effect estimates. The EUR data show more extensive LD, particularly in the lower-left panel, where several variants are in near-complete LD. The contrasting LD patterns across ancestries illustrate how incorporating AFR data can help distinguish highly correlated variants. At these loci, Manc-COJO selected variants that more closely tagged the experimentally supported functional variants in AFR data than those selected by GCTA-COJO. Marginal effect estimates for the functional variants were broadly similar across ancestries (two-sided *z*-tests comparing effect estimates between AFR and EUR, Bonferroni’s adjusted *P* > 0.05 for all three variants). Source data

To assess differences between Manc-COJO (Manc-COJO → 0.09 M AFR + 0.9 M EUR with 6.9 k AFR + 69 k EUR LD) and GCTA-COJO (GCTA-COJO → 0.99 M EUR with 75.9 k EUR LD), we examined their outputs at loci containing experimentally supported functional variants. Previous studies used clustered regularly interspaced short palindromic repeat-based and other functional assays to support the following SNP–gene pairs: rs12740374–*SORT1* (LDL-C)^25^, rs2277862–*CPNE1* (HDL-C and TC), rs10889356–*DOCK7/ANGPTL3* (TG and TC) and rs10872142–*FRK* (LDL-C)^26^; the lipid traits shown in parentheses indicate those linked to each SNP–gene pair on the basis of GWAS and/or mouse-model evidence. For LDL-C, both methods included rs12740374. For the other three variants, both selected high-LD proxies in EUR samples. In each example, however, Manc-COJO selected an SNP with a higher absolute LD correlation with the functional variant in AFR samples. The AFR correlations for Manc-COJO versus GCTA-COJO were 0.942 versus 0.918 for rs2277862 in TC, 0.960 versus 0.476 for rs10889356 in TC and 1.000 versus 0.899 for rs10872142 in LDL-C. Thus, at these loci, incorporating AFR GWAS and LD data improved the tagging of the known functional variants. Out-of-sample prediction point estimates showed a similar pattern, but the small validation samples provided insufficient power to establish statistically significant differences (Fig. 5 and Supplementary Note 5). We also observed broadly similar marginal effect estimates for these functional variants across AFR and EUR GWASs (two-sided *z*-tests: rs2277862 for TC, *z* = 0.22, *P* = 0.83; rs10889356 for TC, *z* = 0.33, *P* = 0.74; and rs10872142 for LDL-C, *z* = 0.83, *P* = 0.41).

## Discussion

We developed a multiancestry algorithm to perform conditional and joint analysis of GWAS summary statistics. We demonstrated through extensive simulations that Manc-COJO outperformed the single-ancestry-based COJO method (that is, GCTA-COJO) applied on both single-ancestry and multiancestry data by correctly identifying, on average, more independent associated variants and reducing false-positive associations. Moreover, Manc-COJO was robust to scenarios when sample sizes were biased, causal SNPs not all genotyped, true SNP effects different across ancestries and imperfect LD references. We applied Manc-COJO to four polygenic lipid traits (which have genetic architectures that include large effect–size associations) and validated SNPs selected by Manc-COJO using out-of-sample prediction (Supplementary Note 5). A key result from our analyses is that Manc-COJO achieved comparable predictive accuracy in EUR ancestry cohorts and better portability in AFR ancestry cohorts, while selecting fewer SNPs. This indicates that the number of independent associations detected using single-ancestry data may be overestimated. Moreover, our results also show that Manc-COJO improves tagging of causal variants by leveraging differences in LD structures among ancestry groups (especially AFR ancestry) even when certain groups are only modestly represented in the GWAS (for example, 10%).

Previous efforts to adapt GCTA-COJO for identification of independent associations in multiancestry GWAS meta-analyses likely include false discoveries, although current sample sizes mean that the impact is limited. Use of LD estimated in EUR ancestry samples in multiancestry analysis performs adequately when the EUR-to-AFR sample size ratio is around 10:1, but it leads to increasing false positives as this ratio approaches 1:1. Pinpointing the exact threshold where performance deteriorates is challenging (and likely varies by chromosomal region and genetic architecture). Likewise, constructing LD reference panels that match the ancestry proportions of the GWASs can work well under idealized conditions (for example, in-sample LD, balanced ancestral representation and sufficient power) but become prone to inflated false discoveries when these assumptions are violated. One potential contributing factor to inflated false discoveries is that all COJO models assume Hardy–Weinberg equilibrium (HWE) in their calculations, which is violated in multiancestry LD reference panels (Supplementary Note 6).

Here we focused on individuals of inferred EUR and AFR ancestry as a proof of principle. Nevertheless, the Manc-COJO methodology is readily extendible and our software supports the inclusion of an arbitrary number of ancestry groups. Notably, Manc-COJO operates on variants, for which association statistics (for example, estimated effect size, s.e. and per-variant sample size) are available across all ancestry groups represented in the GWAS meta-analysis. Consequently, there is a trade-off between increasing sample size and ancestry diversity because inclusion of more datasets may reduce the number of SNPs available for analysis. Data input decisions are described in detail in Supplementary Note 7. Although this report applies meta-analysis strategies to integrate data across ancestries, the software can also be adapted to perform mega-analysis (that is, pooling individual-level genotype data) or analyses within admixed populations, which in principle may offer greater power when in-sample LD is available and sample sizes are sufficiently large^27^, but may be prone to inflated false positives when these ideal conditions are not met (Supplementary Note 6).

Although an increasing number of studies provide empirical evidence that causal SNPs are shared across ancestries with similar effect sizes^18–21^, ancestry-specific causal variants have also been reported^22,23^. To address this, we developed a complementary method, Manc-COJO:FIX, to accommodate such scenarios. In real-data applications, this algorithm identified only a small number of additional variants in the EUR cohort. Importantly, these variants should not be interpreted as definitive evidence of ancestry-specific effects for lipid traits. On the one hand, the additional variants accounted for only a negligible increase in out-of-sample prediction *R*^2^ values. On the other hand, their absence in the AFR group may simply reflect limited statistical power, causing true associations to fall below significance thresholds. Thus, at least in the near future, bioinformatics-based claims of ancestry-specific variants should be supported by direct functional validation. With this in mind, we designed Manc-COJO:FIX to allow users to retain experimentally validated causal variants in the model. This enables an iterative workflow in which bioinformatic analyses not only guide downstream experimental validation but also incorporate experimentally validated variants back into the modeling framework, potentially improving the detection of additional independent association signals (Supplementary Note 3).

**Extended Data Fig. 6:**
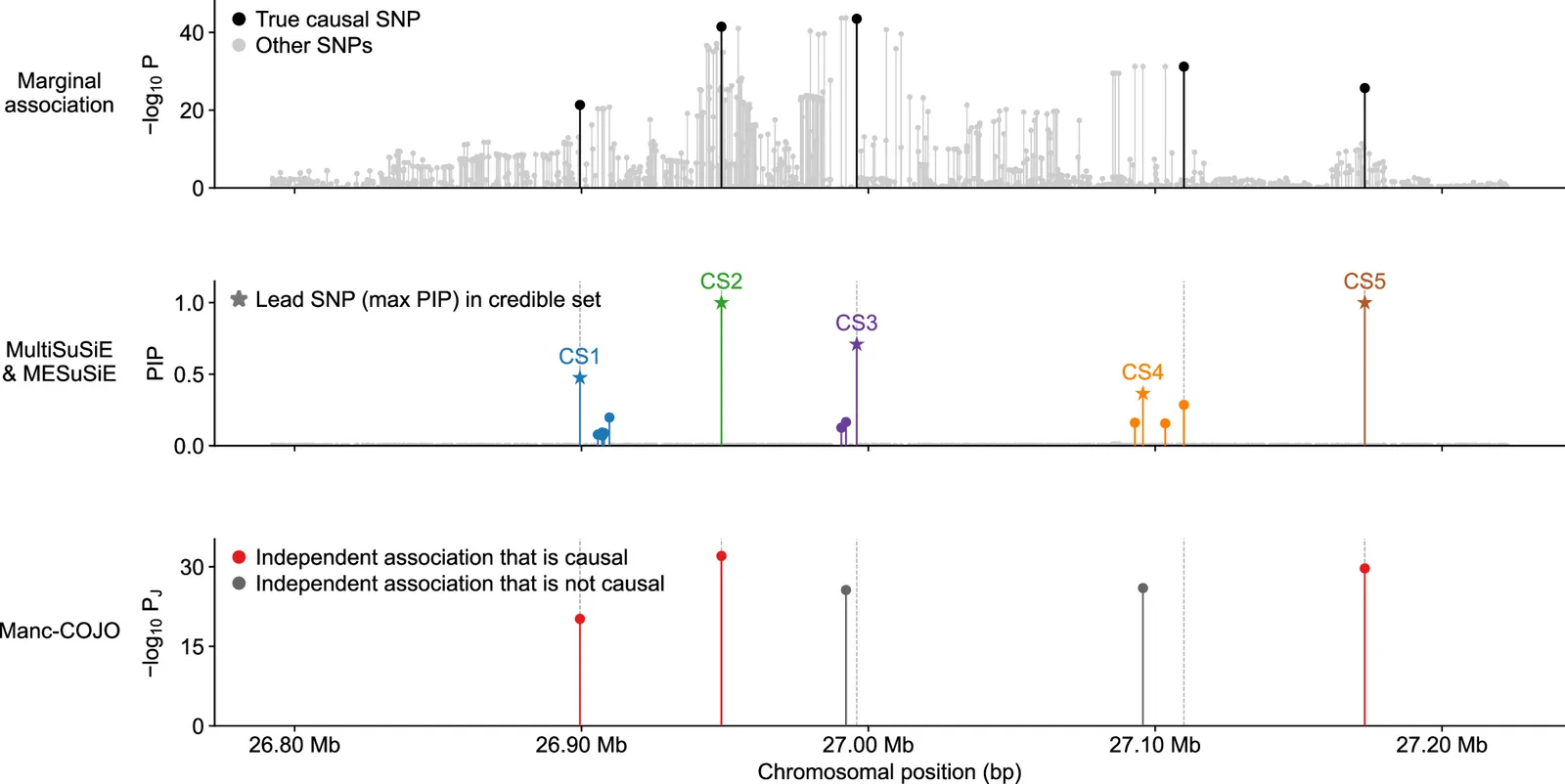
Locus visualization of fine-mapping and COJO analysis results for a representative simulation replicate. Each panel displays SNPs along a 400 kb region of chromosome 22. (Top panel) Marginal association signals for all SNPs, where y-axis is -log_10_p with p denoting the marginal GWAS *P* value from the combined multiancestry summary statistics. True causal SNPs are highlighted in black; all other SNPs are shown in gray. (Middle panel). Posterior inclusion probabilities (PIPs) estimated by MultiSuSiE and MESuSiE, which produced identical credible sets in this replicate. Each credible set (CS1–CS5) is shown in a distinct color; the star marker denotes the lead SNP (maximum PIP within the credible set) and circles denote other credible set members. y-axis represents the PIP of each SNP, reflecting the posterior probability that the SNP is causal. Dashed vertical lines indicate the positions of true causal SNPs. (Bottom panel) SNPs selected by Manc-COJO, where y-axis is -log_10_p_J_, with p_J_ denoting the joint *P* value obtained from stepwise selection. Red markers indicate true causal SNPs identified, whereas gray markers denote selected SNPs that are not causal. This difference reflects the distinct objectives of the methods. Fine-mapping approaches such as MultiSuSiE and MESuSiE model all SNPs jointly and report credible sets that capture uncertainty, often including correlated variants. In contrast, COJO performs stepwise conditional analysis to identify a parsimonious set of approximately independent association signals, where tagging a SNP in linkage disequilibrium with the causal variant is typically sufficient. Source data

Throughout this report, we frequently refer to causal variants and benchmark simulation outcomes against them, raising the question of whether COJO should be considered a fine-mapping tool. In general, Manc-COJO outcomes should be interpreted as a model of variants proximal to true causal variants that are almost independently associated. In other words, although fine-mapping methods^28,29^ aim to pinpoint (within credible sets) the exact causal variant, Manc-COJO seeks to construct a model with nearly independent variants, where tagging variants close to causal ones suffice (Extended Data Fig. 6 and Supplementary Note 8). Therefore, COJO-type approaches should not be considered as bona fide fine-mapping methods.

This study has several limitations, some of which are inherent to the algorithm. We made several assumptions, as discussed in detail in Supplementary Method 1.1, which may be violated in practice. However, such violations are unlikely to substantially affect our overall conclusions. Other limitations arise from data quality and the limited availability of real data for certain ancestry groups, which reduced statistical power and likely contributed to the lack of statistical significance in real-data analyses. Nevertheless, the overall pattern of results was consistent across the traits and datasets examined and aligned with expectations from well-powered simulations. As GWAS sample sizes for non-EUR populations continue to increase, the application of Manc-COJO to crossancestry GWAS meta-analyses is expected to become increasingly valuable.

In summary, our study underscores the importance of collecting data from diverse ancestries, particularly as GWASs for some traits approach saturation in EUR ancestries^3^. It is essential to allocate resources to underrepresented populations during data collection efforts. With multiancestral data, approaches like Manc-COJO can better resolve LD and refine candidate variants for experimental validation. Given that LD blocks are smallest in AFR ancestry cohorts, GWASs in AFR ancestry cohorts will be the most cost-effective for optimization of Manc-COJO SNPs as well as for fine-mapping methods.

## Methods

### Ethics statement

This study was conducted in accordance with all applicable ethical regulations. All population databases used in this study had received prior approval from the appropriate ethics review boards and no additional ethical approval was required for the secondary analyses conducted in this study.

### Manc-COJO algorithm

Briefly, the Manc-COJO algorithm assumes GWAS summary statistics from two or more ancestries. The LD reference panel for each ancestry should closely match the cohort from which the GWAS was generated. The multiancestry COJO algorithm is summarized in seven steps (Fig. 1 and Supplementary Method 1) and, like GCTA-COJO, requires a pre-specified genome-wide significance threshold (here 5 × 10^−^^8^; Supplementary Note 9) and a pre-set collinearity threshold between SNPs (that is, maximum LD *r*^2^, here 0.9).

**Step 1:** conduct an inverse-variance meta-analysis (IVM) of GWAS summary statistics across all ancestries. The SNP with the smallest meta-analysis *P* value is selected for inclusion in the model.

**Step 2:** SNPs with an LD correlation above the pre-set collinearity threshold with any SNP already included in the model in any ancestry are removed in all ancestries. This assumes that the SNP selected from the LD block is the best SNP to represent the true causal SNP in this region.

**Step 3:** within each ancestry, a conditional analysis is performed for each SNP *i* not currently included and the conditional effect estimate is:

1 $${\hat{b}}_{i|{\hat{{\boldsymbol{\beta }}}}_{S}}={\hat{b}}_{i}-{{\hat{V}}_{i}}^{-\frac{1}{2}}{\hat{{\bf{r}}}}_{i,S}{\hat{R}}_{S}^{-1}{\hat{V}}_{\!S}^{\;\frac{1}{2}}{\hat{\bf{b}}}_{S}$$

where:

- ${\hat{{\bf{r}}}}_{i,S}$ is a 1 × *S* vector of observed (signed) LD correlations between SNP *i* and each of *S* SNPs already selected into the model (in the first iteration, only 1 SNP is in the model)
- ${\hat{R}}_{S}$ is an *S* × *S* matrix of observed (signed) LD correlations between *S* SNPs that are already in the model (in the first iteration, this reduces to a 1 × 1 matrix with the value = 1)
- ${\hat{{\bf{b}}}}_{{\bf{S}}}$ is an *S* × 1 vector of observed marginal effects of *S* SNPs in the model
- ${\hat{{\boldsymbol{\beta }}}}_{S}$ is an *S* × 1 vector of observed joint effects of *S* SNPs in the model
- ${\hat{b}}_{i}$ is the observed marginal effect of the SNP *i*
- ${\hat{V}}_{S}$ is an *S* × *S* diagonal matrix with *s*th elements $\hat{{V}_{s}}=\,2\hat{{p}_{s}}\hat{{q}_{s}}$ (assuming HWE)
- $${\hat{V}}_{i}=2{\hat{p}}_{i}{\hat{q}}_{i}$$ (assuming HWE).

**Step 4:** the conditional association statistic is estimated for each SNP (${z}_{i|\hat{{{\boldsymbol{\beta }}}_{S}}})$. As in GCTA-COJO, to avoid overfitting, a conservative approach is used:

2 $${z}_{(i|{\hat{{\boldsymbol{\beta }}}}_{S})}=\frac{{\hat{b}}_{i}-{{\hat{V}}_{i}}^{-\frac{1}{2}}{\hat{{\bf{r}}}}_{i,S}\,{{\hat{R}}_{S}}^{-1}{{\hat{V}}_{S}}^{\frac{1}{2}}{\hat{{\bf{b}}}}_{S}}{{\hat{S}}_{i}}$$

where ${\hat{S}}_{i}$ is the s.e. of the marginal effect of SNP *i*. The conditional *P* value is then calculated for each SNP and the SNP with the smallest meta-analysis *P* value is selected for inclusion in the model.

**Step 5:** after getting the *z*-statistics for each of the remaining SNPs, the SNP with the smallest *P* value is selected into the model (that is, the SNP with the largest *z*^2^). Afterwards, a joint analysis is performed as follows:

3 $${\hat{{\boldsymbol{\beta }}}}_{S+1}=\,{{\hat{V}}_{S+1}}^{-\frac{1}{2}}{{\hat{R}}_{{S}+1}}^{-1}{{\hat{V}}_{S+1}}^{\frac{1}{2}}{\hat{{\bf{b}}}}_{S+1}$$

Here the definitions of ${\hat{{\boldsymbol{\beta }}}}_{S+1}$, ${\hat{{\bf{b}}}}_{S+1}$, ${\hat{R}}_{S+1}$ and ${\hat{V}}_{S+1}$, are the same as defined in equation (1), except that now there are *S* + 1 SNPs in the model. As in GCTA-COJO, the estimation error of joint effects of *S* + 1 SNPs can be expressed as:

4 $$\mathrm{Var}({\hat{{\boldsymbol{\beta }}}}_{S+1})={\hat{\sigma }}_{{\rm{J}}}^{2}{{\hat{D}}_{S+1}}^{-\frac{1}{2}}{{\hat{R}}_{S+1}}^{-1}{{\hat{D}}_{S+1}}^{-\frac{1}{2}}$$

where ${\hat{D}}_{S+1}$ is an (*S* + 1) × (*S* + 1) diagonal matrix with *s*th elements ${\hat{D}}_{s}=2{\hat{p}}_{s}{\hat{q}}_{s}{\hat{n}}_{s}$ and ${\hat{n}}_{s}$ is the effective sample size of SNP *s*. Details on the estimation of ${\sigma }_{J}^{2}$ can be found in Supplementary Methods.

**Step 6:** an IVM of the joint effects of the SNPs included in the model is performed and any SNP with a meta-analysis *P* value for the joint effects—or with inclusion resulting in an adjusted ${R}^{2}$ (an optional criterion; Supplementary Note 10) that exceeds the pre-set threshold—is removed from the model.

**Step 7:** steps 2–6 are repeated until no SNP can be added to or removed from the model.

### Manc-COJO:FIX algorithm

We developed a pipeline, termed ‘FIX’, that enables users to retain specified variants in the COJO model by preventing their removal during the variable selection process. This facilitates the detection of ancestry-specific variants and supports functions such as the integration of wet-lab-validated evidence. Further details of the algorithm are provided in Supplementary Note 3.

### Simulations

Full details of the simulation framework are provided in Supplementary Method 2. Briefly, we used real genotype data from chromosome 22 of UKB participants to construct our simulations. The UKB recruited approximately 500,000 people with 88% recorded as white British. Three cohorts were defined: an AFR cohort (AFR1), comprising 6,901 unrelated participants of inferred AFR ancestry; and two EUR cohorts (EUR1 and EUR2), each including 6,901 randomly selected, unrelated individuals of inferred EUR ancestry, with no overlap or relatedness between EUR1 and EUR2^24^. EUR1 and EUR2 were then combined to form the EUR1_EUR2 cohort (*n* = 13,802) and AFR1 and EUR1 were combined to form the AFR1_EUR1 cohort (*n* = 13,802). The sample sizes of EUR1 and EUR2 were matched to AFR1 to reflect previous findings that combining equal-sized EUR and AFR ancestry cohorts maximize power for detecting causal associations in multiancestry meta-analyses^30,31^. Within these cohorts, chromosome 22 was partitioned into blocks following the definition in ref. ^32^ and we selected 38 blocks for simulation. In each block, we randomly designated 1, 5, 10 or 20 SNPs as causal variants and, to mitigate collinearity, LD correlations between all pairs of causal SNPs were restricted to ≤0.8. The effect sizes of these variants were simulated to be identical across ancestries, ensuring that SNP heritability for each causal SNP ranged between 0.5% and 1.5% in all EUR and AFR cohorts. Necessarily, if effect sizes are held constant across ancestries, then the contributions to variance explained will differ across ancestries depending on SNP allele frequency. For each combination of block and number of causal variants, the simulation was repeated 50×, drawing different sets of causal variants and effect sizes in each iteration. This design yielded 7,600 scenarios in total, reflecting 38 blocks, 4 levels of causal SNP count and 50 replicates of causal SNP sets and effect sizes.

Using these simulated causal SNP sets and effect sizes, phenotypes were generated within each of the defined cohorts. For each of the 7,600 scenarios, a GWAS was conducted in each cohort using PLINK 2.0 (--glm hide-covar cols=+a1freq). An IVM of summary statistics from EUR1 and AFR1 was then performed using customized R code to create the AFR1_EUR1 cohort. We compared outputs across the following GWAS and model combinations: Manc-COJO with EUR1 and AFR1 (denoted as ‘Manc-COJO → 6.9 k AFR + 6.9 k EUR’ in the main text), GCTA-COJO with EUR1_EUR2 (denoted as ‘GCTA-COJO → 13.8 k EUR’) and GCTA-COJO applied to multiancestral data using AFR1_EUR1 (denoted as ‘GCTA-COJO → 6.9 k AFR_6.9 k EUR’). Default settings were applied in all scenarios for both GCTA-COJO and Manc-COJO, with in-sample LD used throughout. For the AFR1_EUR1 cohort, in-sample LD was estimated from the merged genotypes of AFR1 and EUR1 using PLINK 1.9 (--bmerge). For multiancestral implementation of GCTA-COJO, we also applied a EUR-only LD reference (EUR1_EUR2 in this case), in addition to the in-sample mixed-ancestry LD (Table 1).

To assess model performance, we established several benchmarking criteria, described in detail in Supplementary Method 2. These included the number of COJO-selected SNPs that were true causal variants, the proportion of COJO-selected SNPs that were causal and metrics reflecting both power and false-positive discoveries. In addition, we assessed out-of-sample prediction accuracy using the estimated joint SNP effect sizes of COJO-selected SNPs in independent EUR (*n* = 13,800, randomly divided into 23 groups of 600) and AFR ancestry cohorts (*n* = 2,000, randomly divided into 20 groups of 100). Although the COJO algorithm is not designed to optimize out-of-sample risk prediction, comparing out-of-sample prediction using polygenic scores (PGSs) from COJO-selected SNPs provides a practical evaluation criterion that is relevant in both real-data analyses and simulation studies.

We also assessed the performance of different COJO algorithms under several nonidealized scenarios (Supplementary Method 2). Briefly, we repeated the above analyses under conditions where in-sample LD references were unavailable—using *n* = 503 EUR and a random sample of 503 AFR ancestry participants from the 1000 Genomes Project as the LD reference^33^. Additional scenarios included: biased sample sizes favoring the EUR ancestry population at a ratio of 10:1; ancestry-specific effect sizes (but with consistent directions of association across ancestries); ancestry-specific causal variants; and incomplete genotyping of causal variants, in which case we restricted the analysis to HapMap3 SNPs only^34^.

### Real-data analysis

We evaluated the performance of Manc-COJO in real-world data, using ancestry-specific GWAS summary statistics of EUR and AFR ancestry groups for four lipid traits chosen because of their large sample sizes for both EUR and AFR ancestry groups. Global Lipids Genetics Consortium (GLGC) GWAS summary statistics (which excluded the UKB) were available for HDL-C, LDL-C, TC and log(transformed) log(TG)^35^. Only common SNPs (minor allele frequency ≥0.01) present in both ancestries and biallelic SNPs were retained for analysis. To allow for out-of-sample prediction analysis into the UKB, only SNPs available in the UKB across both ancestries and with a minor allele frequency ≥0.01 among unrelated AFR ancestry participants (*n* = 6,901; genetic relatedness <5%) were retained for Manc-COJO analysis.

For each trait, Manc-COJO input files were GWAS summary statistics (one for each ancestry) and corresponding ancestry-specific LD reference panels (that is, a randomly sampled set of 69,010 unrelated EUR ancestry individuals and 6,901 unrelated AFR ancestry individuals from the UKB). Default parameters were applied: a window size of 10 Mb (assuming SNPs >10 Mb apart to be independent), a collinearity threshold of *r*^2^ < 0.9 and a significance threshold of *P* < 5 × 10^−^^8^, consistent with the original GCTA-COJO.

We benchmarked Manc-COJO against GCTA-COJO in both EUR-only meta-analysis and IVM combining multiple ancestries (multiancestry meta-analysis). GCTA-COJO was applied under three distinct scenarios: (1) single-ancestry meta-analysis with the EUR-ancestry-only LD reference; (2) multiancestry meta-analysis with the ancestry-matched LD reference; and (3) multiancestry meta-analysis with the EUR-ancestry-only LD reference (Supplementary Tables 1 and 2).

To enable a fair comparison based on equal sample sizes across scenarios, we generated GWAS summary statistics for each lipid trait using 90,000 unrelated UKB participants of inferred EUR ancestry (Supplementary Method 3 and Supplementary Table 2). These UKB results were then meta-analyzed with the EUR-specific GWAS from the GLGC to construct a EUR-only meta-analysis.

LD reference panels for GCTA-COJO-based methods were created using PLINK’s --bmerge function, from either a merged cohort comprising 69,010 individuals of EUR ancestry and 6,901 individuals of AFR ancestry (ancestry-matched LD reference) or a merged cohort of 69,010 and 6,901 individuals both of EUR ancestry (EUR-only LD reference).

Given that the GLGC GWAS was heavily skewed toward EUR ancestry—with an approximate 10:1 ratio of EUR-to-AFR participants after the exclusion of UKB samples—we conducted a downsampling analysis to investigate a more balanced ancestral representation (approximately 1:1). To do this, we employed GWAS summary statistics derived from the GLGC AFR cohort (*n* ~ 0.09 M) and then used a UKB EUR subset of the same *n*. For the single-ancestry analyses, GWAS summary statistics were generated from 180,000 UKB participants of EUR ancestry for each lipid trait, following the same analytical pipeline used in the full-scale analysis (Supplementary Table 3 and Supplementary Note 2). The multiancestry LD reference panel consisted of 6,901 unrelated EUR and 6,901 unrelated AFR individuals, whereas the EUR-only LD reference comprised 13,802 unrelated EUR individuals, excluding those included in the GWAS. In this analysis, we again benchmarked Manc-COJO applied to multiancestry data against three alternative approaches: (1) GCTA-COJO applied to meta-analyzed multiancestry data using an ancestry-matched LD reference; (2) GCTA-COJO applied to meta-analyzed multiancestry data using a EUR-only LD reference; and (3) GCTA-COJO applied to single-ancestry GWAS using a EUR-only LD reference.

To evaluate objectively the Manc-COJO SNP sets, we used out-of-sample predictive performance. We used 47,066 unrelated UKB participants of inferred EUR ancestry who were included in neither the LD reference panel nor the GWAS analyses, as well as 3,000 participants of inferred AFR ancestry from the UKB. Residuals for all lipid traits were computed using the same covariate adjustments as applied in the GLGC GWAS analyses (Supplementary Method 3). SNP effect sizes, estimated from jointly fitting all SNPs identified by each Manc-COJO and GCTA-COJO model using the --cojo-joint function, were used as SNP allele weights in PGSs. This was performed separately for the EUR-only meta-analysis and the AFR ancestry GWAS summary statistics from the GLGC (Supplementary Tables 2 and 3). PGSs were generated using PLINK (‘--score’ command). To estimate the prediction performance, we randomly divided 47,066 unrelated UKB EUR participants for lipid traits into 50 groups and 3,000 unrelated UKB AFR participants into 20 groups, with one group having more individuals if the sample size was not evenly divisible. Within each group, the out-of-sample *R*^2^ values from a simple linear regression (residual ~ PGS + error) was calculated and averaged across groups for each trait and ancestry, and the s.d. of the estimate was computed.

We repeated our analysis of out-of-sample predictive performance using the All of Us cohort. As SNPs in the All of Us data are aligned to the GRCh38 reference genome, we first constructed a conversion table between SNP names and genomic coordinates in the UKB (GRCh37) and All of Us (GRCh38) cohorts using the University of California, Santa Cruz LiftOver tool^36^. A small proportion of SNPs (~0.3 M out of 8.5 M SNPs with minor allele frequency ≥0.01 in the UKB) could not be successfully converted from GRCh37 to GRCh38 coordinates. To ensure a fair comparison, we repeated the Manc-COJO and GCTA-COJO analyses using the approximately 1-M-sample version of the summary statistics (~0.9 M EUR + 0.09 M AFR or EUR) as described above, but retained only SNPs that were present in both the UKB and the All of Us cohorts at the start of the analysis. For phenotype generation in the All of Us cohort, we extracted lipid traits and applied adjustments as detailed in Supplementary Method 3. We retained EUR ancestry samples (~80,000 per trait) and AFR ancestry samples (~25,000 per trait), with ancestry defined according to the file: ‘gs://fc-aou-datasets-controlled/v8/wgs/short_read/snpindel/aux/ancestry/ancestry_preds.tsv’. Samples were divided into groups of 1,000 and we applied the same protocol used in the UKB to compute PGSs.

### Software and packages

Summary statistics were processed using R v4.3.2 with the data.table package v1.17.8. Causal variants and effect sizes were simulated using customized R scripts, with genotype data imported using the genio package v1.1.2. Simulated GWASs were performed using PLINK 2.0 v2.00a2.3. Meta-analysis was conducted using customized scripts that formatted the resulting summary statistics for input into GCTA-COJO. Conditional and joint analyses were performed using Manc-COJO v1.1.0 and GCTA v1.93.2β and PGSs were calculated using PLINK v1.90b6.21. The regional association plot in Fig. 5 was generated using the R package locuszoomr v0.3.8 with gene annotations from EnsDb.Hsapiens.v75 v2.99.0. All other plots were generated using Python v3.11.3 with the packages matplotlib v3.11.1, NumPy v2.5.1 and pandas v3.0.5. All customized code developed for this study is available on GitHub (https://github.com/light156/multi-ancestry-COJO). and archived on Zenodo^37^ (see ‘Code availability’ below).

### Reporting summary

Further information on research design is available in the Nature Portfolio Reporting Summary linked to this article.

## Online content

Any methods, additional references, Nature Portfolio reporting summaries, source data, extended data, supplementary information, acknowledgements, peer review information; details of author contributions and competing interests; and statements of data and code availability are available at https://doi.org/10.1038/s41588-026-02799-1.

## Supplementary information

Supplementary Information Supplementary Methods 1–3, Notes 1–10, Tables 1–8 and Figs. 1–11.

Reporting Summary

Peer Review File

Supplementary Data 1 Source data for Supplementary Fig. 1.

Supplementary Data 2 Source data for Supplementary Fig. 2.

Supplementary Data 3 Source data for Supplementary Fig. 3.

Supplementary Data 4 Source data for Supplementary Fig. 4.

Supplementary Data 5 Source data for Supplementary Fig. 5.

Supplementary Data 6 Source data for Supplementary Fig. 6.

Supplementary Data 7 Source data for Supplementary Fig. 7.

Supplementary Data 8 Source data for Supplementary Fig. 8.

Supplementary Data 9 Source data for Supplementary Fig. 9.

Supplementary Data 10 Source data for Supplementary Fig. 10.

Supplementary Data 11 Source data for Supplementary Fig. 11.

## Source data

Source Data Fig. 2 Source data for Fig. 2.

Source Data Fig. 3 Source data for Fig. 3.

Source Data Fig. 4 Source data for Fig. 4.

Source Data Fig. 5 Source data for Fig. 5.

Source Data Extended Data Fig. 1 Source data for Extended Data Fig. 1.

Source Data Extended Data Fig. 2 Source data for Extended Data Fig. 2.

Source Data Extended Data Fig. 3 Source data for Extended Data Fig. 3.

Source Data Extended Data Fig. 4 Source data for Extended Data Fig. 4.

Source Data Extended Data Fig. 5 Source data for Extended Data Fig. 5.

Source Data Extended Data Fig. 6 Source data for Extended Data Fig. 6.

## Data Availability

UKB and All of Us data are available through formal application to the UKB (https://www.ukbiobank.ac.uk) and the All of Us research hub (https://www.researchallofus.org), respectively. GWAS summary statistics for the four lipid traits analyzed in this study are publicly available from the GLGC (https://csg.sph.umich.edu/willer/public/glgc-lipids2021/). The 1000 Genomes Project genotype data used for LD reference comparisons are available via figshare at https://doi.org/10.6084/m9.figshare.20802700 (ref. ^38^). Quality-controlled summary statistics used in this study are available via Zenodo at https://doi.org/10.5281/zenodo.21704762 (ref. ^37^). Source data are provided with this paper.

## Code availability

Manc-COJO is implemented as a publicly available software package. The C++ source code and precompiled executable binaries are available at https://github.com/light156/multi-ancestry-COJO. Usage instructions and full documentation are provided at https://light156.github.io/multi-ancestry-COJO-docs. All customized code developed as part of this project is also available via GitHub (https://github.com/light156/multi-ancestry-COJO) and Zenodo at https://doi.org/10.5281/zenodo.21704762 (ref. ^37^).
