# Supplementary material for: Multiancestry conditional and joint analysis applied to GWAS summary statistics
Source: Nat Genet. 2026 Oct 20;58(11):3019–27. doi: 10.1038/s41588-026-02799-1 (PMC13754604; doi:10.1038/s41588-026-02799-1)
Supplement: Supplementary file 1 [file 41588_2026_2799_MOESM1_ESM.pdf]

nature genetics

Supplementary information

https://doi.org/10.1038/s41588-026-02799-1

Multiancestry conditional and joint analysis applied to GWAS summary statistics

In the format provided by the authors and unedited

Table of Contents

Supplementary Methods 1. Important considerations in Manc-COJO model **3**

1.1 Concepts and assumptions **3**

1.1.1 Modelling true effect in an idealised population setting 3

1.1.2 Modelling GWAS marginal effects in an idealised population setting 4

1.1.3 Modelling joint effects in an idealised population setting 4

1.1.4 Modelling conditional effects in an idealised population setting 5

1.1.5 Assumption of a homogenous LD structure 6

1.2 Addressing the challenges of real-world data **6**

1.2.1 Considerations in dealing with real-world data 7

1.2.2 Overview of single-ancestry COJO algorithm 8

1.2.3 Extending COJO analysis into multi-ancestry settings 10

Supplementary Methods 2. Simulations **13**

2.1 Simulation settings **13**

2.1.1 Simulation cohort 13

2.1.2 Partitioning the genome 14

2.1.3 Simulation of causal SNPs 14

2.1.4 Simulation of phenotypes 14

2.1.5 GWAS simulation, and benchmark against existing method 15

2.1.6 Model performance measurement metrics 15

2.2 Simulation sensitivity analysis **16**

2.3 Simulation for the FIX function **16**

Supplementary Methods 3. Real data analysis **18**

3.1 Data quality control **18**

3.2 Data input **18**

3.3 Out-of-sample prediction in UK Biobank **19**

3.4 Out-of-sample prediction in All of Us **20**

Supplementary Notes 1. Simulation sensitivity analysis **22**

Supplementary Notes 2. Down-sampled analysis **23**

Supplementary Notes 3. The Manc-COJO:FIX function **24**

3.1 When to use Manc-COJO:FIX **24**

3.2 Manc-COJO:FIX algorithms **24**

3.3 Interpretation of the Manc-COJO:FIX function outcomes in the context of ancestry specific associations **26**

Supplementary Notes 4. Algorithmic improvements over original COJO **27**

Supplementary Note 4.1 More robust outputs **27**

Supplementary Note 4.2 Improved computation efficiency **27**

Supplementary Note 4.3 Dealing with data missingness 28
Supplementary Notes 5. Insight into Manc-COJO vs GCTA-COJO output 30
Supplementary Notes 6. Meta-analysis, mega-analysis, and admixed population 33
Supplementary Notes 7. Decision on cohort inclusion in the analysis 35
7.1 Extension of Manc-COJO to multiple ancestry groups 35
7.2 Inclusion of a smaller cohort 35
Supplementary Notes 8. COJO and fine-mapping tools 38
Supplementary Notes 9. Stability of the Manc-COJO selection algorithm 40
Supplementary Notes 10. Choice of R^2 parameter 41
Supplementary Tables and Figures 42
References 61

Supplementary Methods 1. Important considerations in Manc-COJO model

In this section, we first review the assumptions underlying the COJO models. We then detail our algorithms and outline the step-by-step model selection process. Finally, we demonstrate the validity of the algorithms implemented in our method through theoretical derivations.

1.1 Concepts and assumptions

In GWAS and post-GWAS analyses, there are three SNP effect size concepts: the causal effect size (β_c), the marginal effect size (b_m), and the effect size from the joint analysis of SNPs (β_J).

1.1.1 Modelling true effect in an idealised population setting

We denote β_c as the causal effect size on the phenotype.

We make three assumptions about causal effects:

(1) β_c of SNPs act on the phenotype additively, which means that for a specific SNP, each copy of the alternative allele adds, on average, β_c to the phenotype, and the effect of a specific SNP will not affect the effect of another SNP.

(2) β_c are treated as fixed-effect parameters rather than random draws from a distribution, consistent with the original GCTA-COJO framework[1].

(3) The number of causal variants, and their effect sizes on **standardised** phenotypes, are identical in different ancestry groups. In other words, under an additive model, if a variant is causal in one ancestry, it is also causal in another ancestry. However, allele frequencies of causal SNPs can differ between ancestry groups because of genetic drift, bottleneck or differential selective pressure[2]. We later relax this assumption by including ancestry-specific variants.

These assumptions can be violated in various ways. For instance, dominant or recessive SNP effects may exist[3] and/or may interact with other SNPs[4], but these can only be identified in the context of large SNP effects. Previous research has shown that the additive effect model explains most genetic variation in complex traits[5,6].

Our data generation model assumes that phenotypes of **all** individuals in a population (size *N*) depend on causal SNP effects according to the following equation:

$$\mathbf{y} = \mathbf{X}_C \boldsymbol{\beta}_C + \mathbf{e}_C \qquad (1)$$

where

- $\mathbf{y}$ is an *N*x1 vector of **standardised** residuals of phenotypes (after regressing out all covariates)
- $\mathbf{X}_C$ is an *N*x*C* **mean-centred** matrix of *C* genotypes of causal variants shared across ancestry groups (mean-centred within each ancestry group for multi-ancestry datasets)

- $\boldsymbol{\beta_C}$ is a *C*x1 vector of causal effects
- $\mathbf{e_C}$ is an *N*x1 vector of error terms, which is normally distributed under N(0,σ_e^2)

Importantly, causal variants and their effect sizes are typically unknown for several reasons. For example, not all causal SNPs are genotyped nor imputed from a SNP-array. Even with whole genome sequencing data, there are still gaps (for example, regions near the centromere and telomere[7]). LD adds complexity in identifying the causal variants, especially when there are multiple causal variants within a region of correlated SNPs. In following sections, we will model what is observable in practice, and link it to the unobservable causal effects.

1.1.2 Modelling GWAS marginal effects in an idealised population setting

In GWAS analysis, one SNP at a time is tested for association with the phenotype. Here, we commence the derivation by assuming access to genetic data for **every individual** in the population; accordingly, all parameters are treated as **population parameters**.

Let $\mathbf{x_m}$ be an *N*x1 mean-centred vector of genotypes for the SNP *m* to be tested in the GWAS analysis, and b_m the marginal effect size of the SNP m.

As the genotype and error are independent (*i.e.* $\mathbf{x}'_{\boldsymbol{m}}\mathbf{e}_{\boldsymbol{C}} = 0$), $\mathbf{x}'_{\boldsymbol{m}}\mathbf{y} = \mathbf{x}'_{\boldsymbol{m}}\mathbf{X_C}\boldsymbol{\beta}_\mathbf{C}$.
It follows that,

$$b_m = (\mathbf{x}'_{\boldsymbol{m}}\mathbf{x}_{\boldsymbol{m}})^{-1}\mathbf{x}'_{\boldsymbol{m}}\mathbf{y} \qquad (2)$$

Notably, this expression is derived under an idealised population assumption, and we discuss the estimation of b_m in Section 1.2.

Now, let p_m to be the minor allele frequency of the SNP *m*, $q_m = 1 - p_m$. Based on the definition of binomial variance, and under the Hardy-Weinberg equilibrium (HWE), $\mathbf{x}'_{\boldsymbol{m}}\mathbf{x}_{\boldsymbol{m}}$ = $2p_m q_m N$. Let us denote $D_m = 2p_m q_m N$.

Applying equation (2) into equation (1), we get

$$b_m = \frac{\mathbf{x}'_{\boldsymbol{m}}\mathbf{X}_{\boldsymbol{C}}\boldsymbol{\beta}_{\boldsymbol{C}}}{D_m} = \sum_{c=1}^{C} r_{m,c}\beta_c\sqrt{\frac{p_c q_c}{p_m q_m}} \qquad (3)$$

where, $r_{m,c}$ denotes the (signed) LD correlation between SNP *m* and the *c*-th causal SNP. In other words, the value of true marginal effect size (b_m) for the SNP m in the GWAS analysis, can be viewed as a weighted sum of true effect size of all causal SNPs, where the weights are the LD correlations between the SNP m and causal SNPs, adjusted for minor allele frequencies.

1.1.3 Modelling joint effects in an idealised population setting

In a joint analysis, any set of *J* SNPs can be fitted together, yielding the corresponding true joint effects (assuming we have genetic data for every individual in the population). The major benefit of considering SNPs jointly is to account for the LD correlation between these SNPs.

Joint effects of *J* SNPs can be related to the true marginal effects of those *J* SNPs and the LD correlation between those *J* SNPs:

$$\boldsymbol{\beta}_{\boldsymbol{J}} = \mathbf{D}_{\boldsymbol{J}}^{-\frac{1}{2}}\mathbf{R}_{\boldsymbol{J}}^{-1}\,\mathbf{D}_{\boldsymbol{J}}^{\frac{1}{2}}\mathbf{b}_{\boldsymbol{M}} \qquad (4)$$

Here,

- $\mathbf{D}_{\boldsymbol{J}}$ is a *JxJ* diagonal matrix with *j*-th elements being $D_{j,j} = 2p_j q_j N$
- $\boldsymbol{\beta}_{\boldsymbol{J}}$ is a *Jx1* vector of true **joint** effect sizes when fitting those *J* SNPs jointly in a model
- $\mathbf{b}_{\boldsymbol{M}}$ is a *Jx1* vector of true **marginal** effect sizes
- $\mathbf{R}_{\boldsymbol{J}}$ is a *JxJ* true LD correlation matrix between *J* SNPs that are fitted jointly in the model

Applying equation (3) into equation (4), gives

$$\boldsymbol{\beta}_{\boldsymbol{J}} = \mathbf{D}_{\boldsymbol{J}}^{-\frac{1}{2}}\,\mathbf{R}_{\boldsymbol{J}}^{-1}\mathbf{r}_{\boldsymbol{J},\boldsymbol{C}}\mathbf{D}_{\boldsymbol{C}}^{\frac{1}{2}}\boldsymbol{\beta}_{\boldsymbol{C}} \qquad (5)$$

where,

- $\mathbf{D}_{\boldsymbol{J}}$ is a *JxJ* diagonal matrix with *j*-th elements being $D_{j,j} = 2p_j q_j N$
- $\mathbf{r}_{\boldsymbol{J},\boldsymbol{C}}$ is a *JxC* true LD correlation matrix between *J* SNPs that are fitted jointly in the model and *C* causal SNPs
- $\mathbf{D}_{\boldsymbol{C}}$ is a *CxC* diagonal matrix with *c*-th elements being $D_{c,c} = 2p_c q_c N$
- $\boldsymbol{\beta}_{\boldsymbol{C}}$ is a *Cx1* vector of **true** effect sizes of *C* causal SNPs

Now, let

- $\mathbf{V}_{\boldsymbol{J}}$ be a *JxJ* diagonal matrix with *j*-th elements being $V_{j,j} = 2p_j q_j$
- $\mathbf{V}_{\boldsymbol{C}}$ be a *CxC* diagonal matrix with *c*-th elements being $V_{c,c} = 2p_c q_c$

Assuming an idealised population, Equation (5) can be simplified to:

$$\boldsymbol{\beta}_{\boldsymbol{J}} = \mathbf{V}_{\boldsymbol{J}}^{-\frac{1}{2}}\,\mathbf{R}_{\boldsymbol{J}}^{-1}\mathbf{r}_{\boldsymbol{J},\boldsymbol{C}}\mathbf{V}_{\boldsymbol{C}}^{\frac{1}{2}}\boldsymbol{\beta}_{\boldsymbol{C}} \qquad (6)$$

When all *C* causal SNPs are fitted in a model, $\mathbf{R}_{\boldsymbol{J}} = \mathbf{r}_{\boldsymbol{J},\boldsymbol{C}}$, $\mathbf{V}_{\boldsymbol{J}} = \mathbf{V}_{\boldsymbol{C}}$. It follows that $\boldsymbol{\beta}_{\boldsymbol{J}} = \boldsymbol{\beta}_{\boldsymbol{C}}$**.** In other words, when all causal SNPs are included jointly in a model and all the assumptions described above are satisfied, the true joint SNP effects will equal the causal SNP effects.

1.1.4 Modelling conditional effects in an idealised population setting

Having modelled the marginal and joint effects, we can now define an additional quantity: the conditional effect. Given a set of SNPs (set *S*), we fit these SNPs jointly into a model as in (4), and obtain the residual:

$$\mathbf{y}_{\boldsymbol{S}} = \mathbf{y} - \mathbf{X}_{\boldsymbol{S}}\boldsymbol{\beta}_{\boldsymbol{S}}$$

For each SNP i not included in the model, the conditional effect of SNP i given set *S*, denoted $b_{i|\boldsymbol{\beta}_S}$, is essentially the marginal effect of SNP *i* in the following regression:

$$\mathbf{y}_{\boldsymbol{S}} = \mathbf{x}_{\boldsymbol{i}} b_{i|\boldsymbol{\beta}_S} + \mathbf{e}_{\boldsymbol{S}}$$

Intuitively, the conditional effect represents the remaining contribution of SNP *i* to the phenotype after adjusting for the effects of SNPs in set *S*.

The conditional effect $b_{i|\boldsymbol{\beta}_S}$ can be expressed as follows:

$$b_{i|\boldsymbol{\beta}_S} = b_i - V_i^{-\frac{1}{2}} \mathbf{r}_{i,S} \mathbf{V}_S^{\frac{1}{2}} \boldsymbol{\beta}_S$$
$$= b_i - V_i^{-\frac{1}{2}} \mathbf{r}_{i,S} \mathbf{R}_S^{-1} \mathbf{V}_S^{\frac{1}{2}} \mathbf{b}_S \qquad (7)$$

where,

- $\mathbf{r}_{i,S}$ is a 1 x *S* vector of LD correlations between SNP *i* and each of *S* SNPs that are already selected into the model (set S)
- $\boldsymbol{\beta}_S$ is an *S* x 1 vector of true **joint effects** of the *S* SNPs in set *S* in the model
- $\mathbf{R}_S^{-1}$ is an *S* x *S* matrix of LD correlations between S SNPs that are already in the model
- $\mathbf{b}_S$ is an *S* x 1 vector of true **marginal effects** of *S* SNPs in the model
- b_i is the true marginal effect of the SNP *i*
- $\mathbf{V}_S$ is an *S* x *S* diagonal matrix with *s*-th elements $V_s = 2p_s q_s$ (Assuming HWE)
- $V_i = 2p_i q_i$ (Assuming HWE)

1.1.5 Assumption of a homogenous LD structure

It is important to note that all derivations above implicitly assume a homogeneous LD structure within the population. Consider a scenario in which a population (denoted by the subscript *com*) is not homogeneous, but consists of two sub-populations (denoted by subscripts *1* and *2*) **com**bined together. Each sub-population is internally homogeneous, yet they differ in allele frequencies at the same SNPs, resulting in distinct correlation structures between the sub-populations. In such scenario, the genotypic variance of SNP *i* in the combined population ($V_{com,i}$) will no longer be $2p_{com,i}q_{com,i}$, but rather

$$V_{com,i} = 2p_{1,i}q_{1,i}w_1 + 2p_{2,\mathrm{i}}q_{2,\mathrm{i}}w_2 + 4w_1w_2(p_{1,\mathrm{i}} - p_{2,\mathrm{i}})^2 \qquad (8)$$

where w_1 and w_2 are the proportion of sub-population 1 and 2 in the combined population, respectively. In other words, the Hardy–Weinberg equilibrium (HWE) assumption is violated when the population is not homogeneous.

Let $V_{a,i}$ be the genotypic variance of SNP *i* in the population *a*, where $V_{a,i} = 2p_{a,i}q_{a,i}$, and $r_{\mathrm{a},i,k}$ be the (signed) LD correlation between SNP *i* and SNP *k*. The LD correlation between SNP *i* and SNP *k* in the combined population, can be expressed as:

$$r_{\mathrm{com},i,k} = w_1 r_{1,\mathrm{i},k}\sqrt{\frac{V_{1,i}V_{1,k}}{V_{\mathrm{com},i}V_{\mathrm{com},k}}} + w_2 r_{2,i,k}\sqrt{\frac{V_{2,i}V_{2,k}}{V_{\mathrm{com},i}V_{\mathrm{com},k}}} + \frac{4w_1w_2(p_{1,\mathrm{i}}-p_{2,\mathrm{i}})(p_{1,\mathrm{k}}-p_{2,\mathrm{k}})}{\sqrt{V_{\mathrm{com},i}V_{\mathrm{com},k}}} \qquad (9)$$

It is clear that $r_{\mathrm{com},i,k} = r_{1,i,k}$ will hold for all SNP pairs *i* and *k* only in a homogeneous population (*i.e.* $w_1 = 1, w_2 = 0$, $V_{1,i} = V_{\mathrm{com},i}$, $V_{1,k} = V_{\mathrm{com},k}$, $p_{1,\mathrm{i}} = p_{2,\mathrm{i}}$, and $p_{1,\mathrm{k}} = p_{2,\mathrm{k}}$)

1.2 Addressing the challenges of real-world data

In an idealised scenario, where genetic data are available for all individuals in a population, the population is homogeneous, and all model assumptions hold, fitting all causal SNPs jointly would recover the causal effect sizes. In practice, such conditions never exist, and some assumption violations are beyond our control. Therefore, the aim of our algorithm (and, arguably, all COJO- or fine-mapping–type algorithms) is to identify a set of SNPs that

approximates the causal variants—in both number and genomic location—while controlling for manageable sources of error and recognising inherent limitations of the model. To achieve this aim, we can implement an extended version of conditional and joint analyses in each iteration of the algorithm. As in GCTA-COJO[1], LD correlations between SNPs can be estimated from a reference sample, with allele frequencies and marginal effect sizes available from GWAS (meta-analysis) summary statistics. However, when using summary statistics several additional quality control procedures are needed to make sure results are comparable to scenarios where individual level genotype data are available.

1.2.1 Considerations in dealing with real-world data

- *Estimation of GWAS marginal effects*

In practice, it is impossible to obtain genotype data from every individual in a population. Consequently, we analyse a sample drawn from the population and perform an association test on the observed data. It follows from Equation (2) that the ordinary least-squares estimator of the marginal effect $\hat{b}_m$ for SNP m can be written as

$$\hat{b}_m = (\mathbf{x}'_m\mathbf{x}_m)^{-1}\mathbf{x}'_m\mathbf{y} = b_m + e_m$$

Here, $\mathbf{y}$ now denotes the N_m x 1 vector of **observed** phenotypes, and $\mathbf{x_m}$ now denotes corresponding N_m x 1 vector of **observed** genotypes at SNP m, where N_m is the sample size for SNP m. It follows that,

$$\mathrm{E}\left[\hat{b}_m\right] = b_m$$

- *Phenotypic variance*

In our model we assumed phenotypic variance to be 1 across studies. However, this may not be true in practice (even when traits are described as standardised this is often prior to accounting for covariates). Therefore, as in GCTA-COJO, the phenotypic variance in each study is estimated from GWAS summary statistics. For each SNP *i*, the estimate of $\boldsymbol{y}'\boldsymbol{y}$ can be estimated from:

$$\hat{\boldsymbol{y}}'\hat{\boldsymbol{y}} = \hat{D}_i\hat{S}_i^2(n_i - 1) + \hat{D}_i\hat{b}_i^2$$

where $\hat{S}_i^2$ is the squared standard error of the marginal effect, $\hat{b}_i$ is observed marginal effect, n_i is the per-SNP sample size of SNP i (*i.e.* not the population size.

Phenotypic variance (V_P) can then be estimated by:

$$\hat{V}_P = \hat{D}_i\hat{S}_i^2 + \frac{\hat{D}_i\hat{b}_i^2}{n_i - 1} \qquad (10)$$

In our analyses, we took the median of $\hat{V}_P$ across all SNPs in the GWAS summary statistics as the estimate of the phenotypic variance.

- *Effective sample size*

As discussed in the GCTA-COJO paper, when data sets contributing to the GWAS results include relatives, the reported sample size will be larger than the effective sample size. In our analyses we estimated the per-SNP sample size for any SNP *i* using the equation[1]:

$$\hat{n}_i = \frac{\hat{V}_P - 2\hat{p}_i\hat{q}_i\hat{b}_i^2}{2\hat{p}_i\hat{q}_i\hat{S}_i^2} + 1 \qquad (11)$$

- *Per-SNP sample size*

In real data sets, per-SNP sample size can be significantly different between SNPs. Within a single data set differences in sample size between SNPs occur because of insufficient SNPs in a region to allow accurate imputation, which in turn may reflect genotype missingness. In GWAS meta-analyses different sub-studies may contribute different SNPs which can generate very large differences in sample sizes between SNPs. We suggest exclusion of SNPs to avoid extreme differences in per-SNP sample sizes, and outline strategies for handling genotype missingness among the remaining SNPs in **Supplementary Note 4.3**.

- *Choice of reference sample to estimate LD correlation matrix*

In Section 1.1.5, we examined the assumption of a homogeneous LD structure within a GWAS cohort. When GWAS summary statistics are obtained from meta-analyses of multiple cohorts, or when they are combined with an external LD reference cohort (*i.e.,* out-of-sample LD), as in GCTA-COJO, an additional layer of complexity is introduced. For the analysis to yield reliable outcomes, all cohorts contributing to the meta-analysis within a single inferred ancestry should exhibit a broadly similar LD structure (*i.e.,* heterogeneity may exist between GWAS meta-analysis cohorts but not within each cohort). Furthermore, it is essential to ensure that the LD structure of the GWAS cohort and that of the LD reference cohort are sufficiently similar to produce robust results.

Ideally, the LD estimated from the same sample used to generate the GWAS should also be employed in COJO analyses. For this reason, we support sharing of LD matrices together with GWAS summary statistics thereby facilitating information sharing when access to raw data is restricted. When the use of out-of-sample LD is unavoidable, its selection may be informed by prior knowledge or by approaches such as principal component (PC) analysis, which lies beyond the scope of this paper.

In addition, we recommend testing for differences in allele frequency between the reference and GWAS cohorts. When the sample size is large enough, the distribution of allele frequency of SNP *i* can be approximated by a normal distribution. $\hat{p}_i \sim \mathrm{N}\left(p, \frac{pq}{2N}\right)$. Hence, we can consider removing SNPs that are significantly different at specified threshold (for example, $p = 5\text{x}10^{-8}$) between the GWAS (subscript *GWAS*) and reference data (subscript *ref*) sets based on the following z-score:

$$Z = \frac{\hat{p}_{ref} - \hat{p}_{GWAS}}{\sqrt{\frac{\hat{p}_{ref}\hat{q}_{ref}}{2n_{ref}} + \frac{\hat{p}_{GWAS}\hat{q}_{GWAS}}{2n_{GWAS}}}} \qquad (12)$$

Another important consideration is the size of the reference sample. Empirical evidence suggest that reference sample sizes > 5,000 will generate relatively accurate LD correlation estimates[1].

1.2.2 Overview of single-ancestry COJO algorithm

In our study, we re-derived the equations used in the original GCTA-COJO so that the LD correlation matrix could be used as the LD reference, in addition to individual-level genotype data.

In the first iteration of the GCTA-COJO algorithm, the SNP with the smallest p-value is selected into the model. Conditional on this SNP, the conditional z-statistics of all other SNPs (taking SNP *i* as an example) are then calculated as:

$$z_{i|\widehat{\boldsymbol{\beta}}_{\boldsymbol{S}}} = \frac{\hat{b}_i - \hat{V}_i^{-\frac{1}{2}} \hat{\mathbf{r}}_{\boldsymbol{i,S}} \widehat{\mathbf{R}}_{\boldsymbol{S}}^{-1} \widehat{\mathbf{V}}_{\boldsymbol{S}}^{\frac{1}{2}} \hat{\mathbf{b}}_{\boldsymbol{S}}}{\hat{S}_i} \qquad (13)$$

where,

- $\hat{\mathbf{r}}_{\boldsymbol{i,S}}$ is a 1 x *S* vector of **observed** LD correlations between SNP *i* and each of the *S* SNPs already selected into the model (in the first iteration, only 1 SNP is in the model)
- $\widehat{\mathbf{R}}_{\boldsymbol{S}}$ is an *S* x *S* matrix of **observed** LD correlations between S SNPs that are already in the model (in the first iteration, this reduces to a 1×1 matrix with the value equal to 1)
- $\hat{\mathbf{b}}_{\boldsymbol{S}}$ is an S x 1 vector of **observed marginal effects** of *S* SNPs in the model
- $\widehat{\boldsymbol{\beta}}_{\boldsymbol{S}}$ is an S x 1 vector of **observed joint effects** of *S* SNPs in the model
- $\hat{b}_i$ is the **observed** marginal effect of the SNP *i*
- $\widehat{\mathbf{V}}_{\boldsymbol{S}}$ is an *S* x *S* diagonal matrix with *s*-th elements $\hat{V}_s = 2\hat{p}_s\hat{q}_s$ (assuming HWE)
- $\hat{V}_i = 2\hat{p}_i\hat{q}_i$ (assuming HWE)
- As in GCTA-COJO, to avoid over-fitting, a conservative approach is used to get the conditional p-value, where $\hat{S}_i$ denotes the standard error of the observed marginal effect of SNP *i*.

After getting the z-statistics for each of the remaining SNPs, the SNP with the smallest p-value is selected into the model (*i.e.* the SNP with largest z^2). Afterwards, a joint analysis is performed as follows:

$$\widehat{\boldsymbol{\beta}}_{\boldsymbol{S+1}} = \widehat{\mathbf{V}}_{\boldsymbol{S+1}}^{-\frac{1}{2}} \widehat{\mathbf{R}}_{\boldsymbol{S+1}}^{-1} \widehat{\mathbf{V}}_{\boldsymbol{S+1}}^{\frac{1}{2}} \hat{\mathbf{b}}_{\boldsymbol{S+1}} \qquad (14)$$

Here, the definition of $\widehat{\boldsymbol{\beta}}_{\boldsymbol{S+1}}$, $\hat{\mathbf{b}}_{\boldsymbol{S+1}}$, $\widehat{\mathbf{R}}_{\boldsymbol{S+1}}$, and $\widehat{\mathbf{V}}_{\boldsymbol{S+1}}$, are the same as defined in equation (13), except that now there are *S*+1 SNPs in the model. As in GCTA-COJO, the estimation error of joint effects of *S*+1 SNPs can be expressed as:

$$\text{Var}(\widehat{\boldsymbol{\beta}}_{\boldsymbol{S+1}}) = \hat{\sigma}_J^2 \widehat{\mathbf{D}}_{\boldsymbol{S+1}}^{-\frac{1}{2}} \widehat{\mathbf{R}}_{\boldsymbol{S+1}}^{-1} \widehat{\mathbf{D}}_{\boldsymbol{S+1}}^{-\frac{1}{2}} \qquad (15)$$

Where $\widehat{\mathbf{D}}_{\boldsymbol{S+1}}$ is an (*S*+1)x(*S*+1) diagonal matrix with *s*-th elements $\widehat{D}_s = 2\hat{p}_s\hat{q}_s\hat{n}_s$, and $\hat{n}_s$ is the effective sample sizes of SNP *s* (as defined in equation 11). Here, the residual variance of the joint analysis, can be estimated with the equation:

$$\hat{\sigma}_J^2 = \hat{V}_P - \hat{\mathbf{b}}_{\boldsymbol{S+1}}{}' \widehat{\mathbf{V}}_{\boldsymbol{S+1}} \widehat{\boldsymbol{\beta}}_{\boldsymbol{S+1}}$$

The vector of z-scores for the joint SNP effects for these *S+1* SNPs is given by

$$\mathbf{z}_{\boldsymbol{S+1}} = \text{diag}(\text{Var}(\widehat{\boldsymbol{\beta}}_{\boldsymbol{S+1}}))^{-\frac{1}{2}} \widehat{\boldsymbol{\beta}}_{\boldsymbol{S+1}} \qquad (16)$$

In this single ancestry COJO algorithm, in each iteration, SNPs whose joint p-value fall above certain significance threshold are removed. The above conditional and joint analysis steps are repeated until no SNP can be removed or added. In our analyses we set the significance threshold for selection as p-value < 5×10^{-8}.

1.2.3 Extending COJO analysis into multi-ancestry settings

The single-ancestry COJO algorithm outlined in the previous section can be extended into a multi-ancestry setting. By leveraging diverse LD structures from different ancestry groups, we expect to gain better resolution in tagging causal variants (shared across ancestry groups), and hence in estimating the number of independent associations.

- *Overview of the multi-ancestry COJO algorithm*

The multi-ancestry COJO algorithm assumes GWAS summary statistics of two or more ancestry groups which have been processed through quality control steps described in section 1.2.1. The multi-ancestry COJO algorithm is summarised in 7 steps and we have implemented it in the new Manc-COJO software. The software requires a predefined genome-wide significance threshold (we used 5×10^{-8}) and a pre-set collinearity threshold between SNPs (*i.e.* maximum LD r^2, we used 0.9).

Step 1: Conduct an inverse-variance meta-analysis (IVM) of GWAS summary statistics across all ancestry groups. The SNP with the smallest meta-analysis *p*-value is selected for inclusion in the model.

Step 2: SNPs above the pre-set collinearity threshold with any SNPs already included in the model in any ancestry are removed in all ancestry groups.

Step 3: Within each ancestry group, a conditional analysis is performed and the conditional effects of each SNP are calculated using Equation (13).

Step 4: An IVM of the conditional effects of each SNP is then conducted and the SNP with the smallest meta-analysis *p*-value is selected for inclusion in the model (referred to as the candidate SNP).

Step 5: Subsequently, a joint analysis is conducted within each ancestry group using Equation (14).

Step 6: An IVM is then performed to estimate the joint effects of the SNPs currently included in the model. SNPs with joint meta-analysis *p*-values exceeding the pre-specified threshold are iteratively removed in a backward selection procedure, eliminating one SNP at a time in order of decreasing significance (i.e., the SNP with the largest *p*-value is removed first), until all remaining SNPs are significant. If, during this process, the candidate SNP's joint meta-analysis *p*-value exceeds the significance threshold, the candidate SNP is rejected, the model is reverted to its state prior to the addition of this SNP, and the candidate SNP is excluded from consideration in subsequent iterations. The next most significant SNP is then evaluated, and the procedure is repeated.

Step 7: Steps 2 through 6 are repeated until no SNP can be added to or removed from the model.

- *Manc-COJO and GCTA-COJO:Fix*

We developed a pipeline, termed FIX, that enables users to retain specified variants in the COJO model by preventing their removal during the variable selection process. This facilitates the detection of ancestry-specific variants and supports functions such as the integration of wet-lab–validated evidence. Further details of the algorithm are provided in the **Supplementary Note 3**.

- *Optional R^2 parameter*

In variable selection algorithms, in addition to the approach implemented in the GCTA-COJO model, another commonly used strategy is to consider changes in the adjusted R^2 (Coefficient of determination) of model. Using summary statistics and an LD reference, the R^2 of the multiple linear regression can be approximated as:

$$\hat{R}^2 = \frac{\hat{\mathbf{b}}_S{}'\hat{\mathbf{V}}_S\hat{\boldsymbol{\beta}}_S}{\hat{V}_P}$$

where $\hat{\mathbf{b}}_S, \hat{\mathbf{V}}_S, \hat{\boldsymbol{\beta}}_S$ are as defined above. The estimated adjusted R^2 of the model can then be calculated as:

$$\hat{R}^2_{adj} = 1 - \frac{(1 - \hat{R}^2)(\hat{n} - 1)}{\hat{n} - s - 1}$$

Where s is the number of SNPs in the model, $\hat{n}$ is the effective sample size as defined in equation (11).

In the Manc-COJO algorithm, it is possible to use changes in $\hat{R}^2_{adj}$ as an *optional* selection criterion for researchers who prefer a more conservative model in regions with extremely complex LD structure. Researchers can specify thresholds that require the $\hat{R}^2_{adj}$ of the model to increase by a certain percentage during both forward and backward selection in *both* ancestry groups for a SNP to be included in the model.

For example, setting the forward-selection $\hat{R}^2_{adj}$ threshold to 0.1 means that, in each iteration, after including a SNP, the $\hat{R}^2_{adj}$ of the model must be at least 1.1 times the value from the previous iteration in both ancestry groups. Conversely, setting the backward-selection threshold to −0.1 means that, if adding a new SNP causes other SNPs to become insignificant and be dropped, then after excluding those SNPs, the $\hat{R}^2_{adj}$ of the resulting model must remain at least 0.9 times the value from the previous iteration for the new SNP to be retained. Otherwise, the algorithm reverts to the model from the last iteration and continues screening other SNPs for inclusion. By default, the $\hat{R}^2_{adj}$ thresholds for both forward and backward selection are set to −1, meaning that the selection criterion based on $\hat{R}^2_{adj}$ is not applied.

- *The benefit of multi-ancestry analyses*

By leveraging differences in LD structures across ancestry groups, Manc-COJO may enhance the ability to detect independent associations. To illustrate this, we begin with the single-ancestry analysis.

Under the infinitesimal model, and given the standardised phenotypic variance, the estimation error in GWAS for SNP *m* ($\hat{S}_m$) can be approximated by $\frac{1}{\sqrt{2\hat{p}_m\hat{q}_m\hat{n}_m}}$. In GWAS analyses, a common simplifying assumption is that the genotype matrix is fixed, even though it typically represents only a sample of the population. Consequently, we can approximate $\frac{1}{\sqrt{2\hat{p}_m\hat{q}_m\hat{n}_m}} \approx \frac{1}{\sqrt{2p_m q_m n_m}}$ (Note, this assumption again underscores the importance of selecting an appropriate LD reference, as discussed previously, and highlights the need to understand the limitations of each model arising from its underlying assumptions.).

Therefore for SNP *m*, the association z-score in GWAS is,

$$z_m = \frac{\hat{b}_m}{\hat{S}_m} = \frac{b_m + e_m}{\hat{S}_m} = e_m\sqrt{2p_m q_m n_m} + \sum_{c=1}^{C} r_{m,c}\beta_c\sqrt{2p_c q_c n_m}$$

$$= \sum_{c=1}^{C} r_{m,c}\beta_c\sqrt{2p_c q_c n_m} + \varepsilon_m \qquad (17)$$

where $\varepsilon_m = e_m\sqrt{2p_m q_m n_m}$, and it follows that $\varepsilon_m \sim \mathrm{N}(0,1)$

When there is only one causal SNP within an LD block, equation (17) can be simplified to

$$z_m = r_{m,c}\beta_c\sqrt{2p_c q_c n_m} + \varepsilon_m \qquad (18)$$

where $\beta_c\sqrt{2p_c q_c}$ is a constant across all SNPs (note $r_{m,c}$, β_c, p_c, and q_c here are population parameters). In other words, in the absence of genotype missingness—so that the sample sizes of all SNPs are **equal**—and when causal SNP effect sizes are **large**, it is relatively straightforward to identify the SNP closest to the causal SNP (*i.e.,* the SNP with the highest $|r_{m,c}|$). However, even in this idealised scenario, if per-SNP sample sizes differ due to genotype missingness, or if there are multiple tightly linked variants, the causal SNP may not correspond to the largest $|z_m|$, even when it is genotyped.

When there is more than one causal SNP in a region of correlated SNPs, it is evident from equation (4) that the most significant SNP may not be the SNP closest to causal SNPs owing to effects such as genetic masking[8] (which refers to when trait increasing alleles are present on different haplotype backgrounds). Recognising that genomic regions are likely to harbour multiple causal SNPs motivates the joint analysis approach.

In multi-ancestry meta-analysis, the z-score of SNP *i* ($z_{i,manc}$), derived from an inverse-variance meta-analysis of summary statistics from each ancestry group, can be expressed as follows:

$$z_{i,manc} = \sum_{anc}\sqrt{\frac{D_{i,anc}}{D}}\, r_{i,c,anc}\beta_c\sqrt{D_{c,anc}} + e \qquad (19)$$

Here,

- $z_{i,manc}$ is the z-score of the SNP *i* in the multi-ancestry analysis IVM

- $\mathrm{D}_{\mathrm{i,anc}} = 2p_{i,anc}q_{i,anc}n_{anc}$, where $p_{i,anc}$ is the MAF of the SNP *i* in a specific ancestry
- $D = \sum_{anc} D_{i,anc}$
- $r_{i,c,anc}$ is the LD correlation between SNP *i* and the causal SNP *c* in a specific ancestry
- β_c is the causal effect, which is assumed to be identical across ancestry groups
- Assuming the estimation error of effect sizes are independent across ancestry groups, it follows $e \sim \mathrm{N}(0,1)$

Note that in the equation (19), $p_{i,anc}$, $r_{i,c,anc}$, $q_{i,anc}$, and β_c are all population parameters. When there are no genotype missingness, $\beta_c\sqrt{D_{c,anc}}$ is a constant for an ancestry. Assuming causal variants are shared across ancestry groups, $r_{i,c,anc}$ will reach its maximum (*i.e.*, 1) in all ancestry groups when the SNP *i* is either the causal SNP or in complete LD with causal SNP in all ancestry groups. Given that LD structures differ across ancestry groups, extending COJO algorithms across multiple ancestry groups can narrow the LD window, thereby improving resolution in identifying the causal variants. However, as discussed previously, when per-SNP sample sizes vary, causal effect sizes are small, or causal SNPs are rare (i.e., low MAF, resulting in lower $D_{c,anc}$ values), the SNP selected by the COJO algorithm may not correspond to the SNP closest to the causal variant, due to the influence of the error term.

When there is more than one causal SNP in a region, equation (19) can be further extended to

$$z_{i,manc} = \sum_{anc} \sqrt{\frac{D_{i,anc}}{D}} \sum_{c=1}^{C} r_{i,c,anc}\beta_c\sqrt{D_{c,anc}} + \varepsilon \quad (20)$$

Simulations show that the multi-ancestry COJO algorithm performs better than the single ancestry analysis of the same sample size in terms of finding independent associations.

Supplementary Methods 2. Simulations

We conducted a series of simulations to compare the multi-ancestry COJO analysis with the single-ancestry COJO analysis of the same sample size with multiple criteria.

2.1 Simulation settings

2.1.1 Simulation cohort

We used real genotype data from chromosome 22 of UK Biobank participants to establish our simulation framework. From all unrelated individuals (coefficient of relationship ≤ 0.05, as calculated using GCTA's default algorithm) of inferred European ancestry (EUR; *N* ~ 340,000) and African ancestry (AFR; *N* = 6,901)—defined based on published ancestry projections[9]—we selected five cohorts. The AFR cohort (AFR1) comprises 6,901 unrelated participants of inferred AFR. EUR cohort 1 (EUR1) and cohort 2 (EUR2) each consist of 6,901 randomly selected, unrelated individuals of inferred EUR, with no overlap or relatedness between EUR1 and EUR2. We then combined EUR1 and EUR2 to form a cohort named EUR1_EUR2 (*N* = 13,802). Additionally, we

randomly selected 13,802 unrelated European participants, independent of EUR1_EUR2, for out-of-sample prediction (named EURpred), and selected 2,000 African participants for out-of-sample prediction in African ancestry (named AFRpred). The sample sizes of EUR1 and EUR2 were matched to AFR1 because previous studies have shown that combining equal-sized European and African ancestry cohorts maximises power for detecting causal associations in multi-ancestry meta-analyses[10,11].

2.1.2 Partitioning the genome

Previously, Berisa & Pickrell partitioned the genome and defined LD-independent blocks separately for African and European ancestry groups[11], assuming that LD correlations between SNPs in distinct LD-independent blocks are negligibly different from zero. In this study, we first partitioned chromosome 22 separately for the African and European cohorts (N=33 and N=23 blocks, respectively). The blocks were partially overlapping, and N=52 overlapping sections were extracted (**Supplementary Fig.1**). Within each of these 52 overlapping blocks, we calculated allele frequencies for all SNPs (using PLINK 1.9 --freq) in the AFR1, EUR1, EUR2, EURpred and AFRpred cohorts, retaining only SNPs with MAF>=0.01 across all data sets. Blocks with fewer than 1,000 SNPs remaining after this filtering were excluded, resulting in 38 blocks for use in simulation.

2.1.3 Simulation of causal SNPs

Within each of the 38 selected blocks, we randomly selected 1, 5, 10, or 20 SNPs to be causal variants. Causal effects (β_c) were simulated to be identical across ancestry groups and such that the average per-SNP heritability ($h_c^2 = 2p_cq_c\beta_c^2$) of causal SNP ranged between 0.5% and 1.5% in all cohorts. However, heritability values could differ across ancestry groups owing to varying MAFs for the same SNP. To avoid collinearity, LD r^2 between all pairs of SNPs selected to be causal were required to be ≤ 0.8 in both ancestry groups. For each combination of block and number of causal variants, we repeated the simulation 50 times, using different sets of causal variants and effect sizes in each iteration. This resulted in 7,600 scenarios, accounting for 38 blocks, 4 different numbers of causal SNPs, and 50 variations of causal SNP sets and effect sizes.

2.1.4 Simulation of phenotypes

We simulated phenotypes for individuals within each cohort as in equation (1)**.** Now,

- $\mathbf{y}$ is an *N* x 1 vector of simulated phenotypes.
- $\mathbf{X}_C$ is an *N* x *C* genotype, with entries encoded as 0, 1, or 2, corresponding to the number of alternative alleles at each SNP. Missing values in $\mathbf{X}_C$ are imputed as the mean value of genotypes of that SNP in that specific cohort.
- $\boldsymbol{\beta}_C$ is a *C* x 1 vector of simulated effect sizes as described in the previous section, and $\mathbf{e}_C$ is an *N* x 1 error vector, and each element of the vector is assumed to be drawn from a normal distribution N ~ (0, var($\mathbf{X}_C\boldsymbol{\beta}_C$)*($\frac{1-h^2}{h^2}$)), where, h^2 is approximated by $\sum_c 2p_cq_c\beta_c^2$ (β_c is *c*-th element of vector $\boldsymbol{\beta}_C$) is the total SNP-based heritability.

We simulated phenotypes within each of the six cohorts (AFR1, EUR1, EUR2, EUR1_EUR2, EURpred, AFRpred).

2.1.5 GWAS simulation, and benchmark against existing method

For each of the 7,600 scenarios, we conducted a GWAS in four cohorts (EUR1, EUR2, EUR1_EUR2 and AFR1) using PLINK 2.0 (--glm hide-covar cols=+a1freq). Subsequently, we performed an inverse-variance meta-analysis of summary statistics from EUR1 and AFR1 using custom *R* code, naming the resulting cohort EUR1_AFR1. We compared outputs across the following GWAS and model combinations: Manc-COJO with EUR1 and AFR1, GCTA-COJO with EUR1_EUR2 and GCTA-COJO using EUR1_AFR1. Default settings were applied in all scenarios for both GCTA-COJO and Manc-COJO, with in-sample LD used throughout. For the EUR1_AFR1 cohort, in-sample LD was estimated from the merged genotypes of AFR1 and EUR1 using PLINK 1.9 (--bmerge). For application of GCTA-COJO in multi-ancestral data, we also applied a EUR-only LD reference (EUR1_EUR2 in this case) in addition to the in-sample mixed-ancestry LD.

2.1.6 Model performance measurement metrics

To assess model performance, we established several criteria to evaluate Manc-COJO outputs. First, to compare Manc-COJO outputs with true effects, we recorded how many COJO-selected SNPs were simulated as causal SNPs and the proportion of COJO-selected SNPs that were causal. This criterion is justified because the primary objective of Manc-COJO is to construct a model with SNPs that have independent associations. However, in some instances, rather than selecting causal SNPs, nearby SNPs in high LD with the causal SNPs may be included in the final selected set, which still aligns with the goal of the method. To account for such scenario, we calculated two additional measures as indicators for power and false positive discovery: we first build a *CxM* LD correlation matrix, where *C* is the number of causal SNPs, and *M* is the number of COJO SNPs. For each of *M* rows, we pick the highest absolute value, and then calculate the mean of this group of highest values. This can be viewed as an indicator for power, as it measures for each causal SNP the highest LD correlation to COJO SNPs. In an idealised situation, where all causal SNPs have been correctly identified, this number should be 1. Similarly, for each of *M* COJO SNPs, we calculated the mean of the highest absolute values from each column, and subtracted the mean from 1. The resulting value provides an indicator of false-positive discoveries: it equals 0 when all COJO SNPs are causal SNPs and approaches 1 when COJO SNPs are less correlated with the causal SNPs. LD correlation matrices were calculated from both EUR1_EUR2 and AFR1 cohort.

Beyond comparisons with the ground truth, we also assessed out-of-sample prediction accuracies in independent European and African ancestry cohorts using joint SNP-effect sizes of COJO SNPs. Although the COJO algorithm is not designed to maximise out-of-sample risk prediction, comparing out-of-sample prediction using COJO selected SNPs is a criterion that can be applied in real data analysis as well as in simulation. We randomly divided the 13,800 EURpred individuals into 23 groups of 600, calculated polygenic scores for each group ($\mathbf{y}_{\text{Pred}} = \mathbf{X}_{\text{Pred}}\boldsymbol{\beta}_{\text{COJO}}$), and fitted a linear model in R using lm() to obtain the adjusted coefficient of determination (R^2_{adj}). We then computed the mean and standard deviation of these 23 regression coefficients. Similarly, we randomly divided the 2,000 AFRpred individuals into 20 groups of 100, and calculated the adjusted coefficient of determination.

2.2 Simulation sensitivity analysis

Section 2.1 outlined our simulation strategies under idealised conditions, where the ancestry composition maximises power to detect causal associations, all causal SNPs are genotyped, causal effects are identical across ancestry groups, and the LD structure in the reference panel matches that of the cohort used for GWAS. However, in practice, these assumptions are often violated. To assess the impact of such violations, we conducted a series of simulations in which one assumption was altered at a time.

We first considered a scenario where European samples constitute the majority of the cohort. All simulation settings remained identical to those described in Section 2.1, except that the EUR1 sample size was increased to 69,010. For all simulations in this scenario, in-sample LD reference panels were used. This means that the LD reference for EUR1 was also larger than in Section 2.1. As a result, the input for Manc-COJO was the GWAS summary statistics from EUR1 (N = 69,010) and AFR1 (N = 6,901). For single-ancestry GCTA-COJO, the input was GWAS from EUR1_EUR2 (N = 75,911). For multi-ancestry GCTA-COJO with ancestry-matched LD reference, the input was the meta-analysis of EUR1 and AFR1 GWAS, and the LD reference panel was the merged EUR1 and AFR1 cohort (i.e. EUR1_AFR1). We also examined an additional setting: multi-ancestry GCTA-COJO using a EUR-only LD reference panel. In this case, the input GWAS was the same meta-analysis of EUR1 and AFR1, but the LD reference was EUR1_EUR2.

In a second simulation scenario we dropped the assumption that all causal SNPs are genotyped. To achieve this, we kept all simulation settings identical to those described in Section 2.1, with the sole exception that we restricted the analysis to HapMap3 SNPs (using gcta --extract and Manc-COJO --extract).

In the third simulation scenario we dropped the assumption of the in-sample LD reference panel. To do this, we downloaded genotype data from the 1000 Genomes Project and randomly sampled two sets of unrelated individuals (N = 503) from European participants, as defined by the 1000 Genomes Project, to serve as the LD reference for EUR. Similarly, we selected N = 503 African individuals as the LD reference for AFR.

In the final simulation scenario we allowed SNP effect sizes to differ across ancestry groups, although retaining direction of association. As in previous settings, all simulation parameters matched those in Section 2.1, except that we relaxed the requiring identical effect sizes across ancestry groups. Instead, we sampled the true effect sizes independently in the two ancestry groups, while ensuring that the directions (sign) of effect were consistent.

2.3 Simulation for the FIX function

Simulations in Sections 2.1 and 2.2 assumed shared causal SNPs across ancestry groups. In this study, we developed the FIX function to account for scenarios where ancestry-specific causal

variants exist, and tested it in simulation. In our simulations, 60% of the causal SNPs in each block were shared across ancestry groups, while 20% were specific to each of the two ancestry groups. For example, in a region with 10 simulated causal SNPs, 6 were shared, 2 were EUR-specific, and 2 were AFR-specific. We simulated scenarios with either 5, 10, or 20 causal SNPs per region. The effect sizes of shared causal SNPs were identical across ancestry groups. Phenotype simulation followed the same procedure as described in Section 2.1. In total, we conducted simulations in 38 blocks, across 3 different numbers of causal SNPs, with 50 replicates per combination, resulting in 5,700 simulated scenarios.

Supplementary Methods 3. Real data analysis

We evaluated the performance of Manc-COJO in real data, using ancestry-specific GWAS summary statistics of European and African ancestry groups for four lipid traits chosen because of their large sample sizes for both European and African ancestry groups. Global Lipids Genetics Consortium (GLGC) GWAS summary statistics (which excluded UK Biobank) were available for high-density lipoprotein cholesterol (HDL), low-density lipoprotein cholesterol (LDL), total cholesterol (TC), and log-transformed triglyceride levels (logTG)[12].

3.1 Data quality control

We used GWAS summary statistics that excluded participants from the UK Biobank. Only common SNPs (MAF $\geq$ 0.01) present in both ancestry groups and bi-allelic SNPs were retained for analysis. Further filtering was conducted to retain only those SNPs that were genotyped in the UK Biobank across both ancestry groups and had an MAF $\geq$ 0.01 among unrelated African participants (N = 6,901). Following quality control, the median per-SNP sample sizes and the number of SNPs included for each trait were as follows: HDL (N_{AFR} = 90,804, N_{EUR} = 881,859, M = 6,514,392 SNPs); LDL (N_{AFR} = 87,759, N_{EUR} = 837,509, M = 6,511,658 SNPs); TC (N_{AFR} = 92,554, N_{EUR} = 924,769, M = 6,514,274 SNPs); logTG (N_{AFR} = 89,467, N_{EUR} = 858,015, M = 6,514,129 SNPs).

3.2 Data input

For each trait, the inputs to the Manc-COJO algorithm consisted of two separate GWAS summary statistics (one for each ancestry group) and two corresponding LD reference panels: a randomly sampled set of 69,010 unrelated European individuals and 6,901 unrelated African individuals from the UK Biobank. Default parameters were applied: a window size of 10Mb (assuming SNPs more than 10Mb apart to be independent), a collinearity threshold of 0.9, and a significance threshold of 5×10^{-8}, consistent with the original GCTA-COJO protocol.

We benchmarked Manc-COJO against GCTA-COJO applied to both multi-ancestry and single-ancestry datasets. For the multi-ancestry analysis, the input was the inverse-variance meta-analysis of the aforementioned European and African GWAS summary statistics from GLGC. The LD reference was either a merged cohort of 69,010 European and 6,901 African ancestry individuals, or a merged cohort of 69,010 European and 6,901 European ancestry individuals (using PLINK's –bmerge function). For the single-ancestry analysis, new GWAS were conducted on 90,000 unrelated European UK Biobank samples for each of the lipid traits. Covariate adjustment followed the original GIANT and GLGC protocols: for lipid traits, covariates were age, age-squared, batch, sex, assessment centre, and the first 20 PCs. For individuals on cholesterol-lowering medications (Field IDs p6153_i0 and p6177_i0), LDL (LDL_{adj} = LDL/0.7) and TC (TC_{adj} = TC/0.8) levels were adjusted. Analyses were sex-stratified: residuals were computed separately for males and females and then pooled. Subsequently, we conducted an inverse-variance meta-analysis of new GWAS summary statistics derived from UKB European

participants and aforementioned EUR-specific GWAS, excluding UK Biobank data, from the GLGC. The LD reference for single-ancestry GCTA-COJO was a merged cohort of 69,010 and 6,901 unrelated European UK Biobank individuals. In general, we ensured that the input GWAS summary statistics involved approximately equal sample sizes and identical SNP sets, and LD reference panel sizes are comparable across methods for fair comparison (**Supplementary Table 1**).

Given that samples from GLGC were heavily skewed towards European ancestry (approximately 10:1 ratio of European to African participants after excluding UK Biobank samples), we conducted a down-sampling analysis to examine whether closer ancestry balance (approximately 1:1) would enhance detection of independent associations. Four approaches were again evaluated: (1) Manc-COJO applied to multi-ancestry data; (2) GCTA-COJO applied to multi-ancestry data using mixed-ancestry LD references; (3) GCTA-COJO applied to multi-ancestry data using EUR-only LD references; and (4) GCTA-COJO applied to single-ancestry data. For Manc-COJO, we used AFR-specific GWAS from GLGC and newly generated EUR-specific GWAS from UK Biobank (N ~ 90,000 per lipid trait). For multi-ancestry GCTA-COJO, input GWAS were derived from inverse-variance meta-analysis of the AFR- and EUR-specific GWAS used for Manc-COJO. LD reference panels consisted of either 6,901 unrelated EUR individuals plus 6,901 unrelated AFR individuals or two independent sets of 6,901 unrelated EUR individuals. For single-ancestry GCTA-COJO, GWAS summary statistics were generated from ~180,000 unrelated EUR individuals per lipid trait in UK Biobank using the same analytical pipeline as in the full-scale analyses, and the corresponding LD reference comprised 13,802 unrelated EUR individuals excluding individuals contributing to the GWAS (**Supplementary Table 1**).

3.3 Out-of-sample prediction in UK Biobank

To obtain the weights for polygenic prediction and ensure a fair comparison across all methods, we first identified the list of SNPs selected by the Manc-COJO and GCTA-COJO models. These SNPs were then jointly **re-estimated** using the GCTA --cojo-joint command, based on the summary statistics and LD reference panels specified in **Supplementary Table 1** (for full-sample analyses) and **Supplementary Table 2** (for down-sampled analyses). After obtaining the joint effect sizes for each model, polygenic scores were computed using the PLINK --score command.

To evaluate out-of-sample prediction performance in European cohorts, we included 47,066 unrelated European participants for lipid traits, none of whom were included in previous GWAS analyses. Prediction into African cohorts was assessed using 3,000 unrelated UKB African participants for lipid traits. Residuals for all traits were computed following the same covariate adjustments described for the GWAS analyses.

We randomly divided 47,066 unrelated UKB EUR participants into 50 groups, and 3,000 UKB AFR participants into 20 groups, with one group having more individuals if the sample size was not evenly divisible. Within each group, a simple linear regression (residual ~ PGS + error) was performed, and the out-of-sample R^2 was calculated as the coefficient of determination. The

final R^2 estimate was averaged across groups for each trait and ancestry, and the standard deviation of the estimate was computed.

To compare genomic regions where the Manc-COJO and GCTA-COJO models identified different numbers of SNPs, we partitioned the genome into LD-independent blocks based on a previously published framework[13]. Note that the LD blocks used here differ from those in the simulation study, as we opted for a more conservative definition to reduce the likelihood of false positive claims in real data analysis. For each individual LD block, the out-of-sample prediction accuracy of each COJO model (Manc-COJO or GCTA-COJO) was estimated by first removing the COJO-selected SNPs within that block, recalculating the joint effect sizes using the remaining SNPs, and then computing the difference in prediction accuracy between the full model and the reduced model.

3.4 Out-of-sample prediction in All of Us

We reassessed out-of-sample predictive performance using data from the All of Us cohort. Because SNPs in All of Us are aligned to the GRCh38 reference genome, we first generated a conversion table linking SNP rsIDs and genomic coordinates between the UK Biobank (GRCh37) and All of Us (GRCh38) datasets using the UCSC LiftOver tool. A small subset of variants (~0.3 million of the 8.5 million SNPs with MAF ≥ 0.01 in the UK Biobank) could not be successfully mapped from GRCh37 to GRCh38. We then extracted genotype data from the ACAF call set, which includes SNPs with MAF ≥ 0.01 or an allele count ≥ 100 in any sub-population ("gs://fc-aou-datasets-controlled/v8/wgs/short_read/snpindel/acaf_threshold/plink_bed"). Genotypes were obtained for all AFR and EUR individuals, as defined in "gs://fc-aou-datasets-controlled/v8/wgs/short_read/snpindel/aux/ancestry/ancestry_preds.tsv". We excluded related individuals and those who did not pass quality control, as indicated in "gs://fc-aou-datasets-controlled/v8/wgs/short_read/snpindel/aux/relatedness/" and "gs://fc-aou-datasets-controlled/v8/wgs/short_read/snpindel/aux/qc/flagged_samples.tsv".

To ensure a fair comparison, we repeated the Manc-COJO and GCTA-COJO analyses using the approximately 1-million-sample version of the summary statistics (~0.9 million EUR plus 0.09 million AFR or EUR) as described above, but retained only SNPs that were present in both the UK Biobank and All of Us cohorts at the start of the analysis.

For lipid traits, we extracted phenotypes defined as "Cholesterol [Mass/volume] in Serum or Plasma," "Cholesterol in HDL [Mass/volume] in Serum or Plasma," "Cholesterol in LDL [Mass/volume] in Serum or Plasma," "Cholesterol in LDL [Mass/volume] in Serum or Plasma by calculation," and "Triglyceride [Mass/volume] in Serum or Plasma." We retained only measurements reported in units of "milligram per deciliter" or "milligram per deciliter calculated." For individuals with multiple measurements of the same trait, we kept the earliest measurement. For participants taking lipid-lowering medications (Atorvastatin, Ezetimibe, Fluvastatin, Lovastatin, Pravastatin, Rosuvastatin, Simvastatin), LDL and total cholesterol levels were adjusted as in the GLGC study (LDL_adj = LDL/0.7; TC_adj = TC/0.8), provided the participant was actively

taking the medication at the time of measurement, determined by comparing medication exposure start and end dates with the lipid measurement date.

We then adjusted each trait for age, sex, and the first 16 PCs, and calculated scaled residuals separately within each ancestry group. After generating residuals, we divided EUR (~80,000 per trait) and AFR (~25,000 per trait) samples into groups of 1,000 and applied the same protocol used in the UK Biobank to compute PGS.

Supplementary Notes 1. Simulation sensitivity analysis

In practice, model assumptions are often violated. In addition to the scenarios described in the main text, we conducted analyses restricted to HapMap3 SNPs to mimic situations in which causal SNPs are not genotyped, simulations in which effect sizes differed between ancestry groups, and simulations in which LD matrices were not calculated from the GWAS samples themselves. As expected, the performance of all models declined under these non-idealised conditions. Nonetheless, Manc-COJO consistently exhibited greater robustness than the GCTA-COJO-based methods.

Causal SNPs are not always genotyped in practice. To simulate this scenario, we retained the simulation settings used under the idealised condition but restricted the analysis to HapMap3 SNPs only. All three methods performed reasonably well in European ancestry, with the resulting models identifying SNPs at similar LD distances from the causal variants. However, the Manc-COJO → 6.9K AFR+6.9K EUR (HM3 SNPs only) method yielded fewer false positive findings. Specifically, the false positive indicator (calculated using LD from European ancestry samples) was lower for Manc-COJO compared to GCTA-COJO → 6.9K AFR_6.9K EUR (HM3 SNPs only) : 0.03 vs 0.04, 0.09 vs 0.11, 0.11 vs 0.14, and 0.13 vs 0.16 for regions with 1, 5, 10, and 20 causal SNPs, respectively. In contrast, when calculating false positive and power metrics using LD from African ancestry samples, GCTA-COJO—as expected—exhibited reduced power and increased false positive rates. While the GCTA-COJO → 6.9K AFR_6.9K EUR (HM3 SNPs only) demonstrated comparable power to Manc-COJO, it consistently resulted in higher numbers in false positive indicator: 0.18 vs 0.17, 0.25 vs 0.21, 0.27 vs 0.23, and 0.30 vs 0.25 for 1, 5, 10, and 20 causal SNPs in a region, respectively (**Supplementary Fig.3**).

In our modelling framework, we initially assumed that the true effect sizes of causal SNPs were identical across ancestry groups. To assess robustness under non-idealised scenarios, we relaxed this assumption in simulation, while keeping all other parameters consistent with the idealised scenarios. In all simulated conditions, the effect sizes of the causal SNPs varied across ancestry groups; however, the direction of association remained consistent. Under these settings, Manc-COJO → 6.9K AFR+6.9K EUR consistently outperformed both GCTA-COJO → 6.9K AFR_6.9K EUR and GCTA-COJO → 13.8K EUR by identifying a greater number of causal SNPs, demonstrating higher statistical power, and yielding fewer false positive discoveries (**Supplementary Fig.4**).

Finally, we simulated scenarios with imperfect LD references. The simulation settings were identical to those of the idealised scenarios, with the sole exception that, instead of using in-sample LD, the LD references were derived from the 1000 Genomes Project. For this, we randomly selected N = 503 unrelated European individuals and N = 503 unrelated African individuals as a reference sample (see **Supplementary Methods**). Generally, the trends remain consistent with the idealised scenario: Manc-COJO → 6.9K AFR+6.9K EUR/503 AFR + 503 EUR 1000G LD or GCTA-COJO → 6.9K AFR_6.9K EUR/503 AFR_503 EUR 1000G LD identify a greater number of causal SNPs and exhibit superior statistical power compared to GCTA-COJO→ 13.8K EUR/503 EUR 1000G LD, while among the two multi-ancestry approaches, Manc-COJO generates fewer false-positive discoveries. However, compared to the scenario using in-sample LD, the

ability to detect independent associations diminishes across all methods. For example, the median number of causal SNPs identified by Manc-COJO decreases from 6 to 5 and from 11 to 8 when there are 10 and 20 causal SNPs in a region, respectively. Concurrently, the median false-positive indicator rises sharply, increasing from 0 to 0.01, 0.01 to 0.09, and 0.03 to 0.19 when there are 5, 10, and 20 causal SNPs in a region, respectively (**Supplementary Fig.5**). This underscores the benefit of using in-sample LD.

Supplementary Notes 2. Down-sampled analysis

In the main text, we down-sampled the EUR GWAS to match the sample size of the AFR GWAS to show that using a EUR-only LD reference can inflate false-positive signals when the ancestral proportion in the meta-analysis of GWAS summary statistics approaches 1:1. In addition to this analysis, we repeated the down-sampling procedure shown in **Extended Data Fig.4** to evaluate whether achieving a more balanced ancestry composition (~1:1) in GWAS sample sizes enhances the detection of independent associations (**Supplementary Table 2**). Given the reduced GWAS sample sizes the R^2 values in all analyses were naturally lower. In EUR cohorts, the out-of-sample R^2 (and standard deviation) for Manc-COJO → 0.09M AFR+0.09M EUR/6.9K AFR+6.9K EUR LD models were lower than that of GCTA-COJO → 0.18M EUR/13.8K EUR LD models: 0.126 (0.022) vs 0.134 (0.020) for HDL, 0.139 (0.022) vs 0.144 (0.024) for LDL, 0.076 (0.013) vs 0.083 (0.015) for logTG, and 0.125 (0.022) vs 0.130 (0.020) for TC. Nonetheless, in African cohorts, Manc-COJO models demonstrated consistently better cross-ancestry portability, achieving higher out-of-sample R^2 values than the GCTA-COJO models: 0.090 (0.042) vs 0.073 (0.032) for HDL, 0.142 (0.047) vs 0.128 (0.045) for LDL, 0.046 (0.024) vs 0.012 (0.0080) for logTG, and 0.121 (0.044) vs 0.116 (0.047) for TC. Notably, as the ancestry balance approached parity, the difference in model size between the methods became more pronounced: Manc-COJO consistently yielded more compact models with fewer selected SNPs—139 vs 182 for HDL, 118 vs 141 for LDL, 97 vs 144 for logTG, and 139 vs 157 for TC (**Supplementary Fig.6**). Overall, although GCTA-COJO models showed higher predictive accuracy in EUR cohorts under the down-sampled setting—because the EUR GWAS summary statistics used by Manc-COJO were now much less powered than those used by GCTA-COJO—Manc-COJO models still retained reasonable accuracy in European cohorts and outperformed GCTA-COJO in African cohorts while including up to 30% fewer SNPs. These results suggest that a more balanced ancestry composition may improve Manc-COJO's ability to detect independent associations, but we currently lack sufficiently large non-EUR datasets—particularly AFR datasets, which have the shortest LD among all ancestry groups—to directly demonstrate this. This highlights the importance of increasing sample sizes for non-European ancestry groups.

Supplementary Notes 3. The Manc-COJO:FIX function

Our software also introduces a new feature that allows users to fix selected variants in the model, ensuring that these SNPs are retained during subsequent variable selection procedures. This model is implemented in both multi-ancestry and single-ancestry algorithms.

3.1 When to use Manc-COJO:FIX

Manc-COJO:FIX can be applied in a variety of analytical settings. As described in the main text, variants identified in the primary Manc-COJO model—based on the assumption of shared causal variants across ancestry groups—can be fixed to facilitate the identification of ancestry-specific signals. Alternatively, because the standard Manc-COJO model operates on SNPs shared across all cohorts, it may exclude variants present only in a subset of ancestry groups or cohorts. In such cases, SNPs identified in an analysis including all ancestry groups can be fixed, and subsequent analyses can be performed within any selected subsets of ancestry-defined cohorts.

In addition, Manc-COJO:FIX allows users to fix variants that have been validated experimentally (i.e., through wet-lab studies), which can aid in disentangling independently associated variants in regions with complex LD structure.

3.2 Manc-COJO:FIX algorithms

The Manc-COJO:FIX algorithm follows the general framework of the Manc-COJO algorithm described in the Methods section, but incorporates modified collinearity and *p*-value checking procedures. In addition, it ensures that user-specified FIXed SNPs are retained and not removed during subsequent variable selection steps.

In Manc-COJO:FIX, the model is partitioned into two components: (i) FIXed SNPs (provided by the user), and (ii) COJO SNPs (variants newly selected into the model).

Collinearity check

During iterative selection, the model evaluates pairwise LD correlations among SNPs to prevent the inclusion of collinear variants. Three thresholds are defined, each corresponding to a different block of the LD correlation matrix:
--fix-col: collinearity threshold among FIXed SNPs (default: 0.9)
--fix-cojo-col: collinearity threshold between FIXed SNPs and newly selected COJO SNPs (default: 0.9)
--cojo-collinear: collinearity threshold among newly selected COJO SNPs (default: 0.9)

A candidate SNP is rejected if it violates any of the applicable collinearity thresholds. Although a threshold of 0.9 performs well across many traits and settings, evidence from height analyses (Yengo *et al.*, Supplementary Note 1[14]) indicates that alternative thresholds can reduce false-positive findings while preserving power in certain contexts. Because no single threshold is optimal for all traits, users are encouraged to evaluate a range of thresholds tailored to each trait.

***p*-value check**
During iterative selection, when a candidate SNP (i.e., the SNP with the smallest conditional *p*-value below the predefined threshold *--cojo-p*, default value: 5×10^{-8}) is proposed for inclusion, the following steps are applied sequentially:

1. If adding the candidate SNP causes the maximum joint *p*-value among the FIXed SNPs to exceed *--fix-p* (which may differ from *--cojo-p*; The default value is 1), the candidate is rejected. The next most significant SNP is then considered, and the procedure is repeated.

2. If condition (1) is not triggered but adding the candidate causes the maximum joint *p*-value among the COJO SNPs to exceed *--cojo-p*, the candidate is provisionally accepted, and backward selection among COJO SNPs is initiated (i.e, FIXed SNPs are not included in this backward selection step).

3. During backward selection, COJO SNPs are removed one at a time, starting with the SNP with the largest *p*-value, until both conditions (1) and (2) are satisfied. If all COJO SNPs are removed and either condition remains unsatisfied, or if the candidate SNP no longer meets the *--cojo-p* threshold, the model reverts to its previous state prior to adding the candidate. The candidate SNP is then excluded from further consideration in subsequent iterations.

4. This process is repeated until no additional SNPs can be added or removed.

***Choice of p*-value threshold**
In the FIX function, *--cojo-p* specifies the *p*-value threshold for selecting new SNPs into the model; we therefore recommend a more stringent threshold (5×10^{-8}). The *--fix-p* parameter defines the threshold for FIXed SNPs, and its choice depends on the characteristics of these variants. For example, when there is strong prior evidence that FIXed SNPs are causal and they fail to meet a stringent threshold due to limited power, a more lenient threshold may be appropriate (*--fix-p* = 1 implies no filtering based on *p*-values). In contrast, when fixed SNPs are included without external support and for exploratory purposes, we recommend a more stringent threshold (as applied in the real data analyses, where *--fix-p* = 5×10^{-8}).

For completeness, we also evaluated *--fix-p* = 1 in the real data analyses. In the AFR cohort, relative to Manc-COJO, Manc-COJO:FIX resulted in changes in prediction R^2 (with corresponding increases in model size) of 0.0062 (15), 0.0007 (22), 0.0034 (11), and −0.0057 (18) for HDL, LDL, logTG, and TC, respectively. In the EUR cohort, the corresponding changes were 0.0032 (160), 0.0023 (77), 0.0032 (131), and 0.0034 (102). Despite substantial increases in model size, the gains in predictive performance were minimal or even negative, further indicating that most phenotypic variation is well explained by models assuming shared causal variants across ancestry groups, regardless of parameter settings.

3.3 Interpretation of the Manc-COJO:FIX function outcomes in the context of ancestry specific associations

In simulations, applying Manc-COJO:FIX within each ancestry group—by fixing variants identified in the Manc-COJO model—can recover a subset of independent association signals when true ancestry-specific effects are present. However, in real data analyses, we find that most phenotypic variation is well explained by models assuming shared causal variants across ancestry groups, regardless of parameter settings.

Across traits, additional variants identified by Manc-COJO:FIX generally explain only a small proportion of phenotypic variance. Moreover, we cannot exclude the possibility that the additional association signals detected by the FIX function reflect differences in statistical power, given that current GWAS are predominantly based on European-ancestry samples, rather than true ancestry-specific causal variants. For example, a causal variant that is shared across ancestry groups may be underpowered for detection in AFR due to smaller sample sizes, leading to imprecise effect estimates. As a result, the variant may be non-significant in AFR, significant in EUR, but fail to reach significance in the meta-analysis due to the added noise from AFR. In such cases, the variant may later be detected in EUR-specific analyses and incorrectly interpreted as EUR-specific despite being shared. This issue is further compounded by the steeper LD decay in AFR populations. At current SNP probe densities, the signal from true causal variants tends to attenuate more rapidly at nearby markers than in EUR, thereby further reducing power in AFR.

Therefore, we recommend **against** using Manc-COJO:FIX as an integrated part of routine analyses to identify ancestry specific variants, unless cohorts across ancestry groups are sufficiently well powered and SNP density is high. This encourages users to make deliberate analytical choices. Underpowered analyses may still use this approach for exploratory purposes, but the results should be interpreted with a clear understanding of their limitations. We believe the proposed method can identify ancestry specific variants if they exist, and that as sample sizes (especially non-EUR population) increase, the results will clarify their presence or absence.

Supplementary Notes 4. Algorithmic improvements over original COJO

Supplementary Note 4.1 More robust outputs

Although our software is designed for multi-ancestry analyses, it can also be used to perform single-ancestry COJO analyses as implemented in GCTA. We refer to our implementation of GCTA-COJO within our software as **Sanc-COJO**, which incorporates two refinements to the original GCTA-COJO algorithm in GCTA while remaining otherwise identical.

First, during early iterations, GCTA stores *p*-values that fall outside the representable range of the C++ double type (1.7×10^{-308} to 1.7×10^{308}) as zero. When several *p*-values are truncated in this way, multiple SNPs become indistinguishably significant, potentially causing a suboptimal SNP to be selected for the joint model. Our implementation instead prioritises the SNP with the largest absolute z-score, which is mathematically equivalent but avoids numerical underflow.

Across the four lipid traits and 22 chromosomes (88 analyses), nine trait–chromosome combinations were affected by this refinement (**Supplementary Table 3**), with the majority of selected SNPs remaining identical to GCTA outputs. Incorporating this refinement results in slightly fewer selected SNPs and marginally improved prediction accuracy for single-ancestry COJO (**Supplementary Table 4**), as expected given that the algorithm is now guaranteed to retain the truly most significant SNP at each iteration.

Second, in rare scenarios involving complex LD patterns—such as applying GCTA-COJO analyses to multi-ancestral data in the main text, where European and African genotype matrices are merged for pseudo–single-ancestry analyses—the final iteration may remove SNPs due to collinearity without adding new ones. In GCTA, this can create index inconsistencies and unstable *.cma.cojo* outputs. This issue does not occur for the *--cojo-cond* option, where the SNP set is fixed by the user, and we did not observe it in standard single-ancestry datasets such as the ~1 million European cohort analysed in this study.

We performed extensive checks and confirmed that our software produces numerically identical results to GCTA (for *.cma.cojo*, *.jma.cojo*, and *.ldr.cojo* outputs) for all analyses in this study, except for scenarios listed in **Supplementary Table 3,** where the implemented refinements improved upon the original GCTA software.

Supplementary Note 4.2 Improved computation efficiency

The main speedup in our implementation comes from recoding genotype values into compact binary form and operating on them with native bitwise instructions. Instead of storing SNP genotypes as arrays of 0, 1, and 2 and performing arithmetic on *int* or *double* types, we pack them into machine-width integers such as *uint64_t*. This enables each CPU instruction to process many SNPs simultaneously using bitwise AND, OR, NOT, and population-count operations. Because these instructions execute as low-level hardware operations, they avoid the overhead of scalar arithmetic and floating-point pipelines. As a result, genotype storage and computation become more memory-efficient, cache-aligned, and effectively executed at

hardware speed, yielding substantial throughput gains for large-scale COJO analyses. Other improvements include reusing intermediate results across correlation matrices, integrating optimised C++ libraries to accelerate input data parsing, and other engineering refinements. Full implementation details are available on our open-source GitHub repository to ensure reproducibility.

Supplementary Note 4.3 Dealing with data missingness

In real data analyses, missingness can arise in both the genotypic data used to compute LD references—due to factors such as genotyping errors or imputation failure—and in the GWAS summary statistics themselves, where genotype missingness within a cohort or differences in the SNP sets reported across cohorts in a meta-analysis can lead to substantial variation in sample size across SNPs. Out-of-sample prediction introduces an additional layer of complexity, as genotype missingness may also occur in the target cohort used for prediction.

In LD calculations, different software handle missing genotypes differently. Tools such as GCTA impute missing genotypes by replacing them with the mean genotype value, whereas software like PLINK exclude all pairs containing missing values when computing pairwise correlations. Both approaches have limitations. Mean imputation reduces the genotypic variance of the SNP, causing the variance to deviate from HWE. In contrast, removing pairs with missing data leads to less precise estimates of genetic correlation, particularly when the LD-reference sample size is not large enough.

In addressing missingness in GWAS summary statistics, GCTA introduces an additional adjustment when computing joint effects (as in equation 4) and conditional effects (as in equation 7). Although this adjustment is not explicitly presented in the original GCTA-COJO paper, GCTA effectively rescales the pairwise LD correlations according to the per-SNP effective sample sizes in GWAS summary statistics. Specifically, the correlation between SNP *i* and SNP *j* is multiplied by $\min(\hat{n}_i, \hat{n}_j)/\hat{n}_j$, and the correlation between SNP *j* and SNP *i* by $\min(\hat{n}_i, \hat{n}_j)/\hat{n}_i$ where $\hat{n}_i$ is the per-SNP effective sample sizes of SNP *i* defined in equation (11).

When calculating polygenic scores, mean imputation is commonly used to handle missing genotype data.

In most COJO- or fine-mapping–based workflows, the calculation of joint effects for polygenic prediction is performed immediately after variable selection and typically uses the same strategy for handling missingness. Interestingly, our results show that variable selection and the estimation of joint effects are in fact two distinct steps that should be treated differently. In our study, we evaluated three approaches for variable selection—(i) excluding individuals with missing genotypes on a per–SNP-pair basis during pairwise LD computation, (ii) imputing missing values when calculating pairwise LD, and (iii) imputing missing values and adjusting for missingness in GWAS summary statistics using the GCTA-based method described above. We applied the same three strategies when computing joint effects for polygenic prediction, yielding nine possible combinations of variable-selection and joint-effect procedures.

For variable selection, the optimal strategy varied across traits. However, in out-of-sample prediction, the approach that imputes missing values and adjusts GWAS summary statistics for missingness consistently outperformed the other two strategies—an unsurprising result given how missingness is handled in the target sample (**Supplementary Table 5**). For simplicity, in the main text we report results using the “remove missing values” strategy for variable selection and the “impute missing values plus adjust for missingness” strategy for estimating joint effects.

Our software implements all three missingness-handling strategies, allowing users to choose the method most appropriate for them. Meanwhile, the input GWAS summary statistics can be adjusted to mitigate missingness. For example, researchers may down-weight effect sizes at loci with substantial missingness by correcting marginal GWAS effects, scaling them by $\sqrt{INFO}$[15], where INFO can be approximated as the ratio of the per-SNP sample size to the total sample size. In addition, specialised tools such as SLALOM[16] and DENTIST[17] can be used to identify loci exhibiting suspicious heterogeneity across cohorts, which has been shown to improve downstream analyses such as fine-mapping.

Supplementary Notes 5. Insight into Manc-COJO vs GCTA-COJO output

To better understand differences between Manc-COJO and GCTA-COJO in a real-world setting, we examined and compared their model outputs from multiple perspectives.

In the main text, we present the distribution of out-of-sample prediction R^2 across validation subgroups as a descriptive comparison of Manc-COJO and GCTA-COJO. These results suggest that Manc-COJO generally retains predictive accuracy in individuals of European ancestry and generally improves prediction in individuals of African ancestry. We therefore formally compared the models using the R package r2redux[18]. Because the two polygenic scores derived from Manc-COJO and GCTA-COJO output were evaluated in the same validation individuals, their R^2 estimates are statistically dependent. r2redux accounts for this dependence by estimating the sampling variance of each r2redux, their covariance, and the variance of their difference.

When predicting in EUR individuals, Manc-COJO → 0.09M AFR + 0.9M EUR/6.9K AFR + 69K EUR LD and GCTA-COJO → 0.99M EUR/75.9K EUR LD did not differ significantly after two-sided tests with Bonferroni correction across all four lipid traits: HDL ($\Delta R^2 = -0.0026$, unadjusted 95% CI −0.0048 to −0.00047, Bonferroni-adjusted P = 0.20), LDL ($\Delta R^2 = -0.00068$, unadjusted 95% CI −0.0025 to 0.0011, adjusted P = 1.0), TC ($\Delta R^2 = -0.00030$, unadjusted 95% CI −0.0023 to 0.0017, adjusted P = 1.0), and logTG ($\Delta R^2 = 0.0015$, unadjusted 95% CI −0.00063 to 0.0035, adjusted P = 1.0). In AFR individuals, however, Manc-COJO showed numerically higher R^2 values for all four traits. The improvements for LDL ($\Delta R^2 = 0.019$, unadjusted 95% CI 0.0068 to 0.031, Bonferroni-adjusted P = 0.029) and logTG ($\Delta R^2 = 0.027$, unadjusted 95% CI 0.015 to 0.039, adjusted $P = 8.4 \times 10^{-5}$) remained significant after Bonferroni correction.

When comparing the prediction accuracy of Manc-COJO with GCTA-COJO → 0.09M AFR + 0.9M EUR/6.9K AFR + 69K EUR LD in EUR samples, Manc-COJO achieved numerically higher prediction accuracy for all four traits, with significant improvements after Bonferroni correction for LDL, TC, and logTG. The Manc-minus-GCTA differences in R^2 were HDL: $\Delta R^2 = 0.0013$ (unadjusted 95% CI −0.0010 to 0.0035, Bonferroni-adjusted P = 1.0); LDL: $\Delta R^2 = 0.0058$ (unadjusted 95% CI 0.0039 to 0.0077, adjusted $P = 3.8 \times 10^{-8}$); TC: $\Delta R^2 = 0.0074$ (unadjusted 95% CI 0.0052 to 0.0097, adjusted $P = 1.4 \times 10^{-9}$); and logTG: $\Delta R^2 = 0.0041$ (unadjusted 95% CI 0.0019 to 0.0062, adjusted $P = 2.1 \times 10^{-3}$). In AFR individuals, Manc-COJO also showed numerically higher R^2 values for LDL ($\Delta R^2 = 0.012$, unadjusted 95% CI 0.00086 to 0.023, Bonferroni-adjusted P = 0.41), TC ($\Delta R^2 = 0.0039$, unadjusted 95% CI −0.0089 to 0.017, adjusted P = 1.0), and logTG ($\Delta R^2 = 0.0076$, unadjusted 95% CI −0.0026 to 0.018, adjusted P = 1.0), although none of these differences remained significant after Bonferroni correction. For HDL, Manc-COJO was slightly lower than GCTA-COJO, most likely because of sampling variation ($\Delta R^2 = -0.00025$, unadjusted 95% CI −0.0099 to 0.0094, adjusted P = 1.0).

When comparing Manc-COJO with GCTA-COJO → 0.09M AFR + 0.9M EUR/75K EUR LD, Manc-COJO consistently achieved higher prediction accuracy in EUR samples, with significantly better performance for TC and logTG after Bonferroni correction. The Manc-minus-GCTA differences in R^2 were 0.00070 for HDL (unadjusted 95% CI −0.00086 to 0.0023, adjusted P = 1.0), 0.0017 for

LDL (95% CI 0.00032 to 0.0030, adjusted P = 0.18), 0.0028 for TC (95% CI 0.0013 to 0.0043, adjusted P = 3.1×10^{-3}), and 0.0027 for logTG (95% CI 0.0012 to 0.0042, adjusted P = 5.9×10^{-3}). In AFR individuals, none of the differences between the Manc-COJO and GCTA-COJO models reached statistical significance: HDL ($\Delta R^2 = -0.0054$, 95% CI −0.012 to 0.0015, adjusted P = 1.0), LDL ($\Delta R^2 = -0.000018$, 95% CI −0.0085 to 0.0085, adjusted P = 1.0), TC ($\Delta R^2 = -0.0016$, 95% CI −0.011 to 0.0082, adjusted P = 1.0), and logTG ($\Delta R^2 = 0.010$, 95% CI 0.0027 to 0.018, adjusted P = 0.10).

Overall, Manc-COJO models were generally smaller than the corresponding GCTA-COJO models in both single- and multi-ancestry settings, while achieving equivalent or better prediction performance across all scenarios evaluated. In order to understand the factors contributing to differences in model size between the Manc-COJO and GCTA-COJO models, we partitioned SNPs identified in the full-sample analysis into LD-independent blocks as defined previously[13] (**Supplementary Methods**). We then compared the number of SNPs included in the Manc-COJO model with those in the single-ancestry GCTA-COJO model within each LD-independent block (**Supplementary Fig.7**).

For most LD blocks (specifically, 64.2% of blocks for HDL, 66.2% for LDL, 61.7% for TC, and 68.7% for logTG), the Manc-COJO and GCTA-COJO models identified an identical number of independent associations. As the example shown in the main text, within these blocks, although the number of independent associations is the same, Manc-COJO identifies independently associated SNPs that are more closely correlated (in terms of LD) with the functional variants than those flagged by GCTA-COJO. For example, for rs2277862 in TC, Manc-COJO selected rs12625762, which has signed LD correlations (r) of 0.994 (EUR) and 0.942 (AFR) with the functional SNP. In contrast, GCTA-COJO selected rs1010759, with corresponding LD correlations of 0.992 (EUR) and 0.918 (AFR). A similar pattern occurred for rs10889356 in TC: Manc-COJO selected rs995000, whereas GCTA-COJO selected rs626787. The LD correlations with the functional SNP were 0.962 (EUR) and 0.960 (AFR) for the Manc-COJO–selected SNP, compared with 0.955 (EUR) and –0.476 (AFR) for the GCTA-COJO–selected SNP. For rs10872142 in LDL, Manc-COJO selected rs13210143, which shows extremely high LD with the causal SNP in both EUR (0.998) and AFR (1.000). In contrast, GCTA-COJO selected rs1556857, whose LD correlations were 0.996 (EUR) and 0.899 (AFR) (**Fig. 5**).

We observed similar patterns in out-of-sample prediction accuracy. For LD-independent blocks containing rs2277862 in TC, Manc-COJO SNPs explained 2.7×10^{-4} more phenotypic variance in AFR than GCTA-COJO SNPs, but 3.6×10^{-6} less variance in EUR. For LD-independent blocks containing rs10889356 in TC, the corresponding differences were 3.1×10^{-4} more in AFR and 3.6×10^{-5} less in EUR. For blocks containing rs10872142 in LDL, Manc-COJO SNPs explained 3.7×10^{-4} and 1.4×10^{-5} more phenotypic variance in AFR and EUR, respectively. Because LD correlations between Manc- or GCTA-COJO SNPs and the causal SNPs are uniformly high and similar in EUR, it is not unexpected that GCTA-COJO SNPs can, due to random error, show slightly higher out-of-sample R^2 in this population. In AFR, however, Manc-COJO SNPs consistently yield higher out-of-sample R^2, and the magnitude of the difference is larger (on the order of 10^{-4}). Although the out-of-sample prediction sample sizes are too small to support statistically

significant differences (Bonferroni-adjusted $P > 0.05$ in all scenarios), the observed patterns are notably consistent with expectations.

The main reason the Manc-COJO model is generally smaller than the GCTA-COJO model is that in many LD blocks the GCTA-COJO model selects one or occasionally two SNPs, whereas the Manc-COJO model does not select any SNPs in those blocks. Specifically, this pattern accounts for 69 SNPs across 62 blocks for HDL, 50 SNPs across 46 blocks for LDL, 48 SNPs across 43 blocks for TC, and 70 SNPs across 64 blocks for logTG (**Supplementary Fig.7**). These SNPs identified by the GCTA-COJO model make up approximately 6–10% of all SNPs in the model and are not rare variants, with mean minor allele frequencies of 0.27 for HDL, 0.28 for LDL, 0.26 for TC, and 0.28 for logTG in the European population. However, they collectively explain only a small proportion of phenotypic variance. The sum of out-of-sample prediction accuracies for these SNPs when predicting into European cohorts was 0.0012 for HDL, 0.0018 for LDL, 0.0014 for TC, and 0.0025 for logTG. Prediction accuracies in African cohorts were even lower, at 0.00043, −0.0010, 0.0011, and −0.0021 for HDL, LDL, TC, and logTG, respectively. The fact that this large set of SNPs consists of common variants yet explains only a negligible proportion of phenotypic variance suggests that at least some of the independent associations tagged by the GCTA-COJO model in these blocks are more likely to represent false-positive signals rather than causal SNPs with small effects.

In general, in real data applications, althought the differences between Manc-COJO and GCTA-COJO models in terms of variance explained by their COJO SNPs were not statistically significant in all scenarios, which likely reflects power (sample sizes of non-European GWAS are currently much smaller than for European GWAS, 10-fold for the lipid traits studied here, and non-European sample sizes for out-of-sample prediction are also limited). Nonetheless, the pattern of results, replicated across the traits and databases studied is consistent with expectations from well-powered simulations. The use of Manc-COJO applied to cross-ancestry GWAS meta-analyses will become increasingly relevant as samples sizes for non-European GWAS increase over time. Moreover, our results provide insights likely relevant to other post-GWAS methods that rely on GWAS summary statistics and LD references.

Supplementary Notes 6. Meta-analysis, mega-analysis, and admixed population

Owing to data-sharing constraints, GWAS summary statistics stratified by ancestry remain the most widely accessible data resource. This limitation motivated the development of a meta-analysis–based framework to identify independent associations in multi-ancestry settings.

In idealised scenarios where individual-level genotype data are available across all ancestry groups, a mega-analysis can be performed as an alternative to a meta-analysis–based approach. In such settings, genotype data are pooled across cohorts, and GWAS is conducted on the combined sample with appropriate covariate adjustment for ancestry. Under idealised conditions—where in-sample LD is available and sample sizes are sufficiently large—mega-analysis may offer greater statistical power, as shown in previous research[19]. However, when in-sample LD is unavailable or conditions deviate from these ideal assumptions, simulations as shown in our study indicate that mega-analysis can be highly susceptible to inflated false-positive rates (**Fig.2; Fig.3**).

A similar issue arises for admixed populations, where prior work has shown that standard GWAS of admixed cohorts can surpass the power of equally sized multi-ancestry mega-analyses with homogeneous within-ancestry structure[20]. However, COJO analysis in admixed populations also relies heavily on having a perfectly matched LD reference.

Given the constraints on data sharing and the practical challenges of pooling individual-level data, we therefore focus on meta-analysis–based strategies in the main text. Nonetheless, our software can be readily adapted to perform COJO analyses in a mega-analysis framework that combines multiple homologous cohorts across diverse ancestral backgrounds, as well as in admixed populations. The main caveat is that, as shown in Equation (8), when underlying subpopulations differ in allele frequencies, the HWE assumption may be violated, and the genotype variance can no longer be approximated by $2pq$. In these situations, the allele-frequency column in the input file should be replaced with the effective allele frequency, which can be obtained by solving the quadratic equation that equates $2pq$ with the observed genotype variance in the sample.

To accommodate mega-analysis and analyses in admixed populations, we developed a function (*--var-from-LD*) that directly calculates genotypic variance from the LD reference sample, rather than relying on the $2pq$ approximation or manual calculation. In this mode, the allele frequency column in the GWAS summary statistics is not used.

However, the purpose of *--var-from-LD* is to enable COJO analyses using GWAS summary statistics and an LD reference derived from the same set of samples, approximating the scenario in which individual-level genotype data are available. **The method does not correct for deficiencies in the upstream analytical pipeline used to generate the summary statistics or LD reference**. For example, SNP correlations may reflect not only LD but also population stratification; if factors such as in-sample PCs are not properly accounted for in the GWAS, or if

the LD reference does not closely match the genomic ancestry proportions of the study sample, the resulting outputs will be unreliable. Users should therefore exercise **extra caution** when applying the *--var-from-LD* option and carefully consider the assumptions underlying their analysis.

Supplementary Notes 7. Decision on cohort inclusion in the analysis

In multi-ancestry analyses, a common question is whether including more ancestry groups necessarily improves performance, and whether ancestry groups with substantially smaller sample sizes should be incorporated. In this section, we first simulate an idealised setting in which all ancestry groups are well powered to illustrate the potential benefits. We then down-sample one ancestry to evaluate the impact of including a group with a smaller sample size.

7.1 Extension of Manc-COJO to multiple ancestry groups

The simulation settings were identical to those outlined in Supplementary Note 2.1. We retained the AFR1 cohort (comprising 6,901 unrelated participants of inferred AFR), the EUR1 and EUR2 cohorts (each consisting of 6,901 randomly selected, unrelated individuals of inferred EUR), and additionally sampled 6,901 unrelated EUR individuals to form the EUR3 cohort, with no overlap or relatedness with EUR1 and EUR2. We also randomly sampled 6,901 unrelated SAS individuals from UKB.

We compared four scenarios: (i) applying GCTA-COJO to EUR1_EUR2_EUR3 (combining EUR1, EUR2 and EUR3 into a single cohort of 20,703 individuals); (ii) applying Manc-COJO to EUR1_EUR2 + SAS (as two separate inputs); (iii) applying Manc-COJO to EUR1_EUR2 + AFR (as two separate inputs); and (iv) applying Manc-COJO to EUR1 + SAS + AFR (as three separate inputs). Evaluation metrics were consistent with those used previously, with the addition of power and false positive indicators calculated based on LD in SAS.

Across all evaluation metrics, multi-ancestry analyses showed higher power and fewer false positive findings than single-ancestry analyses of the same sample size. Among analyses involving two ancestry groups, EUR1_EUR2 + AFR was better powered and yielded fewer false positives than EUR1_EUR2 + SAS, including for metrics evaluated using LD in SAS. This is consistent with the shorter LD observed in AFR compared with SAS. Notably, analyses incorporating all three ancestry groups were consistently more highly powered and produced fewer false positives than those including only two (**Supplementary Fig.8**).

Overall, our simulations demonstrate the benefit of including more ancestry groups in the analysis. Although LD may be longer in certain ancestry groups, for example SAS compared with AFR, differences in LD structure across ancestral groups can still help to break down LD and improve the identification of independent associations. This is in addition to the potential to detect structural variation or variants that are more common in specific ancestry groups. Therefore, international efforts are needed to increase data availability across all ancestry groups.

7.2 Inclusion of a smaller cohort

In practice, there remains a lack of data from non-EUR populations, raising the question of whether inclusion of ancestry groups with relatively small sample sizes is warranted. To address this, we conducted a series of simulations based on equation (19) in Supplementary Note 1.2.3. Briefly, one of 38 LD blocks was randomly selected, and within this block (BP: 25,662,928–

26,469,735 on chr22; GRCh37), individual SNPs were iteratively designated as the causal variant, assumed to be shared across ancestry groups. Allele frequencies and LD correlation matrices were derived from the EUR1_EUR2, AFR and SAS cohorts described above.

We first considered a two-ancestry setting. The EUR sample size was fixed at 0.5M, while the AFR sample size was incrementally increased from 0 to 0.1M in steps of 5,000 individuals. The true causal effect size was fixed at 0.1 across ancestry groups. For each configuration, we generated 10,000 realisations of z-score distributions across SNPs using equation (19). We then quantified performance by calculating the LD between the SNP with the largest absolute z-score and the true causal SNP, and summarising this as the mean LD r^2 across simulations. Distributions of mean LD r^2 values were evaluated across all SNPs when treated as causal variants. As shown in **Supplementary Fig.9a,b**, inclusion of an additional ancestry improves the ability of COJO to resolve independent signals; however, gains are limited when the added sample size is small (for example, ~5,000 individuals). This limitation is more evident in the EUR–SAS combination (**Supplementary Fig.9b**), consistent with the longer-range LD structure in EUR and SAS relative to AFR.

We next extended the framework to three ancestry groups. Fixing EUR at 0.5M and AFR at 0.1M, we varied the SAS sample size from 0 to 0.5M. Conversely, fixing EUR at 0.5M and SAS at 0.1M, we varied the AFR sample size over the same range in increments of 50,000. In both scenarios (**Supplementary Fig. 10a,b**), increasing the sample size of the third ancestry improved the ability of COJO to identify independent associations, with selected signals showing stronger correspondence to the true causal SNP across populations. This effect was more pronounced when increasing AFR sample size, consistent with its shorter LD structure (**Supplementary Fig. 10b**).

Notably, these simulations assume a single causal variant per locus and identical effect sizes across ancestry groups. In addition, the Manc-COJO framework analyses only SNPs shared across all included populations. As shown in **Supplementary Fig. 3**, reduced SNP density adversely affects model performance. Given differences in variant calling pipelines and genotyping platforms across studies, SNP coverage may vary substantially between cohorts. Consequently, inclusion of an ancestry with both limited sample size and low overlap in SNPs may be detrimental to model performance, as the loss of power due to reduced SNP density may outweigh the modest gains from additional ancestry.

Overall, the extent to which multi-ancestry data improve COJO performance depends on several factors, including the number of causal variants per locus, genotype density and the underlying genetic architecture across populations. A formal framework to estimate required sample sizes to break down the LD is beyond the scope of this study, and we aim to provide such a resource in future work. At present, we recommend considering the inclusion of additional ancestry groups with smaller sample sizes when: (1) SNPs available for analysis show strong overlap with other datasets; (2) data collection and analytical pipelines are harmonised, as in large international consortia; and (3) high-quality individual-level genotype data are available to construct reliable LD reference panels. The original GCTA-COJO paper showed that

an LD reference panel with more than 5,000 individuals generally provides reliable LD estimates. Ideally, in-sample LD should be used (i.e., the GWAS cohort itself has a sample size of at least 5,000). Alternatively, LD estimates may be derived from an external cohort drawn from the same ancestral population so that the LD structure is well-matched to that of the GWAS cohort; in this case, the GWAS sample may be smaller, although—as illustrated in the figure—the gains are expected to be limited when the GWAS sample size falls below 5,000.

Conversely, if any of these conditions are not met, then including small cohorts may introduce more noise than benefit. In such cases, empirical evaluation (e.g., model size and out-of-sample predictive performance) should guide decisions about whether to include a given cohort.

Supplementary Notes 8. COJO and fine-mapping tools

Simulations show that, in an idealised scenario where all causal variants are genotyped, no pairs of causal variants exhibit LD above 0.8, and studies are well-powered, GCTA-COJO identifies 40% of causal variants for less polygenic traits (5 causal SNPs in a region) and 16% for more polygenic traits (20 causal SNPs in a region). Manc-COJO increases this to 60% and 40%, respectively, by leveraging differing LD structures across ancestry groups. However, the assumptions (common to all fine-mapping methods) that all causal variants are genotyped (or imputed) and studies are well-powered are rarely met in practice. With whole-genome sequence data, where nearly all genetic variants are genotyped (though gaps remain, especially with short-read sequencing[7]), many variants are in complete or near-complete LD, necessitating indefinitely large sample sizes to completely resolve LD.

We compared Manc-COJO with MultiSuSiE[21] and MESuSiE[22] using the same simulation data in this study, in which all causal variants are shared across ancestry groups and no ancestry-specific effects are present.

For both MultiSuSiE and MESuSiE, we constructed credible sets using a cumulative posterior inclusion probability (PIP) coverage threshold of 0.95 and a minimum purity threshold of 0.5, consistent with the default and commonly adopted settings in the SuSiE framework. For MESuSiE, we set the ancestry weight vector to restrict the model to infer only shared (common) SNP effects. For both methods, the maximum number of independent signals was set equal to the true number of simulated causal variants (1, 5, 10, or 20).

The results are summarised in **Supplementary Table 6a**. Each cell reports four values corresponding to simulations with 1, 5, 10, and 20 causal variants, respectively. Each value is averaged over 1,900 replicates (38 LD blocks × 50 scenarios), using the same data as in Fig.3. We define the **mean number of causal variants recovered** as the average count of true causal variants successfully identified. For MultiSuSiE and MESuSiE, a causal variant is considered recovered if it appears in at least one credible set; for Manc-COJO, if it is included among the selected SNPs. We also define **mean proportion** as the number of recovered true causal variants divided by the total number of unique SNPs nominated by the method (the union of all credible set members for fine-mapping methods, or all selected SNPs for Manc-COJO). A higher mean number of causal variants recovered indicates a more powerful model of picking causal variants, while a higher mean proportion indicates that a greater fraction of nominated SNPs are true causal variants and thus a lower false positive rate.

As shown in the table, MultiSuSiE and MESuSiE achieve higher power than Manc-COJO across all simulation settings, recovering a larger number of true causal variants. However, this comes at the cost of lower precision: for example, with 10 simulated causal variants, MultiSuSiE and MESuSiE nominate on average ~30 unique SNPs per replicate to recover ~9 true causal variants (proportion ≈ 0.29), whereas Manc-COJO selects ~9 SNPs to recover ~6 true causal variants (proportion ≈ 0.64). Notably, the true number of independent signals for the SuSiE-based method is unknown in practice, and users must specify this parameter a priori. As shown in

Supplementary Table 6b, lower values reduce the size of credible sets but also decrease power, whereas higher values may slightly increase power but substantially inflate the model size (i.e. reducing the mean proportion).

This contrast is expected and reflects a fundamental difference in the objectives of the two approaches. Fine-mapping methods such as MultiSuSiE and MESuSiE jointly model all SNPs within a region under a Bayesian framework, constructing credible sets that quantify uncertainty over which variants are causal. By design, these sets may include multiple correlated variants to achieve the targeted coverage probability. In contrast, COJO applies an iterative stepwise conditional analysis, selecting at each step the most significant SNP conditional on those already included, and terminating when no additional SNPs meet the significance threshold. This procedure typically yields a parsimonious set of approximately independent variants, prioritising the identification of independent association signals rather than precise localisation of causal SNPs; in practice, tagging a variant in linkage disequilibrium with the true causal SNP is often sufficient. To further illustrate this distinction, we randomly selected a simulated scenario and present the differing outputs and underlying pipeline designs (**Extended Data Fig. 6**).

Supplementary Notes 9. Stability of the Manc-COJO selection algorithm

A commonly raised question regarding the Manc-COJO algorithm concerns its stability. The COJO framework is deterministic, in contrast to Bayesian approaches that rely on stochastic procedures such as MCMC sampling. Moreover, COJO employs a greedy selection algorithm, meaning that the order of SNP screening does not influence the final results. Consequently, identical outputs are obtained given the same input data and parameter settings.

However, differences in input data can affect the results. For example, increases in sample size within one ancestry—due to the availability of additional data—may alter the outcome, particularly for SNPs near the significance threshold. Because Manc-COJO is deterministic and selects SNPs based on the smallest p-values, small changes in the data can lead to different lead SNPs being chosen. In such cases, a nearby SNP may be selected to represent the same underlying independent association.

Another factor influencing the number of independent associations identified is the choice of input parameters, especially the p-value threshold. Different thresholds reflect distinct analytical priorities: more stringent thresholds reduce false positives and favor the identification of robust secondary associations, but at the cost of reduced statistical power. In contrast, less stringent thresholds increase power but allow a higher false-positive rate.

For example, when we re-ran Manc-COJO on four lipid traits using varying *p*-value thresholds, we observed that relaxing the threshold improved out-of-sample prediction accuracy in EUR populations, as expected given the increased number of SNPs in the model (**Supplementary Table 7**). However, despite identifying more independent associations, prediction accuracy in AFR populations declined when the threshold was lowered to 5×10^{-7} or below. This suggests that many of the additional associations identified at more permissive thresholds may not represent true signals.

In our view, less stringent thresholds may be advantageous for downstream applications such as improving predictive performance, although Bayesian methods (e.g., SBayesRC) are generally better suited for this purpose. Conversely, when the objective is to identify secondary independent associations with higher confidence, more stringent thresholds are preferable.

Supplementary Notes 10. Choice of R^2 parameter

In the Manc-COJO algorithm, it is possible to use changes in $\hat{R}^2_{adj}$ as an *optional* selection criterion for researchers who prefer a more conservative model in regions with extremely complex LD structure. Users can specify thresholds that require the $\hat{R}^2_{adj}$ of the model to increase by a certain percentage during both forward and backward selection in *both* ancestry groups for a SNP to be included in the model as described in the **Supplementary Method** section.

To evaluate the impact of applying different $\hat{R}^2_{adj}$thresholds, we tested two scenarios in both simulation and real-data analyses: (1) $\hat{R}^2_{adj}$ in both forward and backward selection was set to 0. In this setting, for a SNP to be included in the model, the $\hat{R}^2_{adj}$ of the new model after forward and backward selections must be higher than that of the previous model in both ancestry groups. (2) $\hat{R}^2_{adj}$ in forward selection was set to 0, and in backward selection set to −1. In this scenario, we imposed a threshold only for forward selection, requiring an increase in $\hat{R}^2_{adj}$ to add a SNP, while allowing any decrease in $\hat{R}^2_{adj}$ during backward selection. We performed the simulation with a EUR:AFR ratio of 10:1 to match the ancestry proportions used in the real-data analysis.

In general, applying a more stringent $\hat{R}^2_{adj}$ threshold reduces the false positive inclusion of non-causal SNPs in the model, but at the cost of reduced power to include causal SNPs, especially when the number of causal SNPs increases (**Supplementary Fig.11**).

In real data analysis, applying an $\hat{R}^2_{adj}$ threshold of 0 in both forward and backward selection substantially reduced model size compared with the default setting that imposes no $\hat{R}^2_{adj}$ restriction. Model sizes decreased from 831 to 761 for HDL, 554 to 510 for LDL, 655 to 586 for logTG, and 700 to 670 for TC. Introducing an $\hat{R}^2_{adj}$ threshold generally led to a slight reduction in out-of-sample prediction accuracy in EUR; however, for HDL and LDL we observed a small increase in AFR despite the smaller model sizes, and the available sample size did not allow us to determine the significance of this pattern (**Supplementary Table 8**).

Overall, although the default setting without an $\hat{R}^2_{adj}$ threshold performs well in most scenarios, adding such a threshold may help reduce false-positive signals in regions with highly complex LD structures, as illustrated in HDL and LDL. The optimal choice may therefore depend on the genetic architecture of the trait under study.

Supplementary Tables and Figures

Variable Selection				
**Scenarios**	**Methods**	**Input GWAS for EUR**	**Input GWAS for AFR**	**LD reference**
**Multi-ancestry Analysis**	Manc-COJO → 0.09M AFR+0.9M EUR/ 6.9K AFR+69K EUR LD	N ~ 0.9 million EUR GWAS from GLGC	N ~ 0.09 million AFR GWAS from GLGC	69,010 UKB EUR participants and 6,901 UKB AFR participants (separately)
	GCTA-COJO → 0.09M AFR_0.9M EUR/ 6.9K AFR_69K EUR LD			69,010 UKB EUR participants and 6,901 UKB AFR participants (Merged into a single cohort)
	GCTA-COJO → 0.09M AFR_0.9M EUR/ 75.9K EUR LD			75,911 UKB EUR participants (As a single cohort)
**Single-ancestry Analysis**	GCTA-COJO → 0.99M EUR/ 75.9K EUR LD	N ~ 0.9 million EUR GWAS from GLGC plus N ~ 0.09 million EUR GWAS from UKB	N/A	75,911 UKB EUR participants (As a single cohort)
**Generating polygenic prediction weights by jointly fitting selected variables with --cojo-joint**				
**EUR weight**	Output from all COJO models	N ~ 0.9 million EUR GWAS from GLGC plus N ~ 0.09 million EUR GWAS from UKB	N/A	75,911 UKB EUR participants
**AFR weight**	Output from all COJO models	N/A	N ~ 0.09 million AFR GWAS from GLGC	6,901 UKB AFR participants

Supplementary Table 1. Summary of full sample analyses. The top table summarises the GWAS summary-statistic inputs and LD reference panels used by each method for variable selection in the full-sample analyses (total sample size ~1 million). The bottom table shows that, after identifying an independent set of associated SNPs, polygenic prediction weights are derived by jointly fitting the selected variants using the –cojo-joint function on the same dataset across methods to ensure a fair comparison.

Model-fitting				
**Scenarios**	**Methods**	**Input GWAS for EUR**	**Input GWAS for AFR**	**LD reference**
**Multi-ancestry Analysis**	Manc-COJO → 0.09M AFR+0.09M EUR/ 6.9K AFR+6.9K EUR LD	N ~ 90,000 EUR GWAS from UKB	N ~ 90,000 AFR GWAS from GLGC	6,901 UKB EUR participants and 6,901 UKB AFR participants (separately)
	GCTA-COJO → 0.09M AFR_0.09M EUR/ 6.9K AFR_6.9K EUR LD			6,901 UKB EUR participants and 6,901 UKB AFR participants (Merged into a single cohort)
	GCTA-COJO → 0.09M AFR_0.09M EUR/ 13.8K EUR LD			13,802 UKB EUR participants (As a single cohort)
**Single-ancestry Analysis**	GCTA-COJO → 0.18M EUR/ 13.8K EUR LD	N ~ 180,000 EUR GWAS from UKB	N/A	13,802 UKB EUR participants (As a single cohort)
**Generating weight for polygenic prediction**				
**EUR weight**	Output from all COJO models	N ~ 180,000 EUR GWAS from UKB	N/A	13,802 UKB EUR participants (As a single cohort)
**AFR weight**	Output from all COJO models	N/A	N ~ 90,000 AFR GWAS from GLGC	6,901 UKB AFR participants

Supplementary Table 2. Summary of down-sample analyses. This table summarizes the GWAS inputs and LD reference panels used for each method, as in Supplementary Table 1, but for the down-sampled setting in which the ancestry proportions are approximately 1:1 (total sample size ~0.2 million).

Trait, Chr	SNP number (Sanc-COJO)	SNP number (GCTA-COJO)	Common SNPs
**HDL, Chr 15**	37	38	31
**HDL, Chr 16**	50	58	43
**LDL, Chr2**	54	54	53
**LDL, Chr19**	75	77	67
**logTG, Chr2**	55	55	54
**logTG, Chr8**	48	46	42
**TC, Chr1**	64	64	63
**TC, Chr2**	67	68	65
**TC, Chr19**	74	76	69

Supplementary Table 3. Comparison of the number of SNPs identified by different models in single-ancestry analyses. This table summarises the number of SNPs identified using either single ancestry COJO as implemented in GCTA or our refined version of GCTA-COJO (i.e. Sanc-COJO). We analysed four lipid traits using 1 million EUR samples and a 75,911-sample EUR LD reference panel. For most trait–chromosome combinations, the number of independently associated SNPs identified is identical between the two approaches, except for the specific scenarios listed in the table.

Trait	GCTA-COJO	Sanc-COJO
**HDL**	903, 0.077±0.044, 0.170±0.021	894, 0.077±0.046, 0.170±0.022
**LDL**	581, 0.124±0.039, 0.159±0.022	579, 0.129±0.044, 0.159±0.023
**logTG**	705, 0.027±0.013, 0.108±0.017	707, 0.028±0.014, 0.108±0.017
**TC**	718, 0.107±0.040, 0.145±0.020	715, 0.110±0.044, 0.146±0.020

Supplementary Table 4. Comparison between GCTA-COJO and Sanc-COJO. This table compares the original COJO implementation in GCTA with our updated software. The inputs are the same as those used in Supplementary Table 3. For each cell, the values shown from left to right are: (i) the total number of SNPs in the model, (ii) prediction accuracy in AFR (with s.d.), and (iii) prediction accuracy in EUR (with s.d.). Despite identifying a smaller number of SNPs, our refined GCTA-COJO algorithm achieves equal or higher prediction accuracy in both ancestry groups.

Method (Selection_Prediction)	HDL	LDL	logTG	TC
**GCTA_GCTA**	826<br>0.1675±0.0219<br>0.0836±0.0404	545<br>0.1575±0.0207<br>0.1432±0.0478	659<br>0.1071±0.0184<br>0.0490±0.0246	705<br>0.1406±0.0196<br>0.1139±0.0400
**imputeNA_GCTA**	847<br>0.1675±0.0218<br>0.0831±0.0402	567<br>0.1587±0.0205<br>0.1447±0.0496	684<br>0.1096±0.0183<br>0.0565±0.0298	720<br>0.1450±0.0195<br>0.1150±0.0430
**removeNA_GCTA**	831<br>0.1684±0.0228<br>0.0838±0.0403	554<br>0.1585±0.0212<br>0.1373±0.0456	655<br>0.1089±0.0181<br>0.0534±0.0283	700<br>0.1447±0.0204<br>0.1158±0.0437
**GCTA_imputeNA**	826<br>0.1658±0.0220<br>0.0841±0.0407	545<br>0.1541±0.0205<br>0.1412±0.0468	659<br>0.1000±0.0181<br>0.0482±0.0258	705<br>0.1320±0.0186<br>0.1104±0.0402
**imputeNA_imputeNA**	847<br>0.1638±0.0222<br>0.0835±0.0418	567<br>0.1532±0.0199<br>0.1439±0.0509	684<br>0.0982±0.0172<br>0.0559±0.0298	720<br>0.1353±0.0186<br>0.1097±0.0435
**removeNA_imputeNA**	831<br>0.1669±0.0233<br>0.0839±0.0432	554<br>0.1557±0.0211<br>0.1358±0.0453	655<br>0.1043±0.0179<br>0.0523±0.0283	700<br>0.1358±0.0194<br>0.1158±0.0453
**GCTA_removeNA**	826<br>0.1628±0.0219<br>0.0841±0.0403	545<br>0.1518±0.0204<br>0.1406±0.0452	658<br>0.0796±0.0172<br>0.0455±0.0256	704<br>0.1286±0.0185<br>0.1045±0.0378
**imputeNA_removeNA**	847<br>0.1439±0.0220<br>0.0829±0.0420	567<br>0.1441±0.0195<br>0.1419±0.0493	684<br>0.0742±0.0147<br>0.0548±0.0306	720<br>0.1263±0.0186<br>0.0739±0.0369
**removeNA_removeNA**	831<br>0.1616±0.0237<br>0.0833±0.0434	554<br>0.1502±0.0210<br>0.1352±0.0434	655<br>0.0880±0.0155<br>0.0519±0.0285	700<br>0.1279±0.0192<br>0.1137±0.0447

Supplementary Table 5. Comparison of variable-selection and joint-effect estimation strategies in Manc-COJO. In this study, we evaluated three approaches for variable selection that differ in how they calculate pairwise LD in the presence of missing genotype data: (i) removeNA, which excludes individuals with missing genotypes on a per–SNP-pair basis; (ii) imputeNA, which imputes missing genotypes when computing pairwise LD; and (iii) GCTA, which imputes missing values and additionally adjusts for missingness in GWAS summary statistics as described in Supplementary Note 3. We applied the same three strategies when estimating joint effects for polygenic prediction, resulting in nine combinations of variable-selection and joint-effect estimation methods. In each method label, the part before the underscore indicates the strategy used for variable selection, and the part after the underscore specifies the method used for joint-effect estimation in out-of-sample prediction. Each cell reports three quantities: the model size (top row), the prediction accuracy (± s.d.) in EUR (middle row), and the corresponding accuracy in AFR (bottom row). Data inputs are identical to those used in Fig.3.

(a)

Method	Mean number of causal variants recovered	Mean Proportion
**MultiSuSiE**	0.986 / 4.711 / 8.981 / 17.010	0.574 / 0.331 / 0.286 / 0.287
**MESuSiE**	0.986 / 4.628 / 8.655 / 15.706	0.575 / 0.330 / 0.285 / 0.278
**Manc-COJO**	0.693 / 3.224 / 5.912 / 10.590	0.693 / 0.667 / 0.642 / 0.638

(b)

Number of causal SNPs simulated	Method	Number of causal SNPs specified as input	Mean number of causal variants recovered	Mean proportion	Method	Number of causal SNPs specified as input	Mean number of causal variants recovered	Mean proportion
**5**	Multi-SuSiE	3	2.69	0.39	ME-SuSiE	3	2.70	0.39
		4	3.65	0.36		4	3.67	0.35
		5	4.71	0.33		5	4.63	0.33
		6	4.73	0.33		6	4.63	0.33
		7	4.74	0.33		7	4.63	0.33
**10**		6	5.28	0.32		6	5.28	0.32
		8	7.20	0.31		8	7.18	0.30
		10	8.98	0.29		10	8.66	0.28
		12	9.10	0.28		12	8.67	0.28
		14	9.08	0.28		14	8.67	0.28
**20**		12	10.16	0.33		12	10.19	0.31
		16	13.98	0.31		16	13.75	0.29
		20	17.01	0.29		20	15.71	0.28
		24	17.26	0.28		24	15.73	0.28
		28	17.19	0.29		28	15.73	0.28

Supplementary Table 6. Comparison of Manc-COJO, MultiSuSiE, and MESuSiE under simulated common causal variants. (a) Mean number of true causal variants recovered and mean proportion (fraction of nominated SNPs that are true causal variants) are reported in this table for 1, 5, 10, and 20 simulated causal variants (four values per cell, separated by "/"), averaged across 1,900 replicates (38 genomic blocks × 50 simulation scenarios). (b) As the SuSiE-based method requires specification of the number of causal variants, we varied this parameter to assess its impact on model performance.

p-value threshold	HDL	LDL	logTG	TC
**5x10^{-5}**	2299<br>0.1820±0.0195<br>0.0635±0.0276	1422<br>0.1638±0.0214<br>0.1235±0.0377	1914<br>0.1199±0.0195<br>0.0413±0.0204	1660<br>0.1507±0.0200<br>0.1034±0.0383
**5x10^{-6}**	1503<br>0.1782±0.0208<br>0.0736±0.0300	962<br>0.1634±0.0217<br>0.1307±0.0453	1208<br>0.1166±0.0187<br>0.0457±0.0227	1110<br>0.1509±0.0196<br>0.1126±0.0361
**5x10^{-7}**	1082<br>0.1721±0.0207<br>0.0794±0.0405	699<br>0.1609±0.0216<br>0.1357±0.0411	855<br>0.1120±0.0187<br>0.0495±0.0252	856<br>0.1483±0.0198<br>0.1155±0.0399
**5x10^{-8}**	831<br>0.1684±0.0228<br>0.0838±0.0403	554<br>0.1584±0.0212<br>0.1373±0.0457	655<br>0.1089±0.0181<br>0.0534±0.0283	700<br>0.1444±0.0202<br>0.1172±0.0436
**5x10^{-9}**	652<br>0.1630±0.0225<br>0.0807±0.0426	453<br>0.1559±0.0208<br>0.1399±0.0456	540<br>0.1059±0.0183<br>0.0477±0.0237	584<br>0.1425±0.0202<br>0.1241±0.0470

Supplementary Table 7. Sensitivity of Manc-COJO results to the choice of *p*-value threshold. This table summarises the outputs of the Manc-COJO model under different *p* value thresholds, using the same input data as in Fig. 3. Each cell reports three quantities: model size (i.e., number of SNPs selected bi the COJO model, top row), prediction accuracy (± s.d.) in the EUR cohort (middle row), and prediction accuracy in the AFR cohort (bottom row). The highest prediction accuracy for each trait is highlighted in red.

Trait	Manc-COJO (R^2_{Adj} threshold - Forward selection: -1; Backward selection: -1)	Manc-COJO (R^2_{Adj} threshold - Forward selection: 0; Backward selection: -1)	Manc-COJO (R^2_{Adj} threshold - Forward selection: 0; Backward selection: 0)
**HDL**	831	759	761
**LDL**	554	511	510
**logTG**	655	587	586
**TC**	700	654	670

Size of models (i.e., number of COJO-SNPs):

Prediction R^2 (and s.d.) in EUR:

Trait	Manc-COJO (R^2_{Adj} threshold - Forward selection: -1; Backward selection: -1)	Manc-COJO (R^2_{Adj} threshold - Forward selection: 0; Backward selection: -1)	Manc-COJO (R^2_{Adj} threshold - Forward selection: 0; Backward selection: 0)
**HDL**	0.168 (0.023)	0.165 (0.023)	0.165 (0.023)
**LDL**	0.158 (0.021)	0.156 (0.021)	0.156 (0.021)
**logTG**	0.109 (0.018)	0.107 (0.017)	0.107 (0.017)
**TC**	0.145 (0.020)	0.142 (0.021)	0.143 (0.021)

Prediction R^2 (and s.d.) in AFR:

Trait	Manc-COJO (R^2_{Adj} threshold - Forward selection: -1; Backward selection: -1)	Manc-COJO (R^2_{Adj} threshold - Forward selection: 0; Backward selection: -1)	Manc-COJO (R^2_{Adj} threshold - Forward selection: 0; Backward selection: 0)
**HDL**	0.084 (0.040)	0.086 (0.046)	0.087 (0.046)
**LDL**	0.137 (0.046)	0.140 (0.048)	0.140 (0.049)
**logTG**	0.053 (0.028)	0.051 (0.031)	0.046 (0.029)
**TC**	0.116 (0.044)	0.114 (0.046)	0.114 (0.044)

Supplementary Table 8. Comparison of Manc-COJO performance using different adjusted R^2 thresholds in forward and backward selections. This table summarizes the model sizes and prediction accuracies in European and African cohorts obtained under different adjusted R^2 threshold settings in the Manc-COJO models. The data inputs are identical to those used in Fig.3. For the lipid traits, using an adjusted R^2 threshold of either –1 or 0 during backward selection does not affect the results when the forward-selection threshold is set to 0.

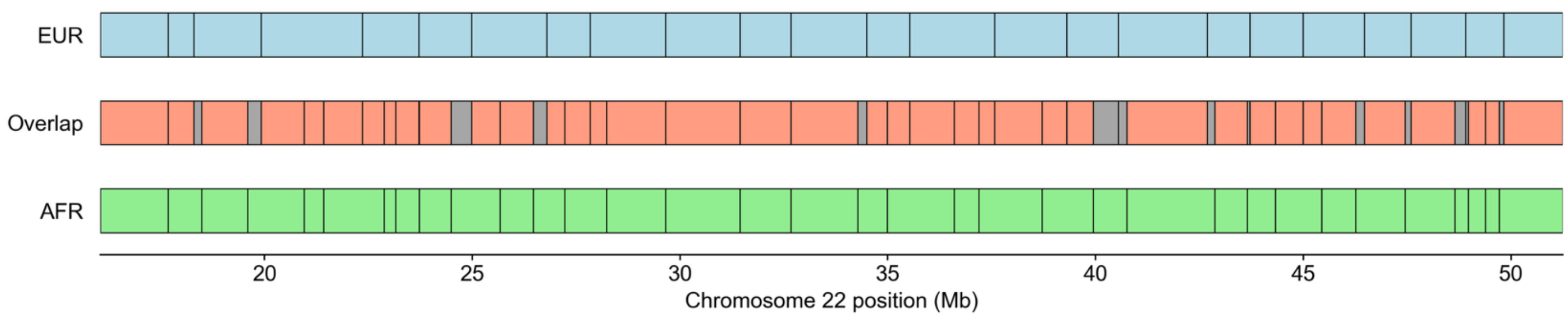

Supplementary Fig.1. Partitioning of chromosome 22 for simulation. In our study, Chromosome 22 was partitioned separately for the African and European cohorts, resulting in 33 and 23 blocks, respectively. These blocks were partially overlapping, and a total of 52 overlapping sections were extracted (middle row of the figure). Within each of these 52 overlapping regions, blocks containing fewer than 1,000 SNPs after quality control were excluded (shown in grey in the middle row), leaving 38 blocks (shown in red) for use in the simulation.

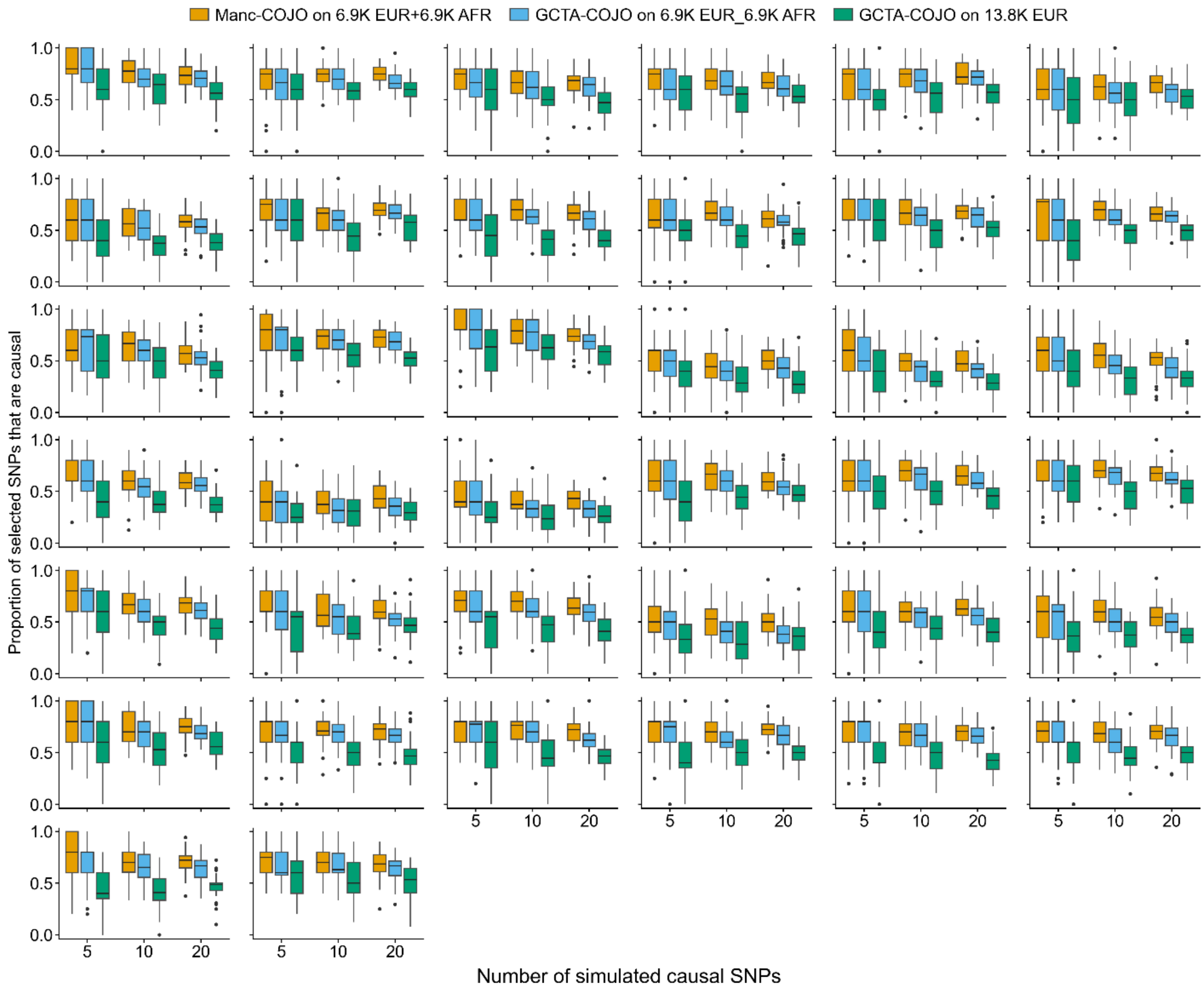

Supplementary Fig.2. Simulation results within individual blocks are consistent with the pooled results. This figure summarises the simulation results for all 38 blocks. The plot in the top-left represents Block 1, with Block 2 directly to its right, and so on. Within each plot, the x-axis denotes the number of simulated causal SNPs in that LD-independent block (5, 10, and 20 for the first, second, and third columns, respectively). The y-axis shows the proportion of COJO SNPs that are true causal SNPs. Overall, the results show that the trends observed within individual blocks align closely with those seen in the pooled analysis.

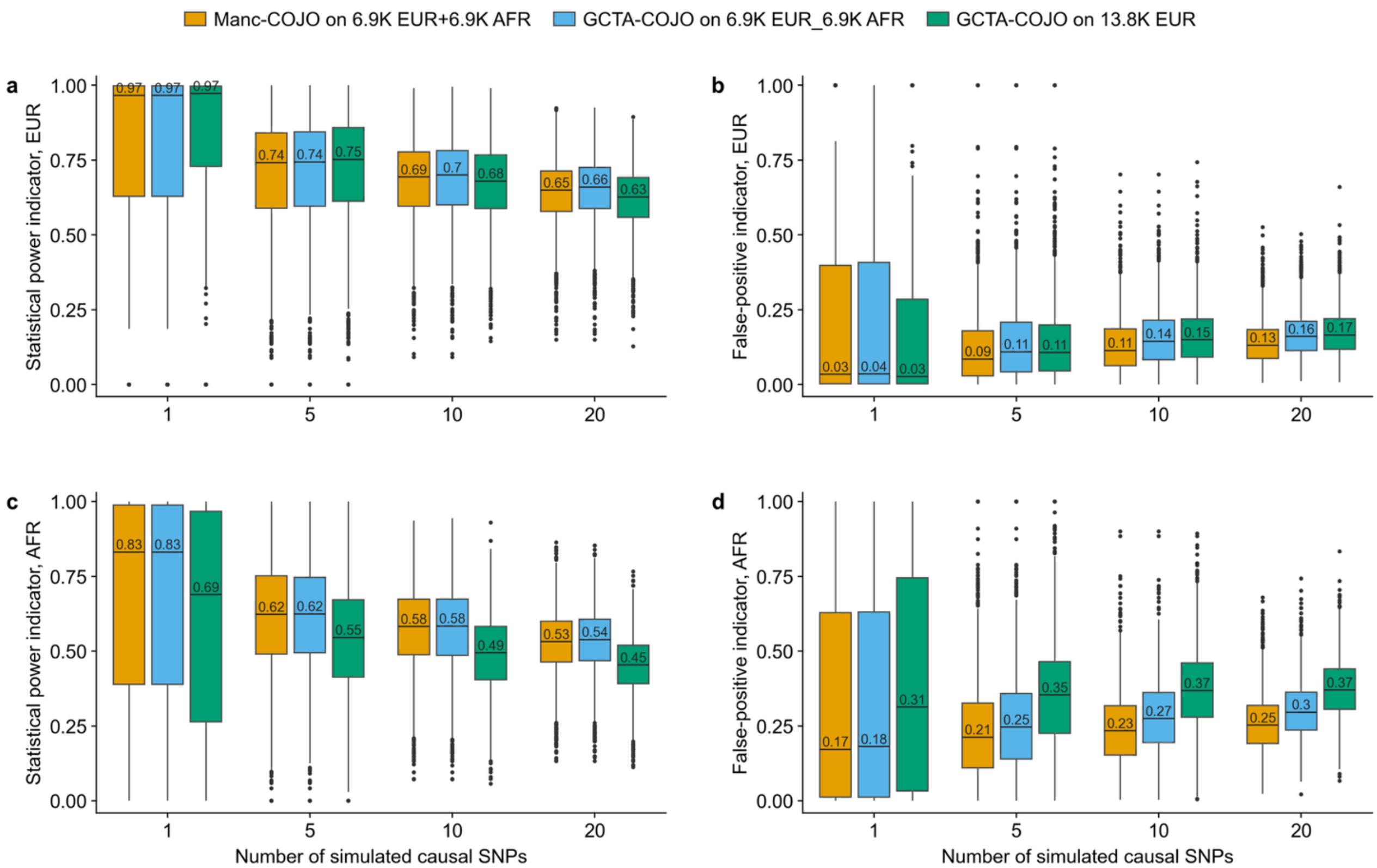

Supplementary Fig.3. Manc-COJO demonstrates superior performance in simulations when causal SNPs are not genotyped. This figure summarise simulation results of *N*=7,600 scenarios, and illustrates the performance of Manc-COJO in identifying causal SNPs, showing, on average, superior power and fewer false-positive discoveries across ancestry groups. We applied our algorithm to both a simulated EUR and a simulated AFR cohort, each with a sample size of *N*=6,901. Our comparisons include GCTA-COJO applied to either *N*=13,802 EUR individuals or a meta-analysis of *N*=6,901 EUR individuals with *N*=6,901 AFR individuals. In all scenarios, in-sample LD was utilised. Only Hapmap3 SNPs are included in the analysis. The X-axis represents the number of simulated causal SNPs. (a) We identified the COJO SNP with the highest absolute LD correlation (in European cohort) to each causal SNP and averaged these correlations across all causal SNPs. Higher value indicates better power in identifying causal variants. (b) For each COJO SNP, we identified the nearest true causal SNP, calculated their absolute (European) LD, and then averaged these LD values across all COJO SNPs. We then subtracted this mean from 1 to obtain an indicator of false-positive discoveries (i.e., when selected SNPs are not in LD with causal SNPs, the average absolute LD will be low, yielding a higher false-positive indicator). (c)(d) Power and false positive indicators as in (a) and (b), except that African LD is used in calculation. Numbers in each plots are the median value of each scenario.

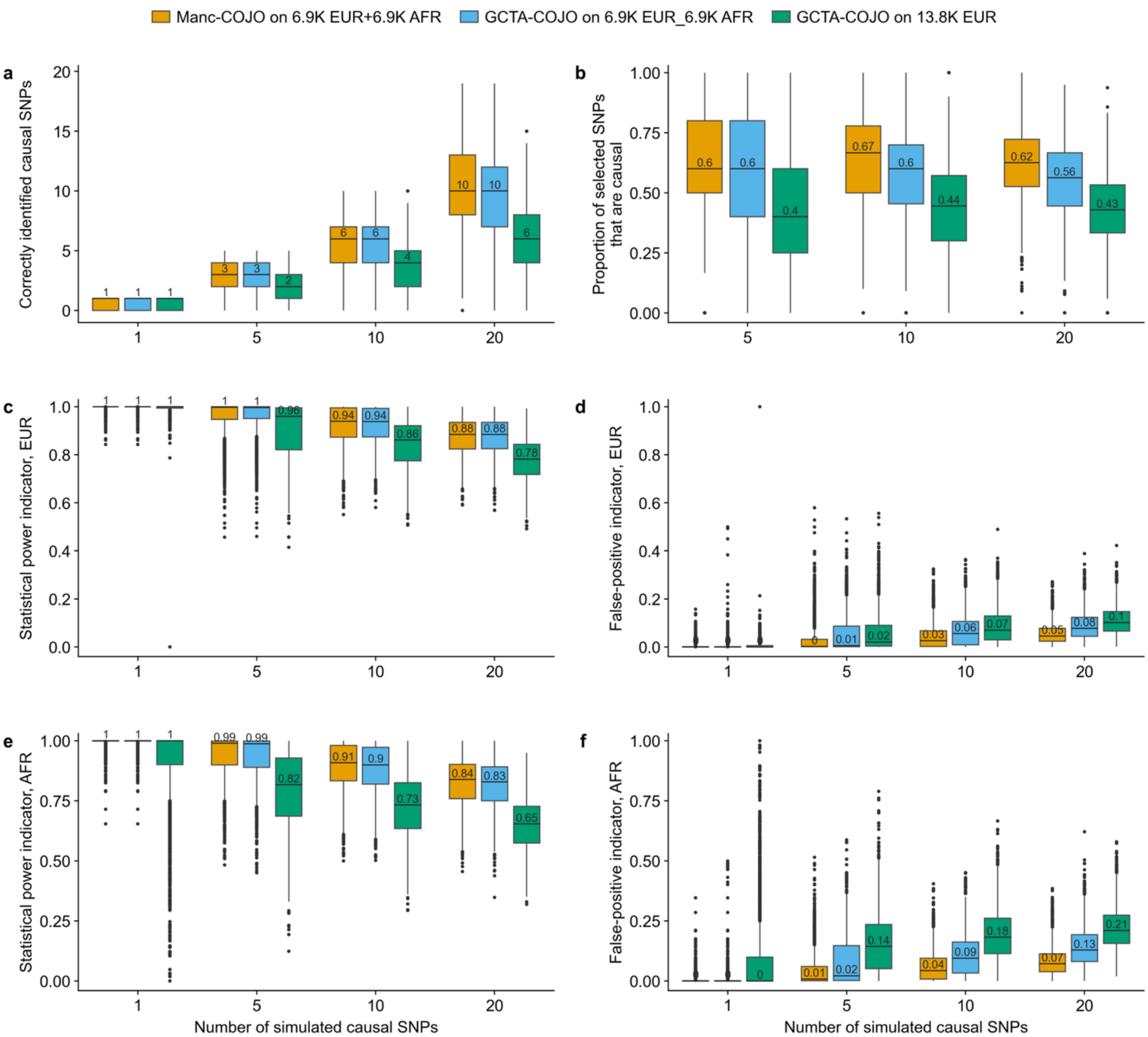

Supplementary Fig.4. **Manc-COJO demonstrates superior performance in simulations when effect sizes of causal SNPs are not identical across ancestry groups.** This figure summarises simulation results of *N*=7,600 scenarios, and illustrates the performance of Manc-COJO in identifying causal SNPs, showing, on average, superior power and fewer false-positive discoveries across ancestry groups. We applied our algorithm to both a EUR and a simulated AFR cohort, each with a sample size of *N*=6,901. Our comparisons include GCTA-COJO applied to either *N*=13,802 EUR individuals or a meta-analysis of *N*=6,901 EUR individuals with *N*=6,901 AFR individuals. In all cases, in-sample LD was utilised. The effect sizes of the causal SNPs varied across the simulated conditions, but the direction of association remained consistent. The X-axis represents the number of simulated causal SNPs. (a) We identified the COJO SNP with the highest absolute LD correlation (in European cohort) to each causal SNP and averaged these correlations across all causal SNPs. Higher value indicates better power in identifying causal variants. (b) For each COJO SNP, we identified the nearest true causal SNP, calculated their absolute (European) LD, and then averaged these LD values across all COJO SNPs. We then subtracted this mean from 1 to obtain an indicator of false-positive discoveries (i.e., when selected SNPs are not in LD with causal SNPs, the average LD will be low, yielding a higher false-positive indicator). (c)(d) Power and false positive indicators as in (a) and (b), except that African LD is used in calculation. Numbers in each plots are the median value of each scenario.

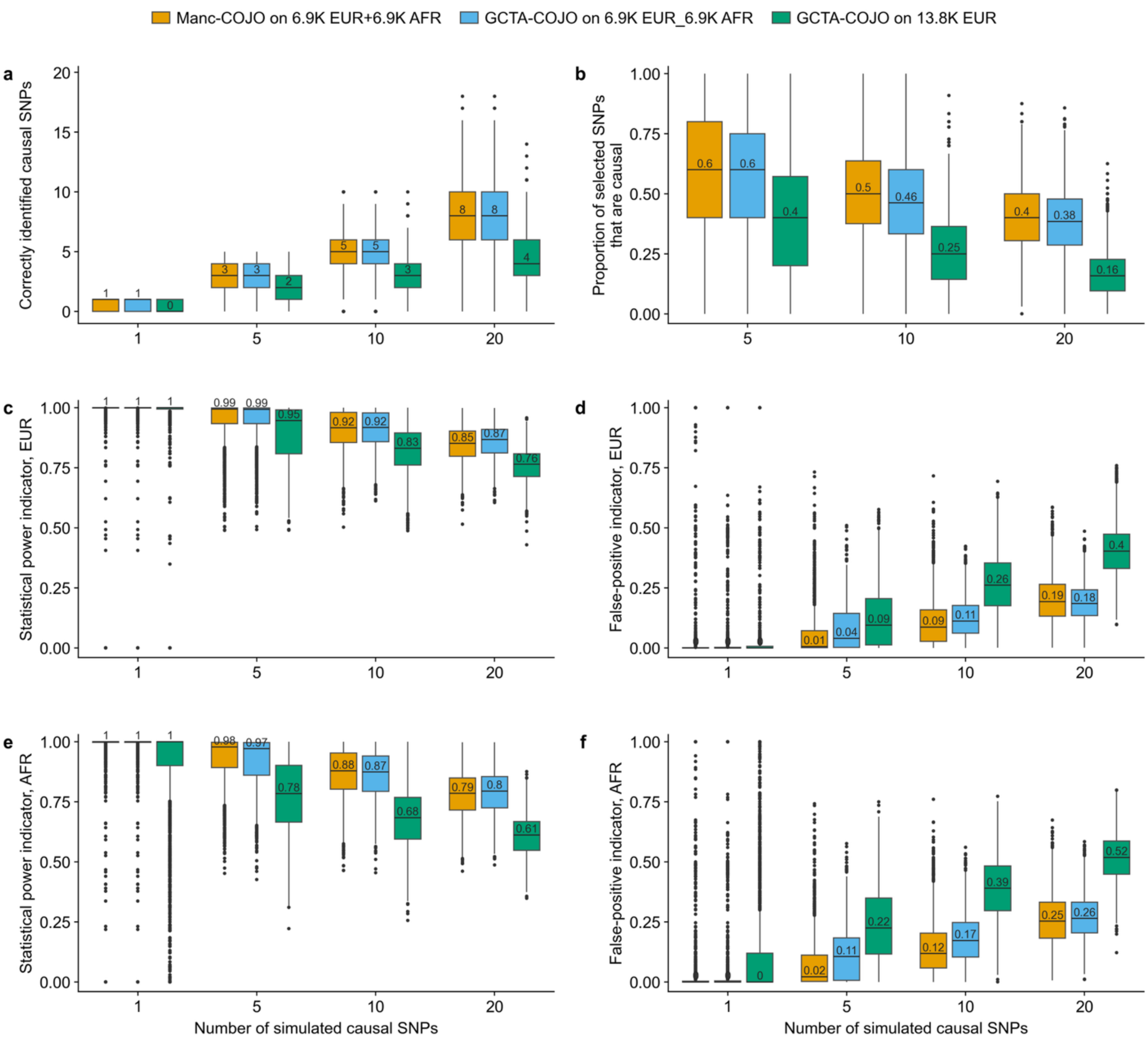

Supplementary Fig.5. Manc-COJO is robust to imperfect LD reference in simulations. This figure summarise simulation results of *N*=7,600 scenarios, and illustrates Manc-COJO is robust to imperfect LD reference. We applied our algorithm to both a simulated EUR and a simulated AFR cohort, each with a sample size of *N*=6,901. Our comparisons include GCTA-COJO applied to either *N*=13,802 EUR individuals or a meta-analysis of *N*=6,901 EUR individuals with *N*=6,901 AFR individuals. An LD reference from the 1000 Genomes Project was applied in both European (*N*=503) and African ancestry (*N*=503). The X-axis represents the number of simulated causal SNPs. (a) We identified the COJO SNP with the highest absolute LD correlation (in European cohort) to each causal SNP and averaged these correlations across all causal SNPs. Higher value indicates better power in identifying causal variants. (b) For each COJO SNP, we identified the nearest true causal SNP, calculated their absolute (European) LD, and then averaged these LD values across all COJO SNPs. We then subtracted this mean from 1 to obtain an indicator of false-positive discoveries (i.e., when selected SNPs are not in LD with causal SNPs, the average LD will be low, yielding a higher false-positive indicator). (c)(d) Power and false positive indicators as in (a) and (b), except that African LD is used in calculation. Numbers in each plots are the median value of each scenario.

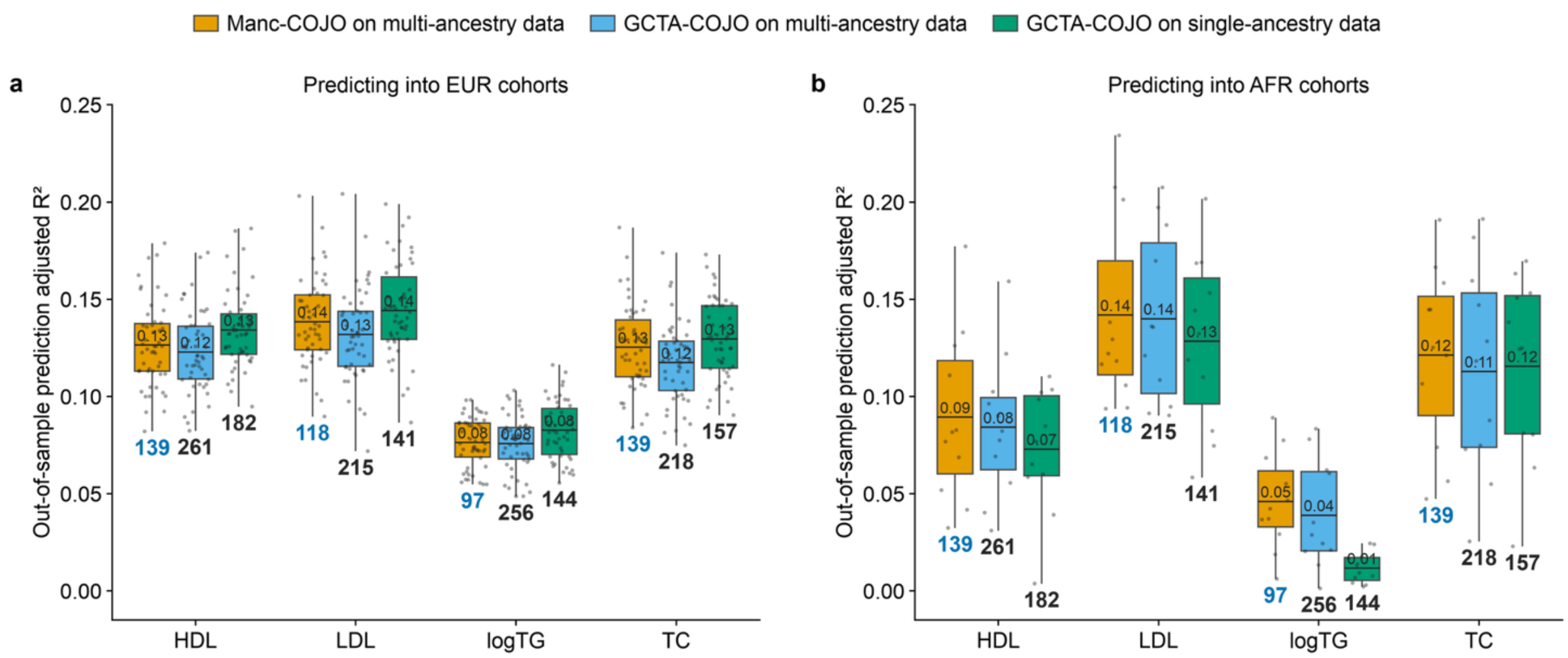

Supplementary Fig.6. Manc-COJO demonstrates superior cross-ancestry portability compared to the single-ancestry GCTA-COJO model in down-sample analysis applied in real data. We conducted a down-sampling analysis to examine whether closer ancestry balance (approximately 1:1) would enhance detection of independent associations. This figure summarises the out-of-sample prediction accuracies of Manc-COJO on multi-ancestry data, GCTA-COJO on multi-ancestry data, and GCTA-COJO on single-ancestral data for HDL, LDL, logTG, and TC when predicting into independent European and African cohorts. Numbers in the figure are sizes of the model (i.e., numbe of COJO-SNPs), and the model with the smallest number of SNPs is highlighted in blue for each trait. (i) Manc-COJO input: AFR-specific GWAS summary statistics from GLGC ($N \approx 90{,}000$), combined with new EUR-specific GWAS conducted in UK Biobank participants (N = 90,000) using pipelines consistent with the original GLGC studies. The LD reference panels consist of N=6,901 unrelated European individuals and N=6,901 unrelated African individuals from the UK Biobank. (ii) GCTA-COJO on multi-ancestry data input: Meta-analysis of the EUR-specific and AFR-specific GWAS summary statistics described above. The LD reference panel is a merged set of the unrelated European and African individuals from the UK Biobank (N=6,901 EUR individuals and N=6,901 AFR individuals). (iii) GCTA-COJO on single-ancestry data input: new EUR-specific GWAS conducted in UK Biobank participants (N=180,000) using pipelines consistent with the original GLGC studies. The LD reference panel was a set of N=13,802 unrelated European and African individuals from the UK Biobank. To ensure fair comparisons across methods, we harmonised the GWAS summary statistics to have identical SNP sets and approximately equal sample sizes, and we kept identical LD reference panel sizes across methods. Overall, Manc-COJO models showed slightly lower out-of-sample prediction accuracy into European cohorts compared to single-ancestry GCTA-COJO models. However, Manc-COJO models were much smaller and consistently demonstrated much better cross-ancestrycross-ancestry portability. Compared to the full-size analysis, the relative size difference between Manc-COJO and GCTA-COJO models decreased, suggesting that closer ancestry balance may enhance the detection of independent associations. Notably, GCTA-COJO models trained on multi-ancestry data continued to show sub-optimal out-of-sample performance across all scenarios.

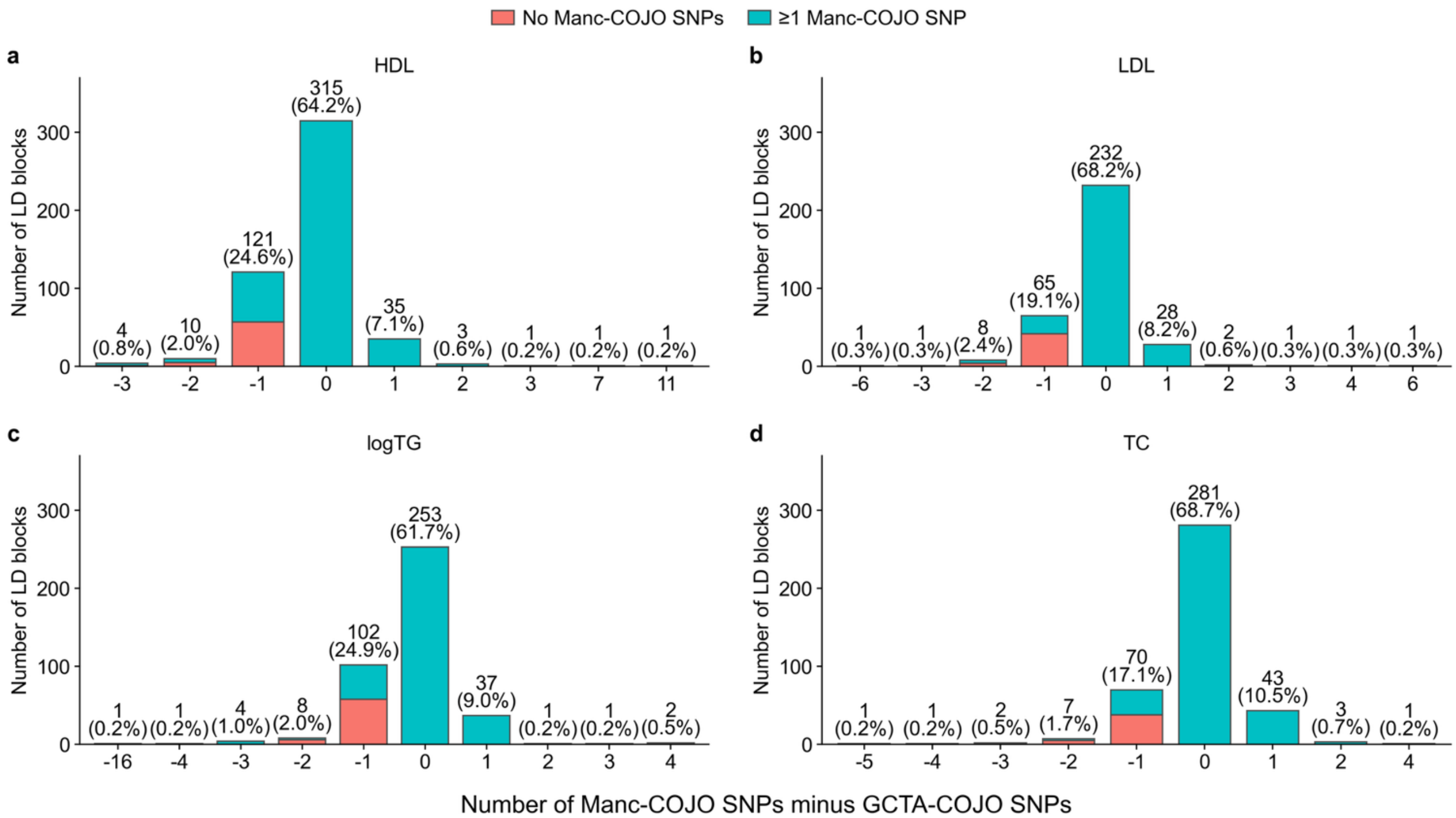

Supplementary Fig.7. Comparison of Manc-COJO and GCTA-COJO outputs. This figure compares the number of COJO SNPs selected by the Manc-COJO model (using GWAS input of 0.9M EUR + 0.09M AFR) with the single-ancestry GCTA-COJO model (using GWAS input of 0.9M EUR + 0.09M EUR) for (a) HDL, (b) LDL, (c) logTG, and (d) TC. COJO-selected SNPs in both models were partitioned into LD-independent blocks. The x-axis shows the difference in SNP counts between the models (number of Manc-COJO SNPs minus number of GCTA-COJO SNPs), where a positive value indicates that Manc-COJO identified more SNPs than GCTA-COJO. The y-axis represents the number of blocks. The number shown above each histogram bar is the count of blocks, with the percentage in parentheses indicating the proportion of all blocks corresponding to that x-value. The **red** sections of the histograms highlight blocks where the Manc-COJO model identified zero SNPs while the GCTA-COJO model identified more than one SNP.

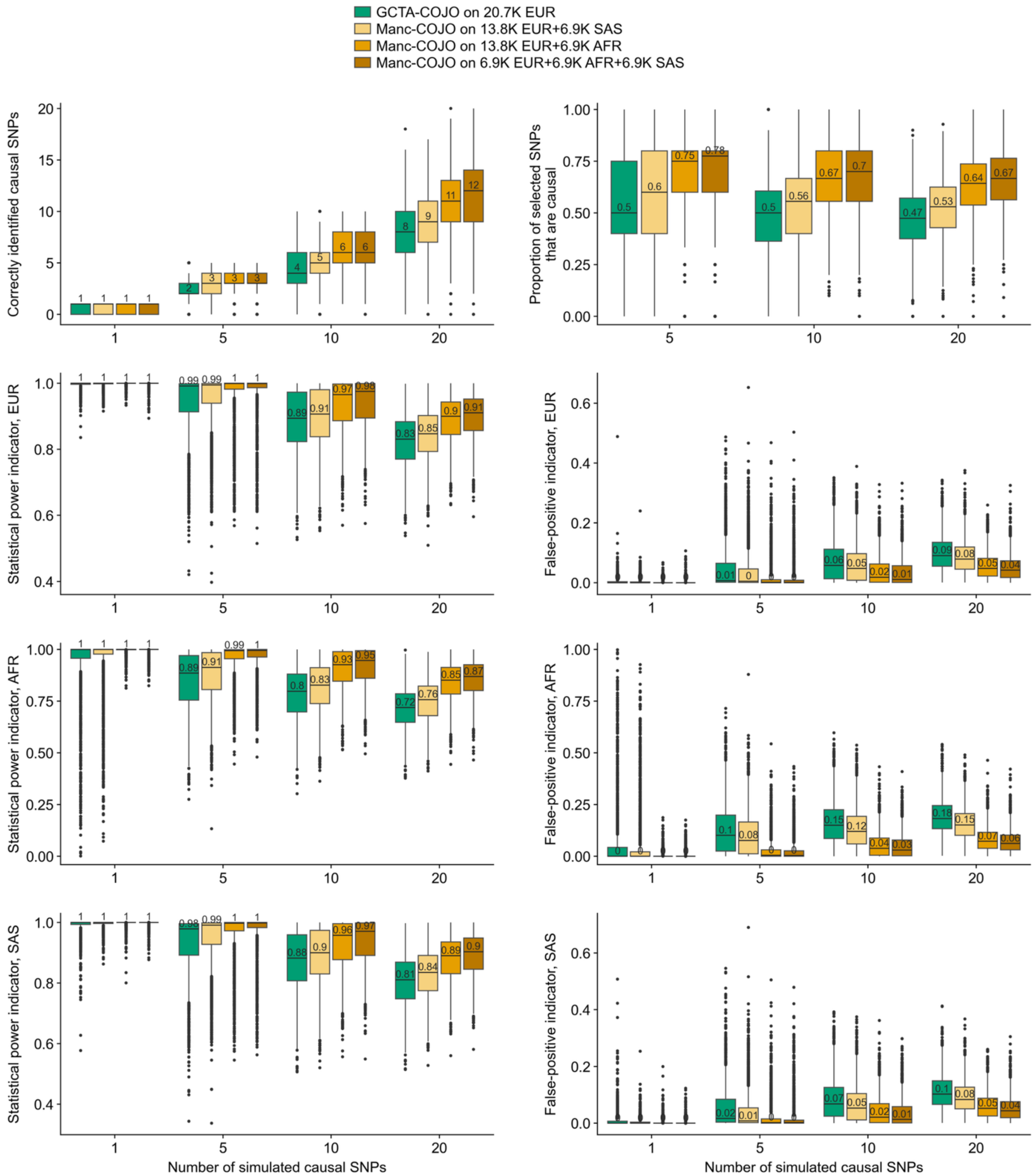

Supplementary Fig. 8. Benefit of incorporating additional ancestry groups into the analysis. This figure summarises simulation results from *N*=7,600 scenarios and demonstrates the benefit of incorporating well powered data from additional ancestry groups in increasing statistical power and reducing false positive discoveries when identifying independent associations. The simulated scenarios are described in Supplementary Note 8.1. The layout of this figure is identical to Fig. 2, except that the evaluation metrics, power (bottom left panel) and false positives (bottom right panel), were additionally calculated using linkage disequilibrium patterns in SAS. NB The total sample size in all analyses is higher here than in Fig. 2.

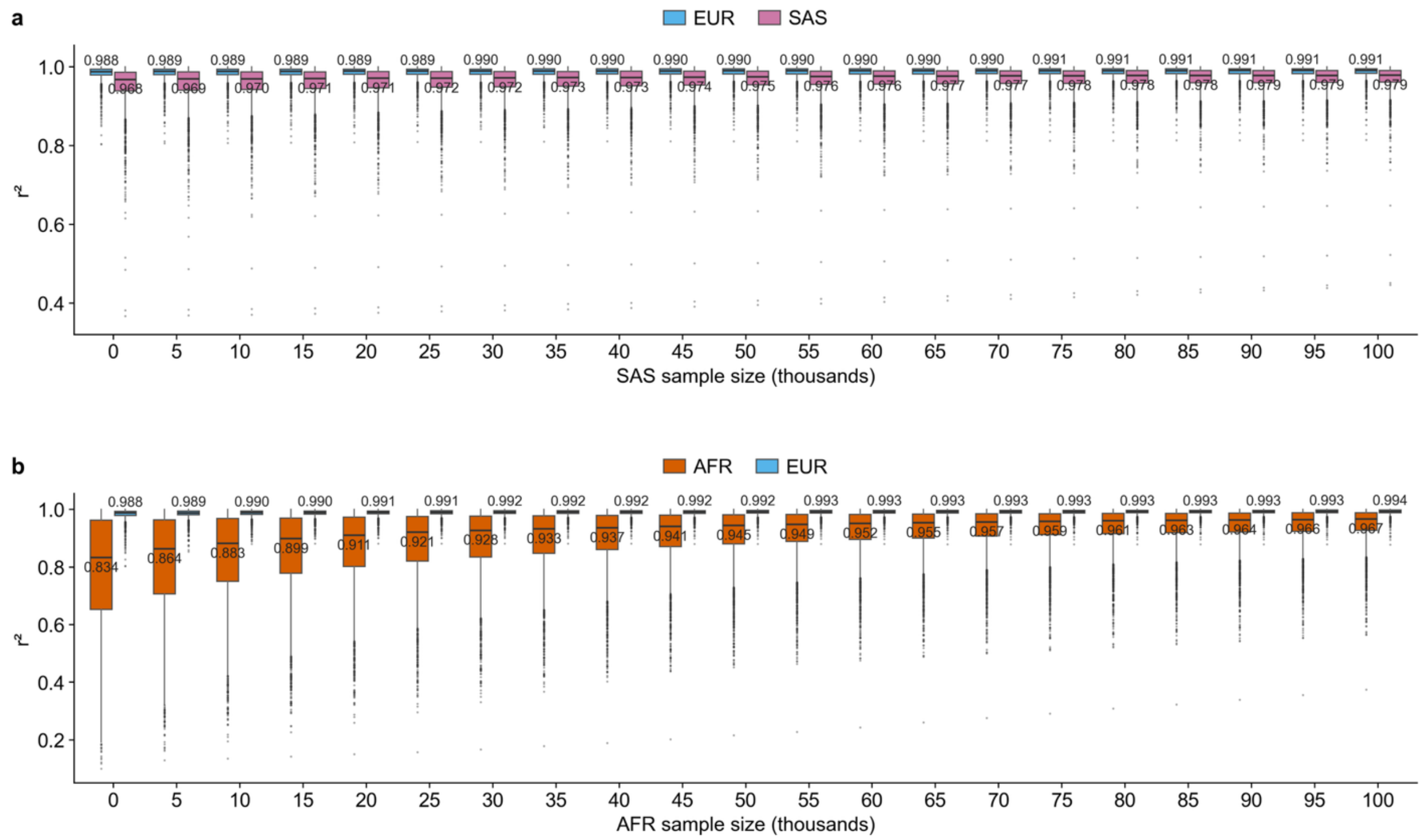

Supplementary Fig. 9. Impact of adding a second ancestry with limited sample size on the identification of independent associations. Simulations based on equation (19) in Supplementary Note 1.2.3 were conducted to evaluate whether incorporating ancestry groups with relatively small sample sizes improves the identification of causal variants. In a two ancestry setting, the EUR sample size was fixed at 0.5 million, while the SAS (x axis of panel a) or AFR (x axis of panel b) sample size increased from 0 to 0.1 million. Performance was assessed using the mean LD r^2 between the top associated SNP and the true causal variant, averaged across 10,000 simulations. The plots show the distribution of mean LD r^2 values (y axis) across all SNPs when each SNP is treated as the causal variant. Panels a and b show that adding an additional ancestry improves the ability of COJO to resolve independent signals, although the benefit is modest when the added sample size is small. This limitation is more pronounced for the EUR and SAS combination, consistent with their longer range LD structure compared to AFR.

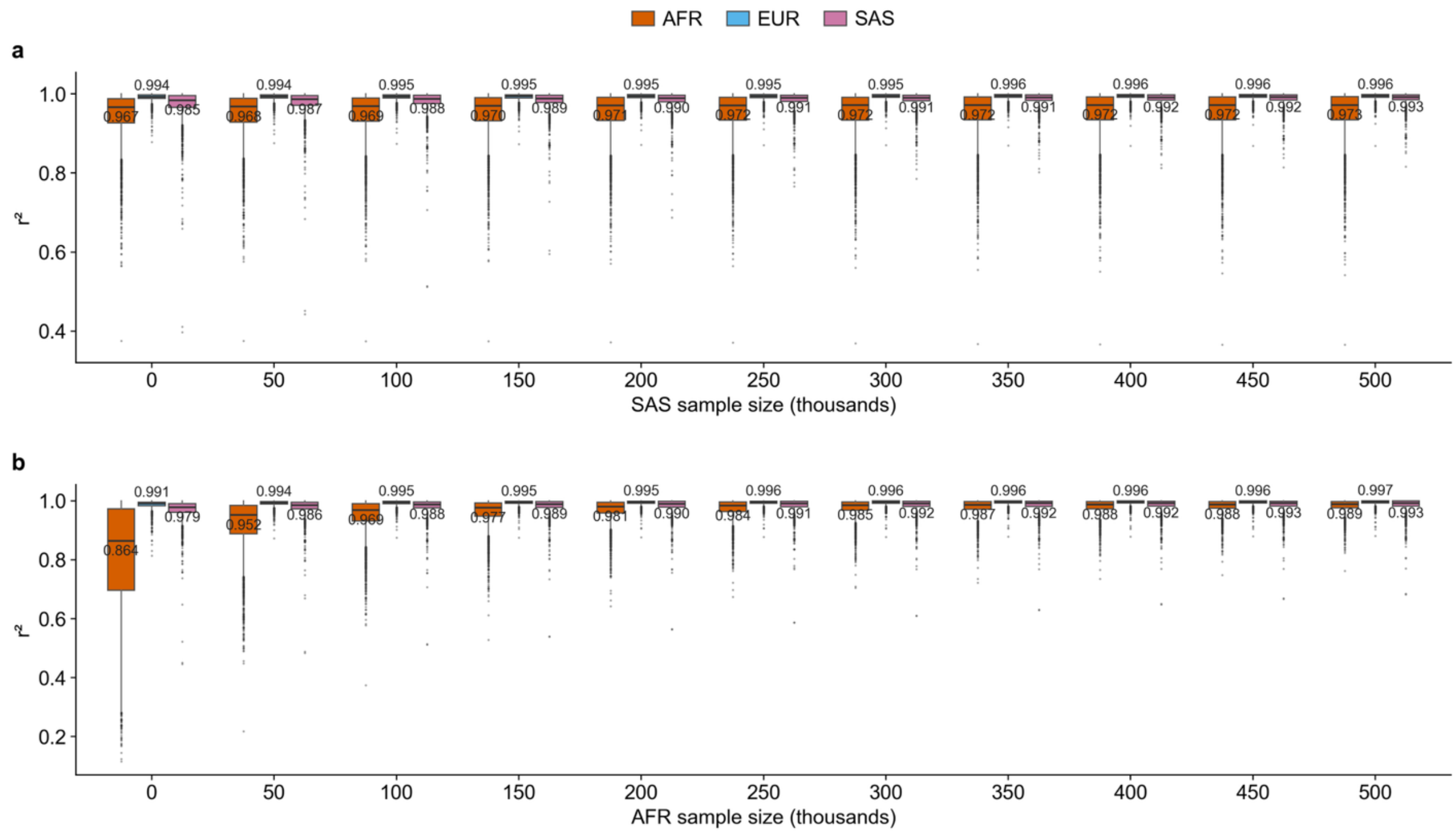

Supplementary Fig. 10. Impact of adding a third ancestry with limited sample size on the identification of independent associations. Simulations based on equation (19) in Supplementary Note 1.2.3 were conducted to evaluate whether incorporating a third ancestry improves the identification of causal variants. The EUR sample size was fixed at 0.5 million. In panel a, the AFR sample size was fixed at 0.1 million while the SAS sample size increased from 0 to 0.5 million (x-axis). In panel b, the SAS sample size was fixed at 0.1 million while the AFR sample size increased over the same range. Performance was assessed using the mean LD r^2 between the top associated SNP and the true causal variant, averaged across 10,000 simulations. The plots show the distribution of mean LD r^2 values (y axis) across all SNPs when each SNP is treated as the causal variant. Panels a and b show that increasing the sample size of the third ancestry improves the ability of COJO to resolve independent signals, with selected variants showing stronger correspondence to the true causal SNP. This improvement is more pronounced when increasing AFR sample size, consistent with its shorter LD structure compared to SAS.

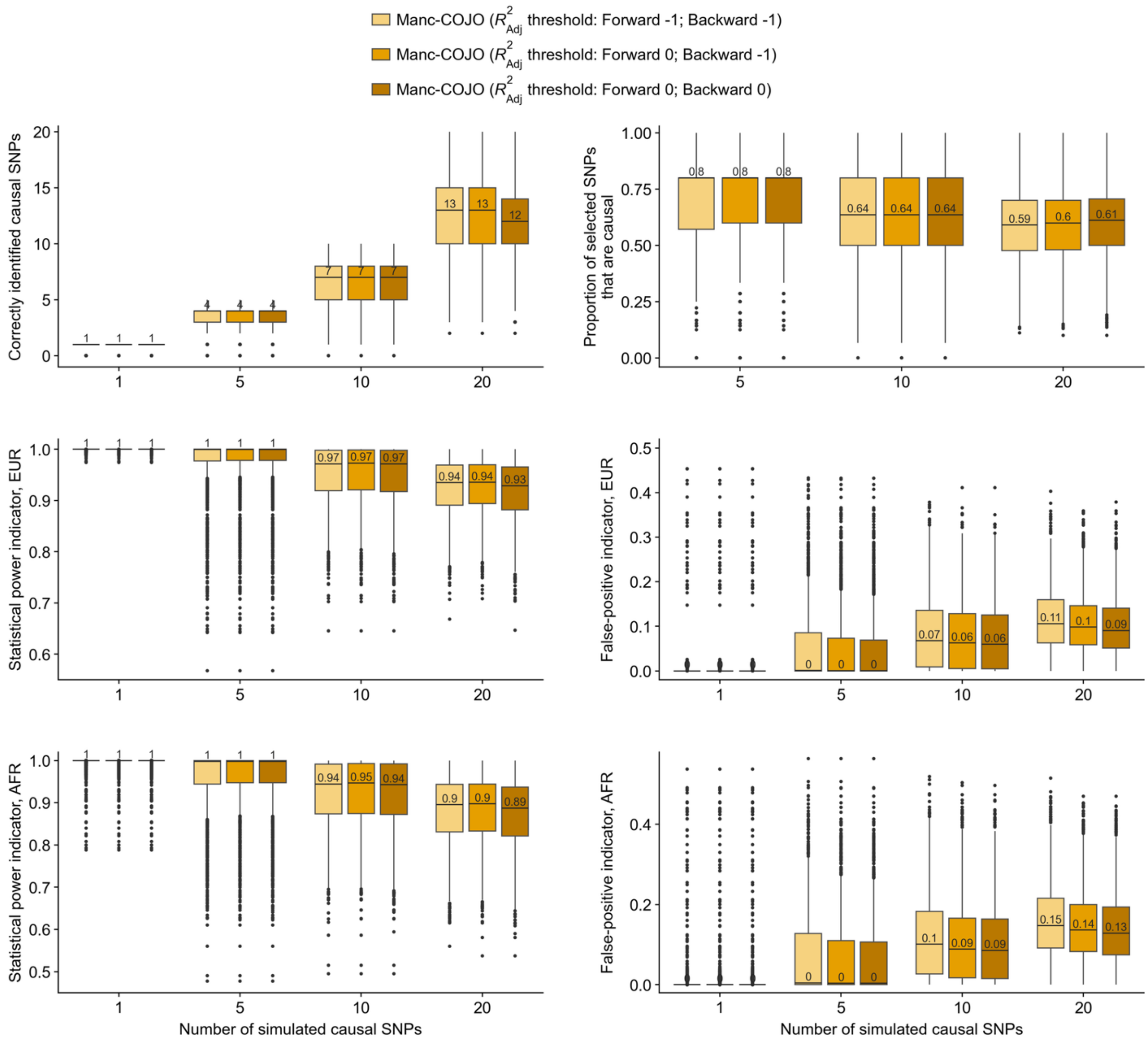

Supplementary Fig.11. Impact of different adjusted R^2 threshold on Manc-COJO output in idealised situations. The simulation settings and layout of this figure are identical to those in Fig.2. In this figure, we compare, under a well-powered scenario with a EUR:AFR ratio of 10:1, the impact of adding an additional optional variable selection algorithm based on the model's adjusted R^2. In general, applying a more stringent threshold reduces the false positive inclusion of non-causal SNPs in the model at the expense of reduced power to include causal SNPs.

References

1. Yang, J. *et al.* Conditional and joint multiple-SNP analysis of GWAS summary statistics identifies additional variants influencing complex traits. *Nature genetics* **44**, 369-375 (2012).
2. Bergstrom, C.T. & Dugatkin, L.A. *Evolution*, (WW Norton & Company, 2016).
3. Palmer, D.S. *et al.* Analysis of genetic dominance in the UK Biobank. *Science* **379**, 1341-1348 (2023).
4. Phillips, P.C. Epistasis—the essential role of gene interactions in the structure and evolution of genetic systems. *Nature Reviews Genetics* **9**, 855-867 (2008).
5. Polderman, T.J. *et al.* Meta-analysis of the heritability of human traits based on fifty years of twin studies. *Nature genetics* **47**, 702-709 (2015).
6. Hill, W.G., Goddard, M.E. & Visscher, P.M. Data and theory point to mainly additive genetic variance for complex traits. *PLoS genetics* **4**, e1000008 (2008).
7. Alkan, C., Sajjadian, S. & Eichler, E.E. Limitations of next-generation genome sequence assembly. *Nature methods* **8**, 61-65 (2011).
8. Li, A. *et al.* mBAT-combo: a more powerful test to detect gene-trait associations from GWAS data. *The American Journal of Human Genetics* **110**, 30-43 (2023).
9. Wang, Y. *et al.* Theoretical and empirical quantification of the accuracy of polygenic scores in ancestry divergent populations. *Nature communications* **11**, 3865 (2020).
10. Schaid, D.J., Chen, W. & Larson, N.B. From genome-wide associations to candidate causal variants by statistical fine-mapping. *Nature Reviews Genetics* **19**, 491-504 (2018).
11. van de Bunt, M. *et al.* Evaluating the performance of fine-mapping strategies at common variant GWAS loci. *PLoS genetics* **11**, e1005535 (2015).
12. Graham, S.E. *et al.* The power of genetic diversity in genome-wide association studies of lipids. *Nature* **600**, 675-679 (2021).
13. Shi, H. *et al.* Localizing components of shared transethnic genetic architecture of complex traits from GWAS summary data. *The American Journal of Human Genetics* **106**, 805-817 (2020).
14. Yengo, L. *et al.* A saturated map of common genetic variants associated with human height. *Nature* **610**, 704-712 (2022).
15. Privé, F., Arbel, J., Aschard, H. & Vilhjálmsson, B.J. Identifying and correcting for misspecifications in GWAS summary statistics and polygenic scores. *Human Genetics and Genomics Advances* **3**(2022).
16. Kanai, M. *et al.* Meta-analysis fine-mapping is often miscalibrated at single-variant resolution. *Cell genomics* **2**(2022).
17. Chen, W. *et al.* Improved analyses of GWAS summary statistics by reducing data heterogeneity and errors. *Nature Communications* **12**, 7117 (2021).
18. Momin, M.M., Lee, S., Wray, N.R. & Lee, S.H. Significance tests for R2 of out-of-sample prediction using polygenic scores. *The American journal of human genetics* **110**, 349-358 (2023).
19. Dias, J.-A. *et al.* Evaluating Multi-Ancestry Genome-Wide Association Methods: Statistical Power, Population Structure, and Practical Implications. *medRxiv* (2025).

20. Lee, H., Lee, M.H., Hou, K., Pasaniuc, B. & Han, B. Admixed and single-continental genome segments of the same ancestry have distinct linkage disequilibrium patterns. *Genome biology* **26**, 201 (2025).
21. Rossen, J. *et al.* MultiSuSiE improves multi-ancestry fine-mapping in All of Us whole-genome sequencing data. *Nature Genetics*, 1-10 (2026).
22. Gao, B. & Zhou, X. MESuSiE enables scalable and powerful multi-ancestry fine-mapping of causal variants in genome-wide association studies. *Nature genetics* **56**, 170-179 (2024).
