# Supplementary material for: Multiancestry conditional and joint analysis applied to GWAS summary statistics
Source: Nat Genet. 2026 Oct 20;58(11):3019–27. doi: 10.1038/s41588-026-02799-1 (PMC13754604; doi:10.1038/s41588-026-02799-1)
Supplement: Supplementary file 2 [file 41588_2026_2799_MOESM2_ESM.pdf]

nature portfolio

Corresponding author(s): Xiaotong Wang

Last updated by author(s): Aug 3, 2026

Reporting Summary

Nature Portfolio wishes to improve the reproducibility of the work that we publish. This form provides structure for consistency and transparency in reporting. For further information on Nature Portfolio policies, see our Editorial Policies and the Editorial Policy Checklist.

Statistics

For all statistical analyses, confirm that the following items are present in the figure legend, table legend, main text, or Methods section.

n/a	Confirmed	
☐	☒	The exact sample size (*n*) for each experimental group/condition, given as a discrete number and unit of measurement
☒	☐	A statement on whether measurements were taken from distinct samples or whether the same sample was measured repeatedly
☐	☒	The statistical test(s) used AND whether they are one- or two-sided<br>*Only common tests should be described solely by name; describe more complex techniques in the Methods section.*
☐	☒	A description of all covariates tested
☐	☒	A description of any assumptions or corrections, such as tests of normality and adjustment for multiple comparisons
☐	☒	A full description of the statistical parameters including central tendency (e.g. means) or other basic estimates (e.g. regression coefficient) AND variation (e.g. standard deviation) or associated estimates of uncertainty (e.g. confidence intervals)
☐	☒	For null hypothesis testing, the test statistic (e.g. *F*, *t*, *r*) with confidence intervals, effect sizes, degrees of freedom and *P* value noted<br>*Give P values as exact values whenever suitable.*
☒	☐	For Bayesian analysis, information on the choice of priors and Markov chain Monte Carlo settings
☒	☐	For hierarchical and complex designs, identification of the appropriate level for tests and full reporting of outcomes
☐	☒	Estimates of effect sizes (e.g. Cohen's *d*, Pearson's *r*), indicating how they were calculated

Our web collection on statistics for biologists contains articles on many of the points above.

Software and code

Policy information about availability of computer code

Data collection	No data collection software was used. The data utilised are described in detail in the manuscript and comprise datasets from multiple large-scale biobanks.
Data analysis	Summary statistics were processed using R v4.3.2 with the data.table package v1.17.8. Causal variants and effect sizes were simulated using custom R scripts, with genotype data imported using the genio package v1.1.2. Simulated GWAS were performed using PLINK 2.0 v2.00a2.3. Meta-analysis was conducted using custom scripts that formatted the resulting summary statistics for input into GCTA-COJO. Conditional and joint analyses were performed using Manc-COJO v1.1.0 and GCTA v1.93.2 beta, and polygenic risk scores were calculated using PLINK v1.90b6.21. The regional association plot in Fig. 5 was generated using the R package locuszoomr v0.3.8 with gene annotations from EnsDb.Hsapiens.v75 v2.99.0. All other plots were generated using Python v3.11.3 with the packages matplotlib v3.11.1, NumPy v2.5.1, and pandas v3.0.5. All custom code developed for this study is available on GitHub (https://github.com/light156/multi-ancestry-COJO) and archived on Zenodo (10.5281/zenodo.21704762).

For manuscripts utilizing custom algorithms or software that are central to the research but not yet described in published literature, software must be made available to editors and reviewers. We strongly encourage code deposition in a community repository (e.g. GitHub). See the Nature Portfolio guidelines for submitting code & software for further information.

Data

Policy information about availability of data

All manuscripts must include a data availability statement. This statement should provide the following information, where applicable:

- Accession codes, unique identifiers, or web links for publicly available datasets
- A description of any restrictions on data availability
- For clinical datasets or third party data, please ensure that the statement adheres to our policy

UK Biobank and All of Us data are available through formal application to the UK Biobank (https://www.ukbiobank.ac.uk) and the All of Us Research Hub (https://www.researchallofus.org), respectively. GWAS summary statistics for the four lipid traits analysed in this study are publicly available from the GLGC (https://csg.sph.umich.edu/willer/public/glgc-lipids2021/). The 1000G Project genotype data used for linkage disequilibrium reference comparisons are available at https://figshare.com/articles/dataset/HapMap3-1KG_1000_Genomes_processed_genotypes_in_PLINK_bed_bim_fam_format/20802700.

Research involving human participants, their data, or biological material

Policy information about studies with human participants or human data. See also policy information about sex, gender (identity/presentation), and sexual orientation and race, ethnicity and racism.

Reporting on sex and gender	Biological sex was defined using reported sex in the UK Biobank (Data-Field 31) and sex assigned at birth in the All of Us dataset. Analyses were performed in a sex-stratified manner, with residuals computed separately for males and females and subsequently pooled. Further details are provided in the main text.
Reporting on race, ethnicity, or other socially relevant groupings	In this study, we used discrete continental genetic ancestry classifications consistent with those defined in the 1000 Genomes Project. For the All of Us data, ancestry classifications were based on the ancestry_pred_other column from the srWGS auxiliary file. In the UK Biobank, genetic ancestry was defined using principal component–based projections as described by Wang et al., with further details provided in the main text.
Population characteristics	Population characteristics for UK Biobank and All of Us participants are available on the respective study websites (UK Biobank: https://www.ukbiobank.ac.uk; All of Us Research Program: https://allofus.nih.gov). In brief, the All of Us Research Program CDRv8 Controlled Tier included data from 633,547 participants who enrolled and consented before October 1, 2023; 62.50% reported female sex assigned at birth, and 20.66% were aged 60–69 years. UK Biobank recruited 503,317 participants aged 40–69 years between 2006 and 2010. At recruitment, 24% of participants were aged 40–49 years, 34% were aged 50–59 years, and 42% were aged 60–69 years; 54% were female and 46% were male according to National Health Service records.
Recruitment	We did not recruit participants for this study. All analyses were based on publicly available datasets and GWAS summary statistics. Recruitment details for UK Biobank and All of Us participants are available on the respective study websites.
Ethics oversight	All analyses in this study were based on publicly available datasets and GWAS summary statistics. Ethical approvals for each biobank are available on their respective websites. In brief, the UK Biobank protocol and Research Tissue Bank were approved by the North West–Haydock Research Ethics Committee, formerly known as the National Research Ethics Service Committee North West–Haydock (original REC reference 11/NW/0382; subsequent renewal references 16/NW/0274 and 21/NW/0157). The All of Us Research Program protocol, informed-consent documents, and participant-facing materials were approved by the All of Us Institutional Review Board.

Note that full information on the approval of the study protocol must also be provided in the manuscript.

Field-specific reporting

Please select the one below that is the best fit for your research. If you are not sure, read the appropriate sections before making your selection.

☒ Life sciences ☐ Behavioural & social sciences ☐ Ecological, evolutionary & environmental sciences

For a reference copy of the document with all sections, see nature.com/documents/nr-reporting-summary-flat.pdf

Life sciences study design

All studies must disclose on these points even when the disclosure is negative.

Sample size	The median per-SNP sample sizes and the number of SNPs included for each trait were as follows: HDL (NAFR = 90,804; NEUR = 881,859; M = 6,514,392 SNPs), LDL (NAFR = 87,759; NEUR = 837,509; M = 6,511,658 SNPs), TC (NAFR = 92,554; NEUR = 924,769; M = 6,514,274 SNPs), and logTG (NAFR = 89,467; NEUR = 858,015; M = 6,514,129 SNPs). These datasets were considered sufficient because they were derived from some of the largest ancestry-specific GWAS conducted to date, providing adequate statistical power and supporting robust, comparable analyses across traits and ancestry groups. Sample sizes varied according to the original GWAS summary statistics and the quality control procedures applied, which are described in detail in the Supplementary Information.

Data exclusions	In both the UK Biobank and All of Us datasets, analyses were restricted to common, bi-allelic SNPs (minor allele frequency [MAF] ≥ 0.01) present in both European and African ancestries. For analyses involving the All of Us dataset, SNPs not shared between the UK Biobank and All of Us cohorts at the start of the analysis were additionally excluded. Further details are provided in the main text.
Replication	In the simulations, we performed 50 replicates for each combination of block structure and number of causal SNPs, resulting in 7,600 simulated scenarios. For the real data analyses, we replicated our analyses both within biobanks—by repeating them across multiple randomly assigned cohorts—and across biobanks, by conducting the analyses in the All of Us dataset. Across all settings, the results showed consistent trends.
Randomization	Randomisation for sample collection is not applicable, as this study uses GWAS summary statistics from publicly available datasets. Randomisation was applied to the simulation samples and to the samples used for out-of-sample prediction. Further details are provided in the main text.
Blinding	Blinding was not applicable in this study, as it relies on GWAS summary statistics from publicly available datasets. No component of the study design required blinding. Further details are provided in the main text.

Reporting for specific materials, systems and methods

We require information from authors about some types of materials, experimental systems and methods used in many studies. Here, indicate whether each material, system or method listed is relevant to your study. If you are not sure if a list item applies to your research, read the appropriate section before selecting a response.

Materials & experimental systems

n/a	Involved in the study
☒	☐ Antibodies
☒	☐ Eukaryotic cell lines
☒	☐ Palaeontology and archaeology
☒	☐ Animals and other organisms
☒	☐ Clinical data
☒	☐ Dual use research of concern
☒	☐ Plants

Methods

n/a	Involved in the study
☒	☐ ChIP-seq
☒	☐ Flow cytometry
☒	☐ MRI-based neuroimaging

Plants

Seed stocks	*Report on the source of all seed stocks or other plant material used. If applicable, state the seed stock centre and catalogue number. If plant specimens were collected from the field, describe the collection location, date and sampling procedures.*
Novel plant genotypes	*Describe the methods by which all novel plant genotypes were produced. This includes those generated by transgenic approaches, gene editing, chemical/radiation-based mutagenesis and hybridization. For transgenic lines, describe the transformation method, the number of independent lines analyzed and the generation upon which experiments were performed. For gene-edited lines, describe the editor used, the endogenous sequence targeted for editing, the targeting guide RNA sequence (if applicable) and how the editor was applied.*
Authentication	*Describe any authentication procedures for each seed stock used or novel genotype generated. Describe any experiments used to assess the effect of a mutation and, where applicable, how potential secondary effects (e.g. second site T-DNA insertions, mosiacism, off-target gene editing) were examined.*
