# Supplementary material for: Multiancestry conditional and joint analysis applied to GWAS summary statistics
Source: Nat Genet. 2026 Oct 20;58(11):3019–27. doi: 10.1038/s41588-026-02799-1 (PMC13754604; doi:10.1038/s41588-026-02799-1)
Supplement: Supplementary file 3 [file 41588_2026_2799_MOESM3_ESM.pdf]

nature portfolio

Peer Review File

Multi-ancestry conditional and joint analysis applied to GWAS summary statistics

Corresponding Author: Dr Xiaotong Wang

Version 0:

Decision Letter:

26th Feb 2026

Dear Dr Wang,

Your Technical Report, "Multi-ancestry conditional and joint analysis (Manc-COJO) applied to GWAS summary statistics" has now been seen by 3 referees. You will see from their comments below that while they find your work of interest, some important points are raised. We are interested in the possibility of publishing your study in Nature Genetics, but would like to consider your response to these concerns in the form of a revised manuscript before we make a final decision on publication.

To guide the scope of the revisions, the editors discuss the referee reports in detail within the team, including with the chief editor, with a view to identifying key priorities that should be addressed in revision and sometimes overruling referee requests that are deemed beyond the scope of the current study. We hope that you will find the prioritized set of referee points to be useful when revising your study. Please do not hesitate to get in touch if you would like to discuss these issues further.

We therefore invite you to revise your manuscript taking into account all reviewer and editor comments. Please highlight all changes in the manuscript text file. At this stage we will need you to upload a copy of the manuscript in MS Word .docx or similar editable format.

We are committed to providing a fair and constructive peer-review process. Do not hesitate to contact us if there are specific requests from the reviewers that you believe are technically impossible or unlikely to yield a meaningful outcome.

When revising your manuscript:

*1) Include a “Response to referees” document detailing, point-by-point, how you addressed each referee comment. If no action was taken to address a point, you must provide a compelling argument. This response will be sent back to the referees along with the revised manuscript.

*2) If you have not done so already please begin to revise your manuscript so that it conforms to our Technical Report format instructions, available
here.
Refer also to any guidelines provided in this letter.

*3) Include a revised version of any required Reporting Summary: https://www.nature.com/documents/nr-reporting-summary.pdf
It will be available to referees (and, potentially, statisticians) to aid in their evaluation if the manuscript goes back for peer review.
A revised checklist is essential for re-review of the paper.

Please be aware of our guidelines on digital image standards.

EXTENDED DATA FIGURES

When re-submitting your manuscript, please ensure that any supplementary figures and tables that are crucial to the

manuscript's conclusions are converted into Extended Data figures and tables to increase visibility of these data. Extended Data figures and tables are online-only (present in the online PDF and full-text HTML versions of the paper), peer-reviewed display items that provide essential background to the article but are not included in the main article due to space constraints. A maximum of ten Extended Data display items (figures and tables) is permitted.

Please use the link below to submit your revised manuscript and related files:

Link Redacted

Note: This URL links to your confidential home page and associated information about manuscripts you may have submitted, or that you are reviewing for us. If you wish to forward this email to co-authors, please delete the link to your homepage.

We hope to receive your revised manuscript within four to eight weeks. If you cannot send it within this time, please let us know.

Please do not hesitate to contact me if you have any questions or would like to discuss these revisions further.

Nature Genetics is committed to improving transparency in authorship. As part of our efforts in this direction, we are now requesting that all authors identified as 'corresponding author' on published papers create and link their Open Researcher and Contributor Identifier (ORCID) with their account on the Manuscript Tracking System (MTS), prior to acceptance. ORCID helps the scientific community achieve unambiguous attribution of all scholarly contributions. You can create and link your ORCID from the home page of the MTS by clicking on 'Modify my Springer Nature account'. For more information please visit please visit www.springernature.com/orcid.

We look forward to seeing the revised manuscript and thank you for the opportunity to review your work.

Sincerely,

Margot Brandt, PhD
Senior Editor
Nature Genetics
https://orcid.org/0000-0002-9434-794X

Reviewers' Comments:

Reviewer #1 (Remarks to the Author):

Wang et al. introduce Manc-COJO, a method for multi-ancestry COJO analysis. They also introduce Manc-COJO:MDISA, a follow-up approach to identify ancestry-specific associations. Their methodology is implemented in an efficient tool that is computationally fast and enables genome-wide analyses. The manuscript is well written, statistical features are discussed in detail, limitations are discussed, and extensive simulation studies and real data analyses illustrate and investigate the new methodology. The results show that Manc-COJO outperforms existing approaches in suitable scenarios and has potentially important applications in the investigation of GWAS results from meta-analyses across multiple ancestries.

• In an admixed study population where distinct ancestral groups cannot be clearly separated, and individual-level genotype data are available, is it appropriate to perform the analysis as a single-ancestry study using an LD matrix estimated from the full sample? Or what would be the recommendation here?
• How would a user decide to run Manc-COJO:MDISA as an additional analysis? Or is it best practice to always run Manc-COJO and Manc-COJO:MDISA? Steps 1-8 in the Methods section suggest that it is an integrated part of the analysis.
• Some meta-analyses incorporate ancestries with a relatively small sample size. What is a lower sample size cutoff to get reasonable results?
• Does the software output heterogeneity measures for effect sizes across ancestries?
• I appreciate the discussion about the fine mapping abilities of the Manc-COJO approach. I wonder if the authors could compare their approach with recent multi-ancestry fine-mapping approaches such as MultiSuSiE or MESuSie and discuss in more detail what the advantage of using COJO is compared to performing fine-mapping.
• 'First, we made several assumptions: true causal SNPs act additively on the phenotype, causal SNPs are independent, and the number of true causal variants, as well as their effect sizes on standardised phenotypes, are identical across ancestries.' Can the authors clarify the assumption that the causal SNPs are independent?
• I think the methodology can incorporate varying sample sizes across SNPs; can the authors emphasize and discuss this in the text? (PMID: 36643910)

Reviewer #1 (Remarks on code availability):

The GitHub repository contains usage instructions and example data. While I have not run the software myself, the materials provided suggest that reproducing the results described in the manuscript should be feasible.

Reviewer #2 (Remarks to the Author):

This paper introduces a new method Manc-COJO, which is a multi-ancestry extension of the classical COJO method, aimed to identify conditionally independent GWAS signals using ancestry-specific LD and summary statistics. The author gave theoretical analysis and developed an efficient software with C++ to implement the proposed method. By conducting extensive simulations and real data analysis to a couple of traits in the paper, the author demonstrates the advantage of the proposed method over the existing COJO framework and other ad hoc methods. Overall, the paper is technically strong, well-organized and clearly written.

Below are some of my concerns:

1.The Manc-COJO utilized the stepwise selection method (forward/backward), how will the author assess the selection stability? How sensitive are the results to the chosen p-value threshold? Some discussions to address the above questions might be helpful.

2.The author demonstrates the superiority of the proposed method with two different ancestries, how would the author expect the performance if more ancestries are included? As the sample size between the ancestries might be imbalanced, for example, if Eur>>Afr>>South Asia, will the extremely small sample size of the third population affect the performance of the other two in the proposed method?

Reviewer #2 (Remarks on code availability):

The author has clearly tutorial and guidance on how to install and use the software on Github.

Reviewer #3 (Remarks to the Author):

The popular GCTA-COJO method for conditional and joint analysis within a single ancestry is extended to multiple ancestries, Manj-COJO. Importantly, the authors also introduce Manc-COJO:MDISA, which identifies ancestry-specific variants that would be missed by Manc-COJO. A faster COJO implementation is also provided in their new software.

Various simulation settings involving two ancestries are explored, including sensitivity analyses.

Some minor comments
1. The method overview should clearly state that Manj-COJO only considers variants that are present in all ancestries.
2. When there are more than two ancestries, is it possible to identify variants that are present in and associated with a subset of the ancestries (e.g. variant present in European and East Asian and associated with the trait in both ancestries, but the variant is rare in African so not present in the African GWAS)?

Reviewer #3 (Remarks on code availability):

Clear installation instructions are provided, together with a tutorial using example data.

Version 1:

Decision Letter:

Our ref: NG-TR71531R

9th Jun 2026

Dear Dr. Wang,

Thank you for submitting your revised manuscript "Multi-ancestry conditional and joint analysis (Manc-COJO) applied to GWAS summary statistics" (NG-TR71531R). It has now been seen by the original referees and their comments are below. The reviewers find that the paper has improved in revision, and therefore we'll be happy in principle to publish it in Nature Genetics, pending minor revisions to satisfy the referees' final requests and to comply with our editorial and formatting guidelines.

If the current version of your manuscript is in a PDF format, please email us a copy of the file in an editable format (Microsoft Word or LaTex)-- we can not proceed with PDFs at this stage.

We are now performing detailed checks on your paper and will send you a checklist detailing our editorial and formatting

requirements soon. Please do not upload the final materials and make any revisions until you receive this additional information from us.

Thank you again for your interest in Nature Genetics Please do not hesitate to contact me if you have any questions.

Sincerely,

Margot Brandt, PhD
Senior Editor
Nature Genetics
https://orcid.org/0000-0002-9434-794X

Reviewer #1 (Remarks to the Author):

The authors provided a detailed response to my comments and carefully revised the manuscript. The revised manuscript contains additional simulations and clarifications, the software includes new options.

Reviewer #1 (Remarks on code availability):

The code is available and, as indicated by the authors, was already used by external researchers. I believe the shared software is highly valuable for the scientific community.

Reviewer #2 (Remarks to the Author):

The author addressed my concern appropriately.

Reviewer #2 (Remarks on code availability):

The guidance and instruction is clear.

Reviewer #3 (Remarks to the Author):

All comments have been addressed.

Reviewer #3 (Remarks on code availability):

commented previously

Open Access This Peer Review File is licensed under a Creative Commons Attribution 4.0 International License, which permits use, sharing, adaptation, distribution and reproduction in any medium or format, as long as you give appropriate

credit to the original author(s) and the source, provide a link to the Creative Commons license, and indicate if changes were made.
In cases where reviewers are anonymous, credit should be given to 'Anonymous Referee' and the source.
The images or other third party material in this Peer Review File are included in the article’s Creative Commons license, unless indicated otherwise in a credit line to the material. If material is not included in the article’s Creative Commons license and your intended use is not permitted by statutory regulation or exceeds the permitted use, you will need to obtain permission directly from the copyright holder.
To view a copy of this license, visit https://creativecommons.org/licenses/by/4.0/

We thank the reviewers and editors for the time and care devoted to evaluating this manuscript. We also appreciate the encouraging and constructive feedback and suggestions received from the broader scientific community following the release of our preprint. In this revised version, we have incorporated the reviewers' comments and provide a point-by-point response (indicated in blue), as well as addressed input from the wider community. We are grateful to everyone whose contributions have helped improve the completeness and quality of this work.

General comments from the broader scientific community:

In response to feedback that the nomenclature was difficult to follow, we have:

1. Used standardised terminology throughout the manuscript as follows:

Method → target data / LD reference (when not using in-sample LD).

We adopt a consistent naming scheme to clarify comparisons. For example, Manc-COJO → 6.9K AFR + 6.9K EUR, rather than simply Manc-COJO, denotes its application to GWAS summary statistics from 6.9K AFR and 6.9K EUR samples using in-sample LD; here, "+" indicates separate GWAS summary statistics and LD reference inputs for each ancestry. By contrast, GCTA-COJO → 6.9K AFR_6.9K EUR / 75.9K EUR LD indicates that the LD reference differs from the GWAS summary statistics and is therefore specified explicitly, with "_" denoting that the GWAS summary statistics of two ancestries has been combined.

2. Revised single ancestry terminology (Sanc-COJO and GCTA-COJO)
As the Manc-COJO software also supports single-ancestry analyses, the use of "Sanc-COJO" to refer to GCTA-COJO may be misleading. We therefore use GCTA-COJO to refer to analyses performed using the original GCTA-COJO software. Our implementation of single ancestry COJO within Manc-COJO—identical to GCTA-COJO except for the bug fix described in Supplementary Notes 4—is now referred to as Sanc-COJO.

Reviewers' Comments:

Reviewer #1 (Remarks to the Author):

Wang et al. introduce Manc-COJO, a method for multi-ancestry COJO analysis. They also introduce Manc-COJO:MDISA, a follow-up approach to identify ancestry-specific associations. Their methodology is implemented in an efficient tool that is computationally fast and enables genome-wide analyses. The manuscript is well written, statistical features are discussed in detail, limitations are discussed, and extensive

simulation studies and real data analyses illustrate and investigate the new methodology. The results show that Manc-COJO outperforms existing approaches in suitable scenarios and has potentially important applications in the investigation of GWAS results from meta-analyses across multiple ancestries.

• In an admixed study population where distinct ancestral groups cannot be clearly separated, and individual-level genotype data are available, is it appropriate to perform the analysis as a single-ancestry study using an LD matrix estimated from the full sample? Or what would be the recommendation here?

Yes, in admixed populations, it can be appropriate to conduct the analysis as a single-ancestry study, as shown in a previous study[1]. However, as discussed in the **Supplementary Note 6**, several important caveats apply: in-sample LD (as noted by the reviewer) must be used, and for some SNPs, allele frequencies can no longer be approximated by 2*MAF*(1-MAF) due to violations of Hardy–Weinberg equilibrium induced when differences in allele frequencies between parent/source populations are large.

In response to reviewer comments, as well as broader community needs, we have implemented a new function to support analyses in admixed populations. This function (*--var-from-LD*) directly estimates genotypic variance from LD reference panels, rather than relying on the 2*MAF*(1-MAF) approximation. It is notable that the purpose of -var-from-LD is to enable COJO analyses using GWAS summary statistics and an LD reference derived from the same set of samples, approximating the scenario in which individual-level genotype data are available. The method does not correct for deficiencies in the upstream analytical pipeline used to generate the summary statistics or LD reference. For example, SNP correlations may reflect not only LD but also population stratification; if factors such as in-sample PCs are not properly accounted for in the GWAS, or if the LD reference does not closely match the genomic ancestry proportions of the study sample, the resulting outputs will be unreliable. Users should therefore exercise extra caution when applying the *--var-from-LD* option and carefully consider the assumptions underlying their analysis.

We have expanded Supplementary Note 6 to provide additional background. In the discussion of the main text (line 346), we direct readers to this section: "Although this study applies meta-analysis strategies to integrate data across ancestries, the software can also be adapted to perform mega-analysis ...... or analyses within admixed populations ...... (**Supplementary Note 6**)."

• How would a user decide to run Manc-COJO:MDISA as an additional analysis? Or is it

best practice to always run Manc-COJO and Manc-COJO:MDISA? Steps 1-8 in the Methods section suggest that it is an integrated part of the analysis.

We thank the reviewer for raising this point. The original aim of developing MDISA was to identify ancestry-specific signals, where present, and our simulations suggest that it can recover part of the association signal when such variants truly exist. However, in analyses of real data, we find that most variation is well explained by models assuming shared causal effects, and the additional variants identified account for only a very small proportion of phenotypic variance.

Moreover, we cannot exclude the possibility that the additional association signals detected by Manc-COJO:MDISA reflect differences in statistical power, given that current GWAS are predominantly based on European-ancestry samples, rather than true ancestry-specific causal variants. For example, a causal variant that is shared across ancestries may be underpowered for detection in AFR due to smaller sample sizes, leading to imprecise effect estimates. As a result, the variant may be non-significant in AFR, significant in EUR, but fail to reach significance in the meta-analysis due to the added noise from AFR. In such cases, the variant may later be detected in EUR-specific analyses and incorrectly interpreted as EUR-specific despite being shared. This issue is further compounded by the steeper LD decay in AFR populations. At current SNP probe densities, the signal from true causal variants tends to attenuate more rapidly at nearby markers than in EUR, thereby further reducing power in AFR.

Therefore, we recommend **against** using MDISA as an integrated part of routine analyses unless cohorts across ancestries are sufficiently well powered and SNP density is high. In this way the researcher actively considers their analyses. We believe we have provided a method that will identify ancestry specific variants if they exist, and that as sample sizes increase the results will demonstrate whether they exist or not.

Notably, the MDISA function can be applied in broader scenarios, such as fixing variants already validated (e.g., through wet-lab experiments). We therefore recognise that the name “MDISA” may be misleading. Accordingly, we decided to rename the function as “FIX” and to extend its scope (with the original MDISA representing a special case of FIX). We have revised the Methods and Results sections accordingly and added **Supplementary Notes 3** to describe the use of this function in detail.

• Some meta-analyses incorporate ancestries with a relatively small sample size. What is a lower sample size cutoff to get reasonable results?

To provide readers with a general sense of whether ancestries with relatively small sample sizes should be included, we conducted a series of simulations based on

Equation 19 (**Supplementary Note 1.2.3**) and added **Supplementary Note 7.2** to describe these analyses in detail.

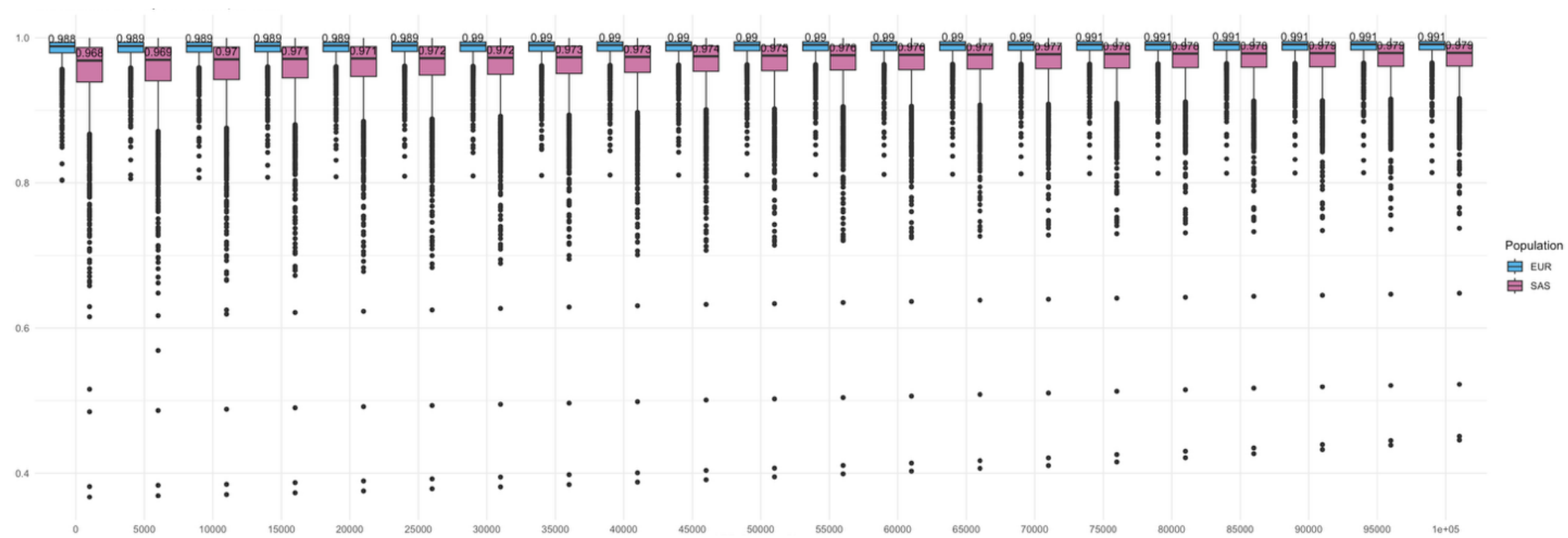

(a)

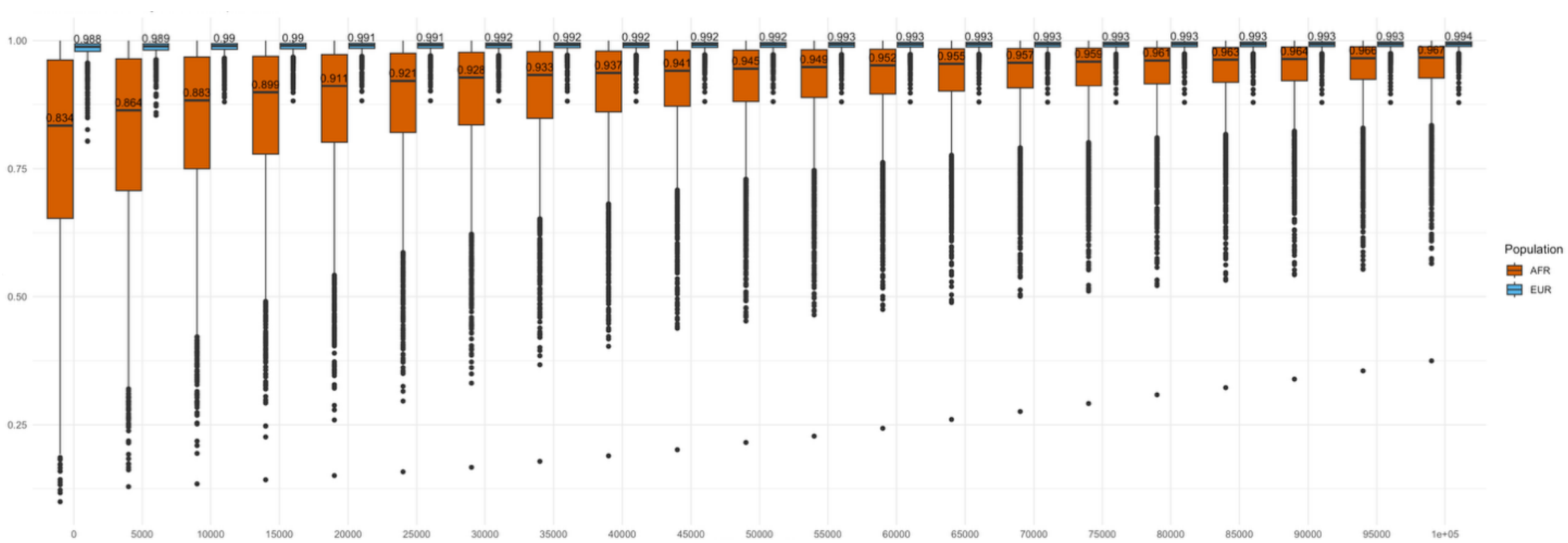

(b)

Supplementary Fig. 9. Impact of adding a second ancestry with limited sample size on the identification of independent associations. Simulations based on equation (19) in Supplementary Note 1.2.3 were conducted to evaluate whether incorporating ancestries with relatively small sample sizes improves the identification of causal variants. In a two ancestry setting, the EUR sample size was fixed at 0.5 million, while the SAS (x axis of panel a) or AFR (x axis of panel b) sample size increased from 0 to 0.1 million. Performance was assessed using the mean LD r^2 between the top associated SNP and the true causal variant, averaged across 10,000 simulations. The plots show the distribution of mean LD r^2 values (y axis) across all SNPs when each SNP is treated as the causal variant. Panels a and b show that adding an additional ancestry improves the ability of COJO to resolve independent signals, although the benefit is modest when the added sample size is small. This limitation is more pronounced for the EUR and SAS combination, consistent with their longer range LD structure compared to AFR.

Overall, incorporating ancestries with modest sample sizes (e.g. 5,000) can help to refine LD structure, particularly when including ancestries with shorter LD blocks, such as African ancestry populations. However, because our algorithm operates on SNPs shared across all cohorts, the inclusion of a cohort with both a small sample size and limited SNP overlap may be detrimental, as it reduces the number of variants available for cross-ancestry analysis.

Defining a universal threshold is challenging, as the reliability of results depends on allele frequency, effect size and the underlying genetic architecture, all of which vary across traits and variants. We are developing a tool to assist with these decisions and to inform

sample size requirements across ancestries; however, this work is beyond the scope of the present study and will be reported separately.

At present, we recommend considering the inclusion of additional ancestries with smaller sample sizes when: (1) SNPs available for analysis show strong overlap with other datasets; (2) data collection and analytical pipelines are harmonised, as in large international consortia; and (3) high-quality individual-level genotype data are available to construct reliable LD reference panels. The original GCTA-COJO paper showed that an LD reference panel with more than 5,000 individuals generally provides reliable LD estimates. Ideally, in-sample LD should be used (i.e., the GWAS cohort itself has a sample size of at least 5,000). Alternatively, LD estimates may be derived from an external cohort drawn from the same ancestral population so that the LD structure is well-matched to that of the GWAS cohort; in this case, the GWAS sample may be smaller, although—as illustrated in the figure—the gains are expected to be limited when the GWAS sample size falls below 5,000.

Conversely, if any of these conditions are not met, then including small cohorts may introduce more noise than benefit. In such cases, empirical evaluation (e.g., model size and out-of-sample predictive performance) should guide decisions about whether to include a given cohort. We have included these discussions in **Supplementary Note 7.2**.

• Does the software output heterogeneity measures for effect sizes across ancestries?

We thank the reviewer for this suggestion and agree that such statistics are useful for assessing data quality. We have added a `--hetero_report` flag to report Cochran's Q statistics for joint effect sizes across ancestries.

• I appreciate the discussion about the fine mapping abilities of the Manc-COJO approach. I wonder if the authors could compare their approach with recent multi-ancestry fine-mapping approaches such as MultiSuSiE or MESuSie and discuss in more detail what the advantage of using COJO is compared to performing fine-mapping.

COJO and SuSiE are best viewed as complementary rather than competing approaches. As noted in the main text, COJO is not, strictly speaking, a fine-mapping method, although we recognise it is often (inappropriately) used for this purpose.

Within the COJO framework, a single SNP is typically selected to represent each independent signal in a region, with the primary objective of estimating the number of conditionally independent associations. By contrast, SuSiE and related Bayesian methods

assign posterior probabilities to variants, explicitly capturing uncertainty and allowing the definition of credible sets enriched for putative causal variants.

For fine-mapping purposes, COJO is often overly parsimonious, as it selects too few variants. Conversely, Bayesian approaches such SuSiE tend to include a larger set of candidate causal variants, which improves sensitivity.

Given that our implementation of COJO can complete WGS-scale analyses within minutes, a practical strategy is to use COJO to identify candidate regions, followed by Bayesian fine-mapping methods to refine causal inference. Further details are provided in **Supplementary Notes 8**, and we have included the key figure below.

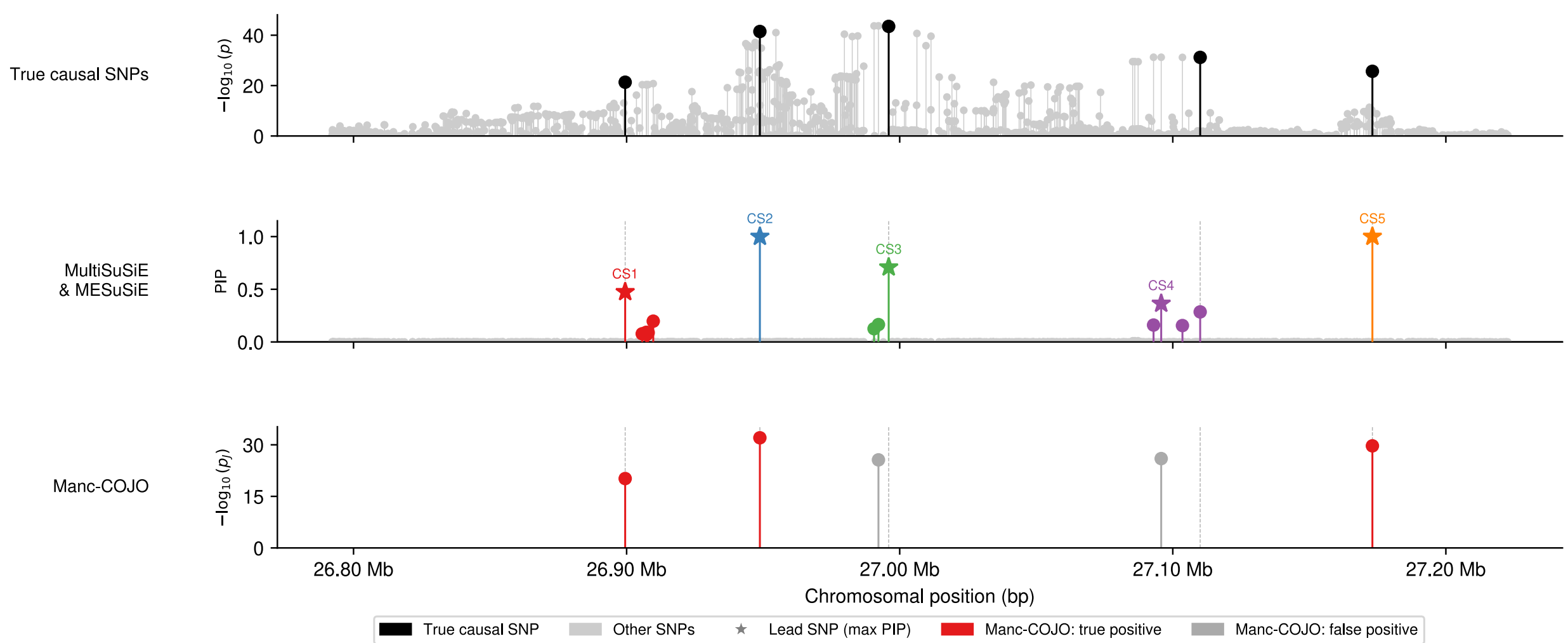

Supplementary Fig. 11. Locus visualisation of fine-mapping and COJO analysis results for a representative simulation replicate. Each panel displays SNPs along a ~400 kb region of chromosome 22. (Top panel) Marginal association signals for all SNPs, where y-axis represents $-\log_{10}p$ with p denoting the marginal GWAS p-value from the combined multi-ancestry summary statistics. True causal SNPs are highlighted in black; all other SNPs are shown in grey. (Middle panel) Posterior inclusion probabilities estimated by MultiSuSiE and MESuSiE, which produced identical credible sets in this replicate. Each credible set (CS1–CS5) is shown in a distinct colour; the star marker denotes the lead SNP (maximum PIP within the credible set) and circles denote other credible set members. y-axis represents the PIP of each SNP, reflecting the posterior probability that the SNP is causal. Dashed vertical lines indicate the positions of true causal SNPs. (Bottom panel) SNPs selected by Manc-COJO, where y-axis represents $-\log_{10}p_J$, with p_J denoting the joint p-value obtained from stepwise selection. Red markers indicate true causal SNPs identified, whereas grey markers denote selected SNPs that are not causal. This difference reflects the distinct objectives of the methods. Fine mapping approaches such as MultiSuSiE and MESuSiE model all SNPs jointly and report credible sets that capture uncertainty, often including correlated variants. In contrast, COJO performs stepwise conditional analysis to identify a parsimonious set of approximately independent association signals, where tagging a SNP in linkage disequilibrium with the causal variant is typically sufficient.

- 'First, we made several assumptions: true causal SNPs act additively on the phenotype, causal SNPs are independent, and the number of true causal variants, as well as their effect sizes on standardised phenotypes, are identical across ancestries.' Can the authors clarify the assumption that the causal SNPs are independent?

Thanks for pointing this out. Our original intent was to assume that the **effect sizes** of causal variants are independent. However, we agree that the wording in the main text regarding the independence of causal SNPs was unclear and incomplete. We have therefore revised the wording in the main text (line 354) in line with wording used in the

GCTA-COJO paper: “First, we made several assumptions: true causal SNPs act additively on the phenotype, and causal effects are treated as fixed effects…”. We have also updated Section 1.1.1 of the Supplementary Methods accordingly.

• I think the methodology can incorporate varying sample sizes across SNPs; can the authors emphasize and discuss this in the text? (PMID: 36643910)
Yes, the current COJO algorithm does accommodate varying sample sizes across SNPs (as did GCTA-COJO). Approaches for handling missing data are described in Supplementary Notes 4.3, reflecting strategies previously adopted in the literature. We agree with the reviewer that additional strategies may be beneficial, such as applying standard pre-processing quality control to summary statistics and removing suspicious variants prior to COJO analysis using tools such as SLALOM[2] or DENTIST[3]. We have therefore expanded the discussion in Supplementary Notes 4.3 to outline how these tools can be applied in the current framework “Meanwhile, the input GWAS summary statistics can be adjusted to mitigate missingness…..specialised tools such as SLALOM and DENTIST can be used to identify loci exhibiting suspicious heterogeneity across cohorts, which has been shown to improve downstream analyses such as fine-mapping.”

Reviewer #1 (Remarks on code availability):

The GitHub repository contains usage instructions and example data. While I have not run the software myself, the materials provided suggest that reproducing the results described in the manuscript should be feasible.

Thank you for checking our code. Since posting our manuscript on a pre-print server many people have tried our software tool. In general, evaluations are very good, and based on feedback we have continued to improve user experience.

Reviewer #2 (Remarks to the Author):

This paper introduces a new method Manc-COJO, which is a multi-ancestry extension of the classical COJO method, aimed to identify conditionally independent GWAS signals using ancestry-specific LD and summary statistics. The author gave theoretical analysis and developed an efficient software with C++ to implement the proposed method. By conducting extensive simulations and real data analysis to a couple of traits in the paper, the author demonstrates the advantage of the proposed method over the existing COJO framework and other ad hoc methods. Overall, the paper is technically strong, well-organized and clearly written.

Below are some of my concerns:

1.The Manc-COJO utilized the stepwise selection method (forward/backward), how will the author assess the selection stability? How sensitive are the results to the chosen p-value threshold? Some discussions to address the above questions might be helpful.

We thank the reviewer for this suggestion and agree that this distinction warrants explicit clarification.

Briefly, the COJO framework is deterministic, in contrast to Bayesian approaches that rely on stochastic procedures such as MCMC sampling. In addition, COJO employs a greedy algorithm, such that the order of SNP screening does not influence the final results. Consequently, identical outputs are obtained given the same input data and parameter settings.

Accordingly, the principal determinants of the results are the input data and parameter choices. With respect to the former, this is directly related to Reviewer 1's question regarding the impact of including a small cohort, which we now address in the **Supplementary Note 7**.

Another key parameter, as noted by the reviewer, is the p-value threshold. Different thresholds reflect distinct analytical priorities: more stringent thresholds favour the identification of secondary (independent) associations with fewer false positives, at the cost of reduced statistical power, whereas less stringent thresholds increase power but permit a higher false-positive rate.

In our view, less stringent thresholds may be advantageous for downstream applications such as improving predictive performance, although Bayesian methods (e.g., SBayesRC) are generally better suited for this purpose. By contrast, when the objective is to identify secondary associations, we favour more stringent thresholds. We have added a corresponding discussion in **Supplementary Notes 9**.

2.The author demonstrates the superiority of the proposed method with two different ancestries, how would the author expect the performance if more ancestries are included? As the sample size between the ancestries might be imbalanced, for example, if Eur>>Afr>>South Asia, will the extremely small sample size of the third population affect the performance of the other two in the proposed method?

Theoretically, including additional ancestries can improve the ability to detect independent associations, as LD structure varies across populations. In particular, ancestries with shorter LD—such as AFR populations—provide greater resolution for disentangling correlated signals. In contrast, ancestries with generally longer LD blocks

contribute less additional resolution, although they may still offer more information than using EUR samples of equivalent size alone. We have added a simulation in the **Supplementary Note 7** to illustrate this effect (key figures are provided below).

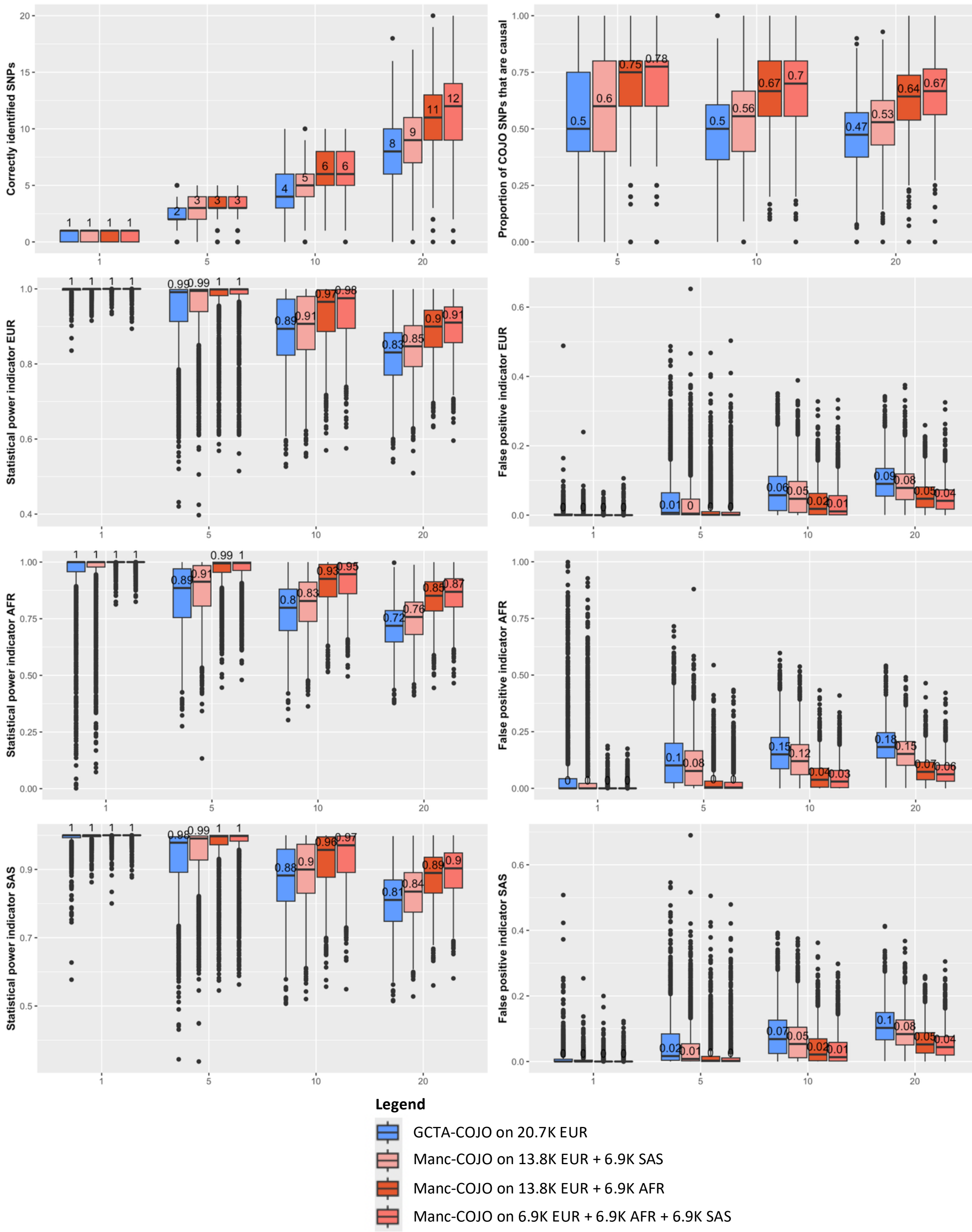

Supplementary Fig. 8. Benefit of incorporating additional ancestries into the analysis. This figure summarises simulation results from 7,600 scenarios and demonstrates the benefit of incorporating well powered data from additional ancestries in increasing statistical power and reducing false positive discoveries when identifying independent associations. The simulated scenarios are described in Supplementary Note 8.1. The layout of this figure is identical to Fig. 2, except that the evaluation metrics, power (bottom left panel) and false positives (bottom right panel), were additionally calculated using linkage disequilibrium patterns in SAS. NB The total sample size in all analyses is higher here than in Fig. 2.

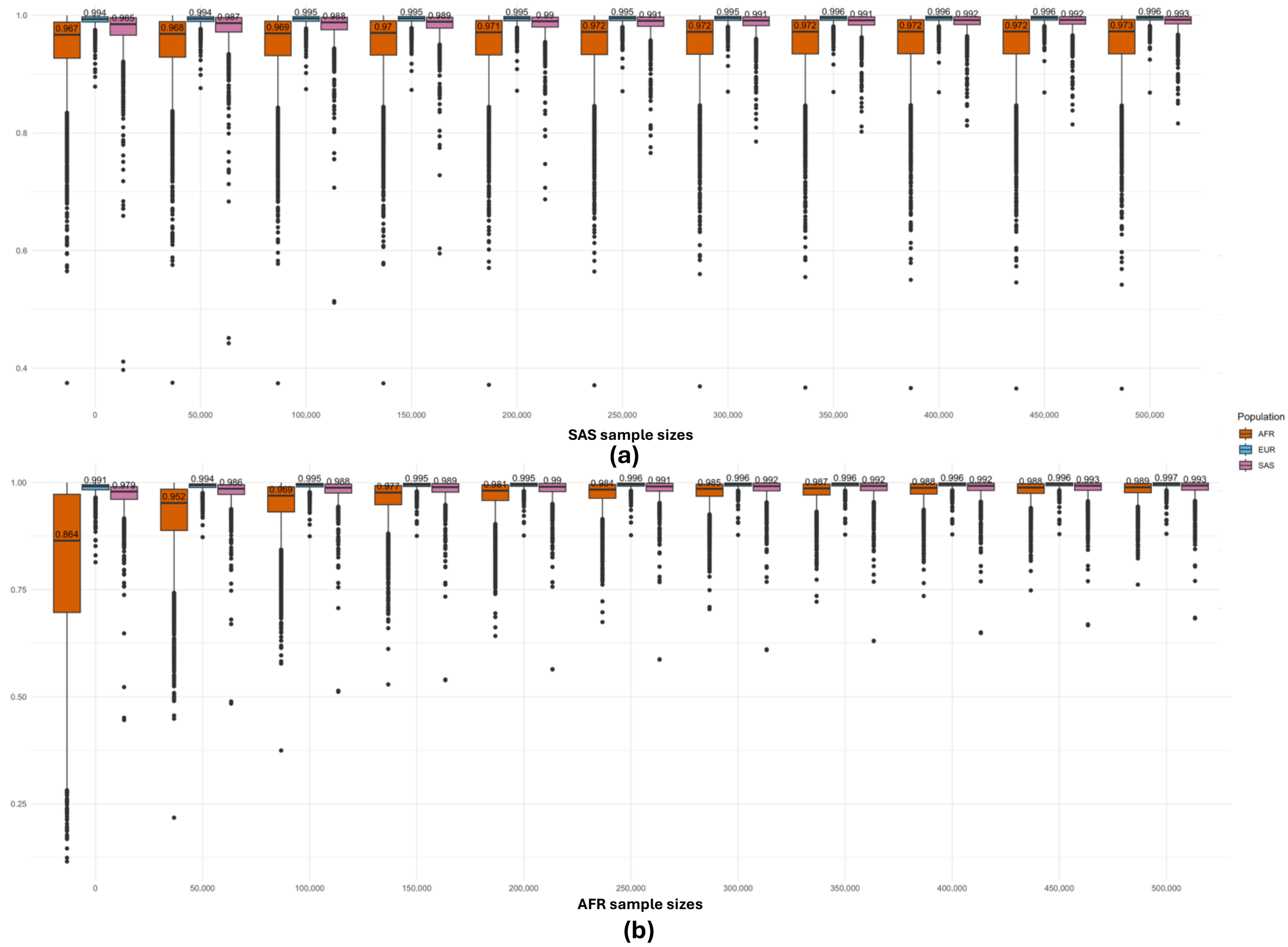

Supplementary Fig. 10. Impact of adding a third ancestry with limited sample size on the identification of independent associations. Simulations based on equation (19) in Supplementary Note 1.2.3 were conducted to evaluate whether incorporating a third ancestry improves the identification of causal variants. The EUR sample size was fixed at 0.5 million. In panel a, the AFR sample size was fixed at 0.1 million while the SAS sample size increased from 0 to 0.5 million (x-axis). In panel b, the SAS sample size was fixed at 0.1 million while the AFR sample size increased over the same range. Performance was assessed using the mean LD r^2 between the top associated SNP and the true causal variant, averaged across 10,000 simulations. The plots show the distribution of mean LD r^2 values (y axis) across all SNPs when each SNP is treated as the causal variant. Panels a and b show that increasing the sample size of the third ancestry improves the ability of COJO to resolve independent signals, with selected variants showing stronger correspondence to the true causal SNP. This improvement is more pronounced when increasing AFR sample size, consistent with its shorter LD structure compared to SAS.

In practice, however, the main challenge when incorporating multiple ancestries is not limited sample size, but differences in SNP coverage across datasets. Variants may not fully overlap between ancestries, leading to reduced SNP density after harmonization. This reduction in marker density can adversely affect model performance. This caveat is noted in **Supplementary Note 7**.

In the main text (line 344) we have added "Consequently, there is a trade-off between increasing sample size and ancestral diversity as including more datasets may reduce the number of SNPs available for analysis. Data input decisions are described in detail in Supplementary Note 7." to refer to Supplementary Note 7.

Reviewer #2 (Remarks on code availability):

The author has clearly tutorial and guidance on how to install and use the software on

Github.

Thanks for checking.

Reviewer #3 (Remarks to the Author):

The popular GCTA-COJO method for conditional and joint analysis within a single ancestry is extended to multiple ancestries, Manj-COJO. Importantly, the authors also introduce Manc-COJO:MDISA, which identifies ancestry-specific variants that would be missed by Manc-COJO. A faster COJO implementation is also provided in their new software.

Various simulation settings involving two ancestries are explored, including sensitivity analyses.

Some minor comments

1. The method overview should clearly state that Manc-COJO only considers variants that are present in all ancestries.

We thank the reviewer for this comment and have incorporated this clarification into the Discussion (line 342): "Notably, Manc-COJO operates on variants, which association statistics (e.g., effect size, standard error and sample sizes) are available across all ancestries."

2. When there are more than two ancestries, is it possible to identify variants that are present in and associated with a subset of the ancestries (e.g. variant present in European and East Asian and associated with the trait in both ancestries, but the variant is rare in African so not present in the African GWAS)?

Thank you for this interesting comment. It motivated us to make the original MDISA function more flexible and to replace it with the new Manc-COJO:FIX implementation. Due to the combinatorial complexity (e.g., with four ancestries, there are $2^4=16$ possible combinations), we do not provide this analysis as a default output. However, users can readily fix the SNPs identified in the all-ancestry model and then perform follow-up analyses within any desired subset of ancestries. Details are provided in the **Supplementary Note 3**.

Reviewer #3 (Remarks on code availability):

Clear installation instructions are provided, together with a tutorial using example data.

Thanks for checking.

We again thank all reviewers for their valuable feedback on the manuscript.

References

1. Lee, H., Lee, M.H., Hou, K., Pasaniuc, B. & Han, B. Admixed and single-continental genome segments of the same ancestry have distinct linkage disequilibrium patterns. *Genome biology* **26**, 201 (2025).
2. Kanai, M. *et al*. Meta-analysis fine-mapping is often miscalibrated at single-variant resolution. *Cell genomics* **2**(2022).
3. Chen, W. *et al*. Improved analyses of GWAS summary statistics by reducing data heterogeneity and errors. *Nature Communications* **12**, 7117 (2021).
